# Tuning Photophysical and Chiroptical Properties of [7]Helicene through Formation of Imidazole-Based Push–Pull Systems

**DOI:** 10.1021/acs.joc.6c00092

**Published:** 2026-05-19

**Authors:** Martin Kos, Tomáš Beránek, Sachika Takase, Jaroslav Žádný, Jan Sýkora, Ivana Císařová, Jan Storch, Vladimír Církva, Martin Jakubec

**Affiliations:** ∥ Department of Materials Chemistry, Research Group of Advanced Materials and Organic Synthesis, 86876 Institute of Chemical Process Fundamentals of the Czech Academy of Sciences, v. v. i., Rozvojová 135, 165 00 Prague 6, Czech Republic; ‡ Department of Analytical Chemistry, University of Chemistry and Technology Prague, 166 28 Prague 6, Czech Republic; § Department of Inorganic Chemistry, Faculty of Science, Charles University in Prague, Hlavova 2030, 128 40 Prague 2, Czech Republic

## Abstract

Although carbohelicenes are attractive circularly polarized luminescence (CPL) emitters, they often suffer from low fluorescence quantum yields and relatively modest luminescence dissymmetry factor (*g*_*lum*_), resulting in limited CPL brightness. Here, we report the modulation of the photophysical and chiroptical properties of [7]­helicene through the formation of imidazole-based push–pull systems. A series of diaryl imidazole derivatives bearing electron-donating and electron-withdrawing substituents was synthesized, and selected compounds were obtained in enantiomerically pure form. Imidazole incorporation markedly enhances fluorescence quantum yields and molar absorption coefficients, while preserving blue emission (λ_em_ ≈ 452–457 nm). Although absorption and luminescence dissymmetry factors decrease compared to the parent helicene, the combined increase in molar attenuation coefficient at the excitation wavelength (ε) and fluorescence quantum yields (Φ) leads to a substantial enhancement of CPL brightness. The best-performing derivatives exhibit up to a 15-fold increase in CPL brightness (*B*_*CPL*_) relative to unsubstituted [7]­helicene. These results identify imidazole-based push–pull functionalization as an effective strategy for improving the overall CPL performance of helicenes.

## Graphical abstract

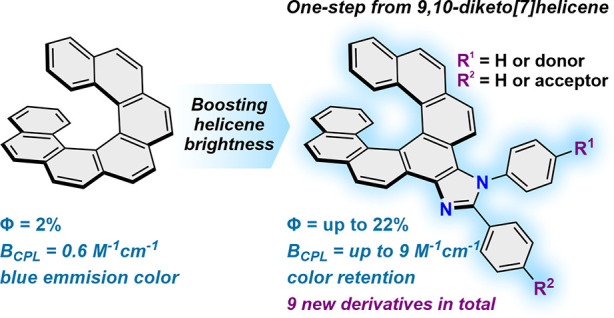

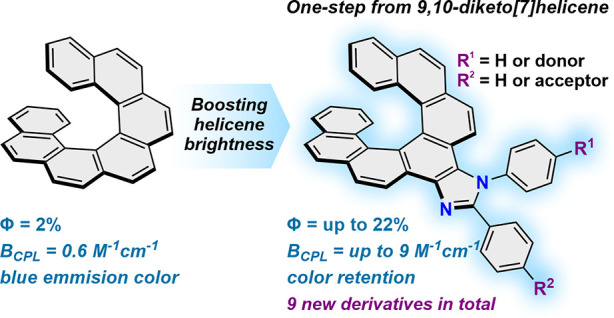

## Introduction

Chiral polyaromatic materials have recently attracted growing attention due to their unique structural and photophysical properties. By incorporating a chiral moiety into an otherwise achiral polyaromatic scaffold, a range of new chirality-driven functionalities^1–3^ emerges from the interplay between inherent chirality and extended π-conjugation, enabling potential applications in nonlinear optics, switches and sensors, spin filtering, and other fields.^4^ Among these, the ability to emit circularly polarized light (CPL) stands out as particularly significant, positioning such molecules as promising candidates for next-generation optoelectronic devices,^5,6^ quantum information processing,^7,8^ and advanced data encryption technologies.^9^

Within this field, helicenes represent one of the most accessible and versatile families of chiral polycyclic aromatic hydrocarbons (PAHs). These helically chiral scaffolds combine inherent structural chirality with tunable photophysical properties, offering an attractive platform for CPL-active materials. However, despite their appeal, simple carbohelicenes and many of their functionalized derivatives are limited by intrinsically low fluorescence quantum yields (Φ, typically <0.05), largely due to efficient nonradiative intersystem crossing (ISC),^10^ as well as modest luminescence dissymmetry factors (*g*_*lum*_ values typically in the 10^–4^–10^–3^ range). Together, these limitations translate into poor CPL brightness (*B*_*CPL*_), defined as *B*_*CPL*_ = *ε* × Φ × *g*_*lum*_/2, where *ε* is the molar attenuation coefficient at the excitation wavelength.

Considerable efforts have therefore been directed toward overcoming these deficiencies, and a wide range of design strategies have been explored. These include the incorporation of heteroatoms,^11–14^ backbone elongation,^15,16^ π-extension,^17–19^ and symmetry modification,^20–22^ each of which can selectively influence one or more photophysical parameters. For instance, π-extension of helicenes, as demonstrated in molecules **Ia** and **Ib** (Figure 1), produced a simultaneous increase in both quantum yield and dissymmetry factor. Despite a slight reduction in *ε*, the overall CPL brightness improved more than 11-fold.^16^ In another approach, assembling multiple helicene units within a single molecule enhanced the dissymmetry factor, as observed in compounds **IIa** and **IIb**.^23^ Interestingly, while **IIb** displayed a 2.2-fold higher *g*_*lum*_ relative to **IIa**, its markedly reduced fluorescence quantum yield diminished the overall brightness. Functional group modification provides yet another pathway: molecules **IIIa** and **IIIb**, bearing triarylamino- and triarylborane substituents at the sterically demanding 2- and 2′-positions, exhibited a remarkable 7.5-fold enhancement in *g*_*lum*_.^24^ However, this came at the cost of significant emission red-shift, due to the formation of donor–acceptor charge-transfer (CT) states.

**Figure 1:**
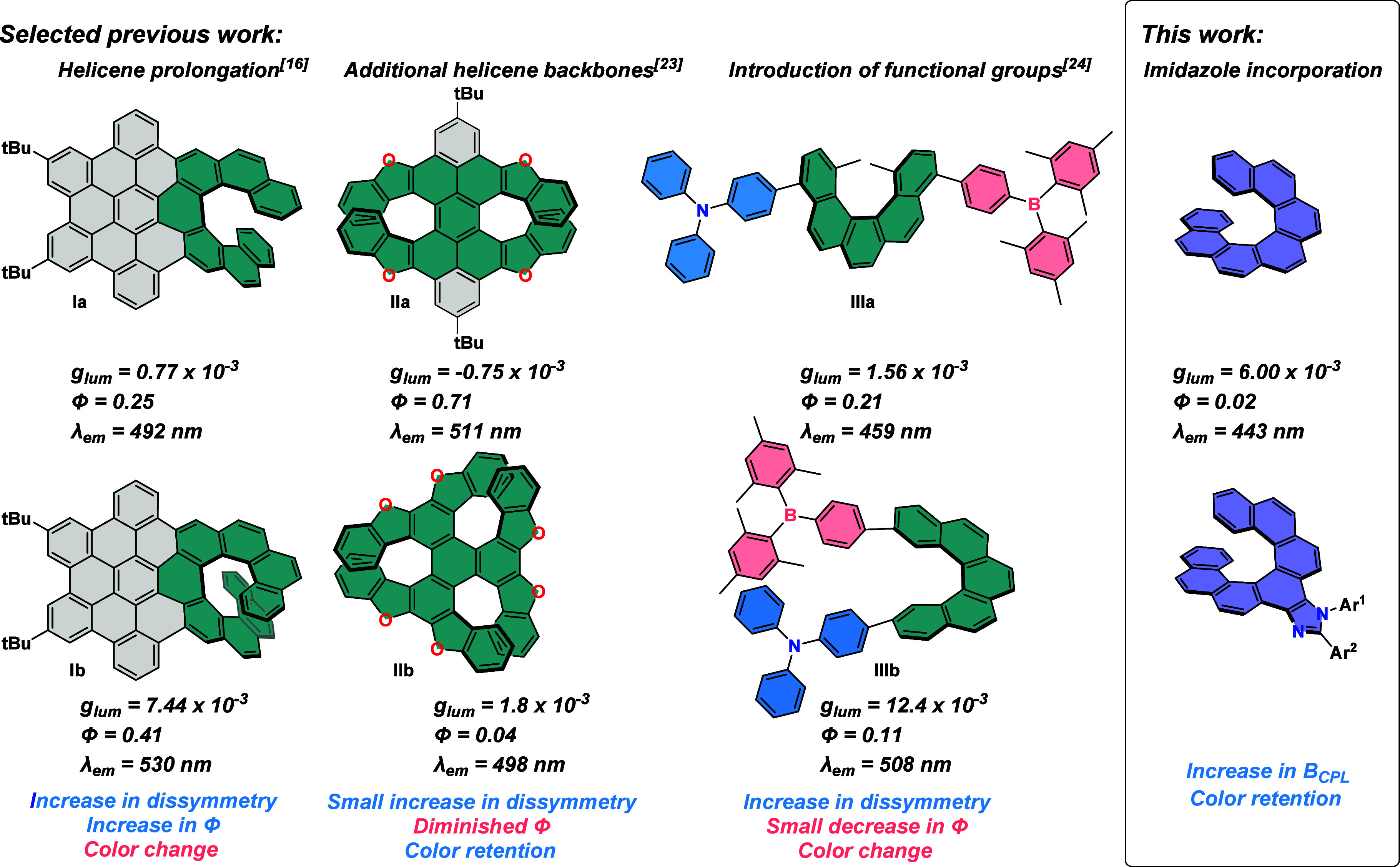
Previously published examples of helicene-based push–pull systems and this work.

These representative cases highlight the central challenge in designing efficient CPL emitters: enhancing one photophysical parameter often comes at the expense of another. Achieving simultaneous improvements in quantum yield, dissymmetry factor, molar absorption coefficient and, subsequently, brightness, while also retaining the desired emission color, remains a challenging task.

In this work, we investigate the changes in the photophysical parameters, such as *ε*, *g*_*lum*_, fluorescence quantum yield, or emission color of the parent [7]­helicene upon introduction of emissive imidazole-based push–pull systems. To achieve this, we employ a modification of our previously published method for synthesizing helicene imidazoles from 9,10-diketo[7]­helicene,^25^ which also enables straightforward access to its enantiomerically pure versions.^26^

## Results and Discussion

The rationale behind this molecular design is 3-fold: a) we have previously observed emergence of bright blue fluorescence in helicenes upon condensation of imidazole moiety;^25^ b) utilization of push–pull systems in helicenes leads to higher Φ^27^ and; c) imidazoles of similar design were already used in blue OLED fabrication.^28,29^ We therefore proposed a library of nine new diaryl imidazoles bearing both electron donors and electron acceptors (Scheme 1). During the optimization of the reaction conditions of the Debus–Radziszewski imidazole synthesis, we scanned a variety of substituents on both carbaldehydes and anilines in the presence of ammonium acetate and acetic acid. While the substituents on carbaldehyde had little to no impact on the yield of the reactions and were always incorporated into the imidazole, electron acceptors (such as the trifluoromethyl group) on the anilines decreased the nucleophilicity of the amine and instead, ammonia coming from the ammonium acetate was incorporated, yielding an undesired monoaryl N–H imidazole. As a result, we decided to introduce the electron donors on the anilines, while the electron acceptors were introduced into the carbaldehyde (Scheme 1). The reaction was run at 100 °C and typically provided full conversion after approximately an hour. The yields of this reaction typically ranged from 72 to 90% with the exception of **2-CF**_**3**_ (59%), and under these conditions, a minimal amount of the monoarylated product was observed. The identity was unambiguously confirmed by a single crystal X-ray analysis for compounds **1-H**, **2-H**, **3-H** and **1-CN** (see SI).

**Scheme 1:**
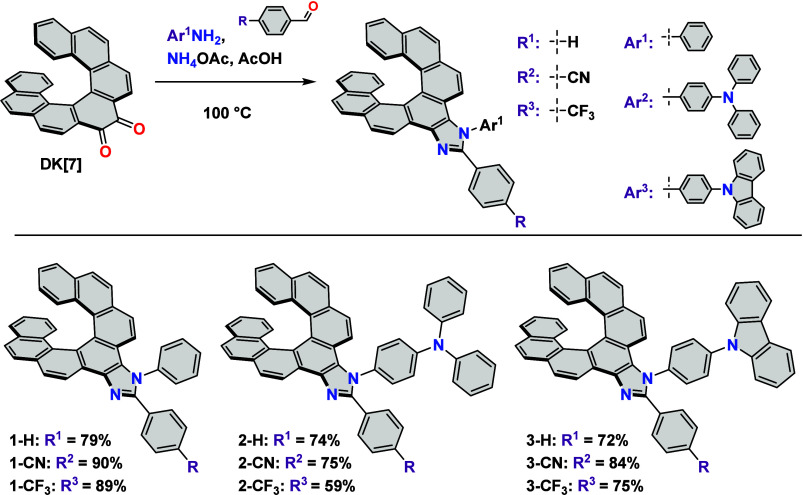
Preparation of the Imidazole Series **1**–**3**

We next investigated the photophysical properties of the prepared helicene derivatives. The UV/vis absorption spectra revealed no major changes upon introduction of either electron-donating or electron-withdrawing substituents. In all cases, the spectra featured a dominant band at around 275 nm and a secondary shoulder or peak in the 315–350 nm region, with the exact position depending on the substitution pattern (see SI). The substituents had an observable impact on the ε, which will be discussed later in the text together with its implications for *B*_*CPL*_. Despite these variations in absorption profiles, the emission maxima of all compounds remained remarkably consistent, falling within a narrow 5 nm range between 452 and 457 nm (Table 1). In contrast, the substitution pattern exerted a much stronger influence on the fluorescence quantum yields (Φ). Across the series, electron-withdrawing groups introduced via the benzaldehyde precursor led to clear enhancements. The effect was modest in the case of the trifluoromethyl substituent, but pronounced in the nitrile-substituted series, where quantum yields increased by up to 41% relative to the unsubstituted analogues (**3-CN** vs **3-H**). Electron-donating diarylamine substituents had a weaker impact: a 14% increase was observed for the diphenylaminophenylene derivative (**2-H** vs **1-H)**, whereas in **3-H** the quantum yield decreased slightly. Notably, systems combining donor and acceptor groups (**2-CN**, **2-CF**_**3**_, **3-CN**, and **3-CF**_**3**_) displayed synergistic effects, exhibiting higher Φ values than any of the corresponding monosubstituted compounds. The highest quantum yields were measured for **2-CN** (22.1%) and **3-CN** (21.6%), representing a 13-fold enhancement compared to the parent [7]­helicene (1.7% measured, 2.0% reported^30^), while crucially preserving the blue emission.

**Table 1:** Optical and Electronic Properties of the Prepared Compounds

	LUMO	HOMO	Δ*E*	ε_340_ ^a^ (M^–1^ cm^–1^)	λ_em_ ^b^ (nm)	Φ_340_ ^c^ (%)	|*g*_abs_^max^|^d^ (×10^–3^)	|*g*_lum_^max^|^d^ (×10^–3^)	*B*_CPL_ (M^–1^ cm^–1^)
**1-H**	–1.848	–5.372	3.524	29 019	456	16.1	2.76	1.79	4.2
**1-CN**	–2.272	–5.598	3.326	36 959	453	19.9	2.29	1.26	5.1
**1-CF**_**3**_	–2.054	–5.545	3.491	28 648	452	14.9			
**2-H**	–1.805	–5.327	3.522	42 569	457	18.3	4.64	2.30	9.0
**2-CN**	–2.212	–5.535	3.323	50 623	452	22.1	1.70	1.47	8.3
**2-CF**_**3**_	–2.000	–5.487	3.487	57 248	453	18.8			
**3-H**	–1.905	–5.434	3.529	38 865	456	15.3	5.09	2.32	6.9
**3-CN**	–2.327	–5.644	3.317	39 586	452	21.6	1.82	1.46	6.3
**3-CF**_**3**_	–2.108	–5.594	3.486	12 530	452	17.6			
**[7]** ^31^	–1.894	–5.701	3.806	12 256^e^	443^e^	1.7^e^	8.57^e^	5.53^e^	0.6

a Extinction at 340 nm, 10^–5^–10^–4^ M concentration range in acetonitrile, 2 mm optical pathway.

b Excitation wavelengths 340–370 nm, 10^–5^ M concentration range in acetonitrile.

c Corrected absolute quantum yields upon excitation at 340 nm.

d Absolute value as the average of the experimentally obtained data from both enantiomers.

e Experimentally obtained value from (*M*)-[7]­helicene enantiomer.

We have also investigated the compounds using DFT calculations (see SI). They showed that changing the electron donor has almost no effect on the HOMO and LUMO levels, as well as the energy gap, which is around 3.52 eV for all three compounds **1-H**, **2-H**, and **3-H**. Introduction of the acceptor then reduced HOMO and LUMO for both cyano- and trifluoromethyl acceptor, but only for the −CN series it resulted in an approximately 0.2 eV reduction of the energy gap (Δ*E*) (Figure 2). In all the compounds, the HOMO orbital is predominantly located on the helicene and imidazole moieties, irrespective of the substitution pattern. The LUMO orbital, however, is spread more toward the electron-accepting moiety in both the −CN and −CF_3_ series. The S_0_→S_1_ transition is comprised of the HOMO→LUMO transition (53 to 81% contribution), with a minor contribution from a HOMO→LUMO+1 transition, as revealed by TD-DFT calculations (see Figure 2). Furthermore, we also calculated the circular dichroism spectra and *g*_*abs*_. Taken from the transition with the highest oscillator strength in the most red-shifted region, some of the compounds provided promising values in the 10^–2^ region. We also calculated the *g*_*lum*_ values for all compounds, which showed similar values in the series with the same acceptor. The series **1-H** -**3-H** exhibited values 3.76, 3.63, and 3.99 × 10^–3^ respectively. Similar behavior was found for the other series as well, although the absolute values of *g*_*lum*_ were lower (details in SI, section 7). I). Based on the aforementioned results, we decided to prepare 6 different sets of enantiomers, each maximizing the final brightness in their own way; the series **1-H**–**3-H** was chosen because it exhibited the largest calculated *g*_*lum*_, and the series **1-CN**–**3-CN** was prepared because it showed the largest Φ and ε in the series.

**Figure 2:**
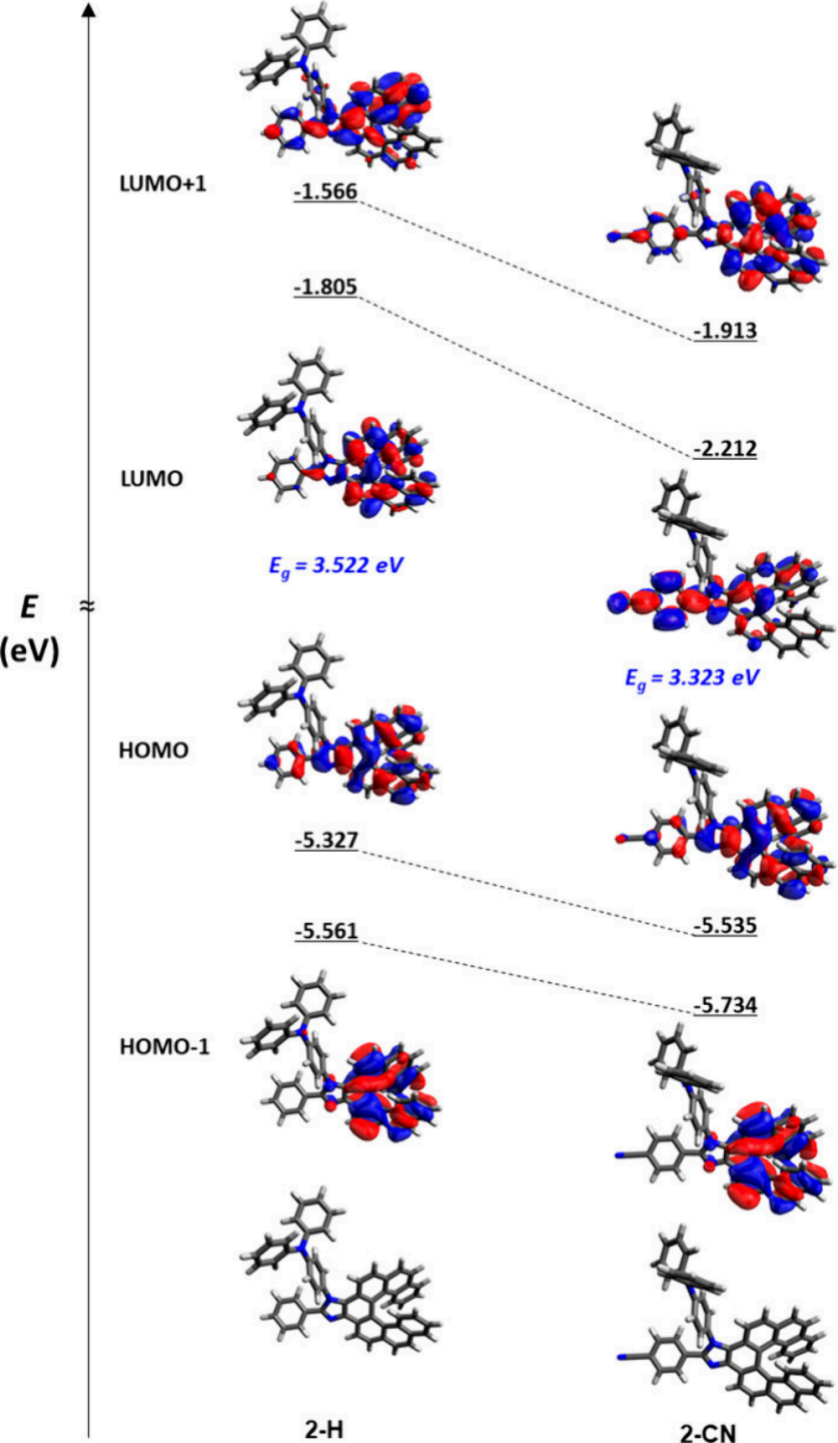
Frontier MOs, energy levels, and Δ*E* for **2-H** and **2-CN** as representative examples of the series. Calculated at B3LYP/6-311G­(d,p)++ level of theory.

The pure enantiomers were prepared from an enantiomerically pure **DK­[7]** (see Scheme 1), which was obtained according to our previously published procedure.^26^ Different yields of the reactions were predominantly attributed to the smaller scale and the necessity to purify the products via column chromatography (see SI). The enantiomeric purity of the products (>98% *ee*) was established using a Chiralpak IC analytical column (see SI), showing that the Debus-Radziszewski reaction proceeds without racemization of the helicene moiety, which is in agreement with the observations we made previously.^26^

Chiroptical characterization was performed using circular dichroism (CD) and circularly polarized luminescence (CPL) spectroscopy. All CD spectra were found to be similar, (*P*)-enantiomers showed a positive Cotton effect at around 360 nm, with a shoulder at approximately 375 nm, and another positive band in the vicinity of 230 nm, which is very typical for (*P*)-enantiomers. The CD spectra of individual enantiomers were also in good agreement with theoretical calculations. Spectra of the same enantiomer also show two negative bands at around 315 and 270 nm. As expected, the (*P*)- and (*M*)-enantiomers display mirror-image CD spectra. Subsequently, the absorbance dissymmetry factors (*g*_*abs*_) were calculated, showing remarkable differences in the two series. While the unsubstituted (**H**) series exhibited *g*_*abs*_ values around 5 × 10^–3^, the cyano-substituted counterparts showed about half the value or less. Given the nominally similar values of Δ*ε* in the CD band of all the compounds, this decrease is attributed to the formation of the push–pull system and the emergence of the stronger absorption band in the 350–400 nm range. It is also evident that the dissymmetry of the molecule can be ascribed mainly to the helicene itself, rather than any of the appended moieties. The CPL spectra of all compounds exhibit clear mirror-image profiles, with *g*_*lum*_ values ranging from 1.56 × 10^–3^ to up to 2.52 × 10^–3^ for individual enantiomers. In general, molecules lacking electron-accepting substituents display higher *g*_*lum*_ values than their cyano-substituted analogues. Conversely, the incorporation of both amine donor groups results in an approximately 50% increase in *g*_*lum*_, yielding the highest overall values of 2.52 × 10^–3^ and 2.45 × 10^–3^ for compounds **2-H** and **3-H**, respectively (see Figure 3 and Figures S22–S25 in SI).

**Figure 3:**
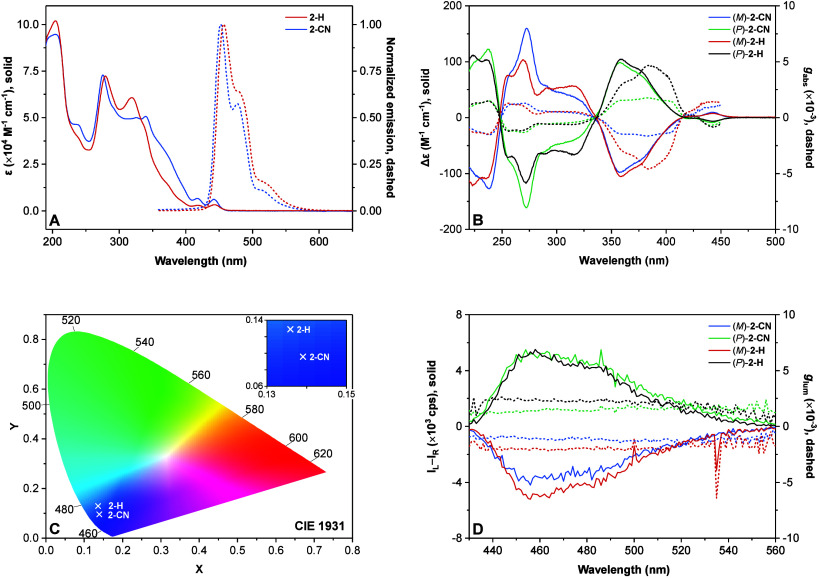
Photophysical data of selected examples **2-H** and **2-CN**. A) UV/vis and fluorescence spectra at 10^–5^ M concentration range in acetonitrile. B) CD spectra and *g*_*abs*_. C) CIE 1931 coordinates of fluorescence emission with zoomed inset. D) CPL spectra and *g*_*lum*_.

These *g*_*lum*_ values, together with the respective *ε* and Φ, were used for the calculation of the CPL brightness (*B*_*CPL*_). The brightness for all compounds was located in a relatively narrow interval from 4.2 to 9.0 M^–1^ cm^–1^. The largest values were found in **2-H** (9.0 M^–1^ cm^–1^) and **2-CN** (8.3 M^–1^ cm^–1^), which represent an impressive 15-fold increase with respect to the unsubstituted [7]­helicene (0.6 M^–1^ cm^–1^) in a single reaction step. The increase in *B*_*CPL*_ is driven by different parameters in different ways. Upon introduction of the imidazole moiety, the *ε* is increased more than twice, with additional increase upon introduction of either the donor, acceptor, or, most significantly, both. Similarly, fluorescence quantum yield has a profound impact as well, increasing almost 9-fold in the basic imidazole derivative **1-H**, and up to more than 13-fold in the cyano-substituted series (vs [7]­hel). The opposite effect was found in the case of *g*_*lum*_, which was reduced by 55–70% in all the prepared compounds, in comparison to the parent [7]­helicene. Interestingly, the *B*_*CPL*_ values within the same series **1**–**3** are almost the same, meaning any changes in *ε* and Φ induced by the introduction of the acceptor are offset by the reduction in *g*_*lum*_. Chosen optical and electronic properties are summarized in Table 1.

## Conclusions

In this paper, we investigated the impact of forming imidazole-based push–pull systems on the CPL performance of the parent [7]­helicene moiety. We found that the imidazole formation strongly improves the fluorescence quantum yield and increases the molar absorption coefficient *ε*, while having only a small impact on the emission color. On the other hand, both *g*_*abs*_ and *g*_*lum*_ are smaller than in the unsubstituted [7]­helicene. Despite that, we were able to increase the overall *B*_*CPL*_ by a factor of 15 in the best performing molecule **2-H**, as well as thoroughly inspect the impact of the individual parameters on the *B*_*CPL*_ *.* We believe that this study showed a new approach toward improving the CPL properties of helicenes and can contribute to design strategies leading to high-performance CPL materials.

## Experimental Section

### Materials and Methods

Commercially available reagent grade materials were used as obtained from Sigma-Aldrich, Acros Organics, Apollo Scientific, and Fluorochem. All solvents were of a reagent grade and used without any further purification, except for tetrahydrofuran and toluene, which were freshly distilled from sodium/benzophenone, and dichloromethane, which was freshly distilled from calcium hydride. TLC was carried out using silica gel 60 F254-coated aluminum sheets, and compounds were visualized with UV light (254 and 366 nm). Column chromatography was performed using Biotage HPFC systems (Isolera One) with prepacked flash silica gel columns. ^1^H and ^13^C­{^1^H} NMR spectra were recorded using Bruker Avance spectrometer at 400 MHz (^1^H NMR), 101 MHz (^13^C NMR), and 376 MHz (^19^F NMR). Chemical shifts (δ) are reported in parts per million (ppm) and referenced to residuals of CDCl_3_ (δ = 7.26 and 77.00 ppm, respectively) or CD2Cl2 (δ = 5.30 and 54.00 ppm, respectively). The coupling constants (*J*) are given in hertz (Hz) and the corresponding multiplicity (s = singlet, d = doublet, t = triplet, m = multiplet). For exact mass measurement, the spectra were internally calibrated using Na-formate or APCI-TOF tuning mix. APCI high-resolution mass spectra were measured in a positive mode using a micrOTOF QIII mass spectrometer (Bruker) and determined by software Compass Data Analysis. The ECD and absorption spectra were recorded on an Olis DSM172 spectrophotometer. The ECD and absorption spectra were recorded over a spectral range of 190 to 650 nm in acetonitrile (10^–5^-10^–4^ M). Absorption was recorded in a quartz cuvette with a 10 mm optical path at constant DIT of 0.1 s, a bandpass width of 1 nm, and a data interval of 0.5 nm. ECD measurements were made in a quartz cell with a 2 mm path length using a fixed bandpass width of 1 nm and variable integration time dependent on the used high voltage to achieve the best S/N ratio under the used parameters. After a baseline correction (acetonitrile as baseline), ECD and UV–vis spectra were expressed in terms of differential molar extinction (Δ*ε*) and molar extinction (*ε*), respectively (see Figures S22–S25A,B). The g_abs_ values were then obtained as Δ*ε*/*ε* from spectra of individual enantiomers and are plotted along with the CD. Emission spectra in excitation and emission modes were recorded in a quartz cuvette with a 10 mm optical path using a FP-8300 spectrofluorometer (JASCO, Japan) controlled by the Spectra Manager II software. Data were acquired at room temperature using the following measurement conditions: excitation and emission bandwidths of 5 nm, scanning speed of 100 nm/min, and a data interval of 0.5 nm. The emissions of 10^–5^ M solutions were measured using excitation wavelengths from the 330 to 370 nm interval (for details, see Table 1 in the Manuscript and see Figures S22–S25A). Absolute quantum yields (QYs) were determined using the same spectrofluorometer equipped with an ILF-835 integrating sphere accessory (100 mm diameter) for precise quantification. Measurements were performed at room temperature in a standard 10 mm quartz cuvette. Instrument calibration for QY determination was carried out using Jasco light sources (ESC-842, ESC-843). Data collection was conducted under a nitrogen atmosphere (4.8) with the following parameters: 1 mm aperture, excitation and emission spectral bandwidths of 5 nm, scanning speed of 100 nm/min, and a data interval of 0.5 nm. For all the measurements, the samples were prepared at 10^–4^ M concentrations in HPLC-grade acetonitrile. Prior to measurement, the solution was thoroughly degassed by bubbling acetonitrile-premoisturized argon (5.0) through it for at least 10 min, avoiding any concentration changes of the solution. The absolute QYs were measured using an excitation wavelength of 340 nm. Both the incident light and fluorescence intensities, under direct and indirect excitation, were recorded under the above-specified conditions. The QYs calculations were performed using Jasco’s Quantum Yield Calculation Program (FWQE-880), with corrections applied for indirect excitation. Chromaticity of the racemic solutions was obtained from emission spectra recorded as described above. To obtain and plot x, y coordinates, Chromaticity Diagram App and Origin2019b software by OriginLab were used, allowing free-format spectra processing. The results are reported in the CIE-1931 system with the inset of the zoomed blue region (see Figures S22–S25C). The circularly polarized luminescence (CPL) spectra were recorded in a screw cap-sealed glass cuvette with a 10 mm optical path with an Olis DSM172 spectrophotometer. For the measurements, an integration time of 4 s, a 10 nm emission bandpass, and a data interval of 1 nm were selected. The spectra were recorded in a 430–560 nm wavelength range at 10^–5^-10^–4^ M concentration range in degassed HPLC-grade acetonitrile at room temperature under a nitrogen (4.8) atmosphere. Degassing procedure details can be seen above. A fixed wavelength of 410 nm provided by an LED source was employed as the excitation source. The resulting g_lum_ values were obtained as an average from 100 scans. To obtain more precise g_lum_ values, the CPL spectra were smoothed using a shape-preserving Savitzky-Golay smoothing (polynomial order 4, window size 60 pts with reflection at the boundaries) to reduce the influence of noise. Such obtained values were used for *B*_CPL_ calculation. Raw data are plotted along with ΔI (see Figures S22D–S25D). The optical purity of each enantiomer was checked by using a Thermo Ultimate 3000 ProStar with a PDA detector (200–800 nm) using a Chiralpak IC (Chiral Technologies) column (250 × 4.6 mm, 10 μm) and *n-* heptane/dichloromethane/methyl *tert*-butyl ether 70:20:30 as a mobile phase at a flow rate of 1 mL/min. See Figures S26–S46. Diffraction data were collected on a Bruker D8 VENTURE Kappa Duo PHOTON III with the monochromated Mo/Cu–Kα radiation. The structures were solved by direct methods (SHELXT 2018/2)^32,33^ and refined by full-matrix least-squares on F^2^ values (SHELXL-2019/2).^34^ All heavy atoms were refined anisotropically. Hydrogen atoms were usually localized from the expected geometry and difference electron density maps. The hydrogen atoms were fixed into idealized positions (riding model) and assigned temperature factors Hiso­(H) = 1.2 Ueq­(pivot atom). ORTEP-3 ^35,36^ was used for structure presentation. See Figures S47–S50.

### Synthetic Procedures

#### General Procedure: Imidazole Formation

9,10-Diketo­[7]­helicene (30 mg, 1 eq, 0.0735 mmol), ammonium acetate (56 mg, 10 equiv), aniline (1.1 equiv), and benzaldehyde (1.1 equiv) were charged into a 10 mL round-bottom flask and suspended in acetic acid (3 mL). The reaction mixture was heated to 100 °C in an aluminum heating block until the full conversion was observed on TLC (usually around 2 h). Upon cooling, the mixture was poured into a saturated solution of NaHCO_3_ and extracted with ethyl acetate (3 × 20 mL). The combined organic phases were dried with MgSO_4_, evaporated, and purified with flash column chromatography, using ethyl acetate (EA)/ petroleum ether (PE) mixture.

##### 1,2-Diphenyl-1*H*-[7]­helicenoimidazole (**1-H**)

GP was followed with **DK­[7]** (30 mg, 1 eq, 0.0735 mmol), ammonium acetate (56 mg, 10 equiv), aniline (7.5 mg, 1.1 eq, 0.0809 mmol), benzaldehyde (8.6 mg, 1.1 eq, 0.0809 mmol), and acetic acid (3 mL). Product **1-H** was obtained after column chromatography on silica gel with EA/PE (10 → 30 vol %) as a yellow solid (33 mg, 79% yield). The same procedure was followed with (*P*)-**DK­[7]** (10 mg, 0.024 mmol) yielding 9 mg (0.016 mmol, 66%) of (*P*)-**1-H** and (*M*)-**DK­[7]** (10 mg, 0.024 mmol) yielding 8 mg (0.014 mmol, 57%) of (*M*)-**1-H**. ^1^H NMR (400 MHz, CDCl_3_) δ 9.04 (d, *J* = 8.2 Hz, 1H), 8.10 (d, *J* = 8.3 Hz, 1H), 7.82–7.77 (m, 1H), 7.76–7.65 (m, 5H), 7.64–7.55 (m, 3H), 7.50 (d, *J* = 7.8, 1.6 Hz, 1H), 7.41 (d, *J* = 8.5 Hz, 1H), 7.39–7.31 (m, 5H), 7.28 (d, *J* = 8.1, 1.3 Hz, 1H), 7.25 (d, *J* = 9.2 Hz, 1H), 7.12 (d, *J* = 8.5 Hz, 1H), 7.08 (d, *J* = 8.5 Hz, 1H), 6.93–6.86 (m, 2H), 6.43 (ddd, *J* = 8.5, 6.9, 1.4 Hz, 1H), 6.38 (ddd, *J* = 8.4, 6.9, 1.4 Hz, 1H). ^13^C {^1^H} NMR (101 MHz, CDCl_3_-*d*) δ 152.0, 138.8, 138.1, 131.7, 131.6, 130.6, 130.4, 130.22, 130.19, 129.9, 129.6, 129.55, 129.54 (2C), 129.4, 129.34, 129.33, 129.20, 129.16, 129.0, 128.4, 128.3 (2C), 127.0, 126.8, 126.5, 126.3 (2C), 126.2, 126.0, 125.3, 125.0, 124.9, 124.8, 124.7, 123.9, 123.6, 123.4, 122.8, 121.6, 121.1, 118.9. *R*_f_ = 0.43 (PE/EA 5:1). HRMS (APCI/QTOF) *m*/*z* [M + H]^+^ calculated for [C_43_H_27_N_2_]^+^ 571.2169; found 571.2166 (100%).

##### 1-Phenyl-2-(4-cyanophenyl)-1*H*-[7]­helicenoimidazole (**1-CN**)

GP was followed with **DK­[7]** (30 mg, 1 eq, 0.0735 mmol), ammonium acetate (56 mg, 10 equiv), aniline (7.5 mg, 1.1 eq, 0.0809 mmol), 4-cyanobenzaldehyde (10.6 mg, 1.1 eq, 0.0809 mmol), and acetic acid (3 mL). Product **1-CN** was obtained after column chromatography on silicagel with EA/PE (10 → 30 vol %) as a yellow solid (39 mg, 90% yield). The same procedure was followed with (*P*)-**DK­[7]** (10 mg, 0.024 mmol) yielding 11 mg (0.016 mmol, 75%) of (*P*)-**1-CN** and (*M*)-**DK­[7]** (10 mg, 0.024 mmol) yielding 11 mg (0.016 mmol, 75%) of (*M*)-**1-CN**. ^1^H NMR (400 MHz, CDCl_3_) δ 8.98 (d, *J* = 8.2 Hz, 1H), 8.11 (d, *J* = 8.3 Hz, 1H), 7.84–7.71 (m, 6H), 7.70–7.60 (m, 4H), 7.57 (d, *J* = 8.5 Hz, 1H), 7.51 (d, *J* = 7.8 Hz, 1H), 7.42 (d, *J* = 8.5 Hz, 1H), 7.38 (d, *J* = 8.5 Hz, 1H), 7.32 (d, *J* = 8.5 Hz, 1H), 7.28 (d, *J* = 8.1 Hz, 1H), 7.25 (d, *J* = 8.9 Hz, 1H), 7.09 (d, *J* = 8.5 Hz, 1H), 7.05 (d, *J* = 8.5 Hz, 1H), 6.95–6.86 (m, 2H), 6.46–6.34 (m, 2H). ^13^C {^1^H} NMR (101 MHz, CDCl_3_) δ 149.3, 138.43, 138.37, 134.9, 132.0 (2C), 131.69, 131.65, 130.64, 130.62, 130.60, 130.5, 130.0, 129.61 (3C), 129.57, 129.56, 129.3, 129.2, 129.1, 129.0, 128.7, 127.2, 127.1, 126.6, 126.5, 126.4, 126.1, 125.9, 125.2, 125.1, 125.0, 124.8, 124.7, 124.5, 123.7, 123.5, 123.1, 121.4, 120.9, 118.8, 118.5, 112.2. *R*_f_ = 0.35 (PE/EA 5:1). HRMS (APCI/QTOF) *m*/*z* [M + H]^+^ calculated for [C_44_H_26_N_3_]^+^ 596.2121; found 596.2117 (100%).

##### 1-Phenyl-2-(4-(trifluoromethyl)­phenyl)-1*H*-[7]­helicenoimidazole (**1-CF_3_**)

GP was followed with **DK­[7]** (30 mg, 1 eq, 0.0735 mmol), ammonium acetate (56 mg, 10 equiv), aniline (7.5 mg, 1.1 eq, 0.0809 mmol), 4-trifluoromethylbenzaldehyde (14.1 mg, 1.1 eq, 0.0809 mmol), and acetic acid (3 mL). Product **1-CF**_**3**_ was obtained after column chromatography on silicagel with EA/PE (10 → 30 vol %) as a yellow solid (42 mg, 89% yield). ^1^H NMR (400 MHz, CDCl_3_) δ 9.01 (d, *J* = 8.2 Hz, 1H), 8.11 (d, *J* = 8.2 Hz, 1H), 7.84–7.71 (m, 6H), 7.67–7.55 (m, 5H), 7.52 (d, *J* = 7.9 Hz, 1H), 7.42 (d, *J* = 8.5 Hz, 1H), 7.38 (d, *J* = 8.5 Hz, 1H), 7.34 (d, *J* = 8.5 Hz, 1H), 7.28 (d, *J* = 7.9 Hz, 1H), 7.25 (d, *J* = 8.3 Hz, 1H), 7.11 (d, *J* = 8.5 Hz, 1H), 7.07 (d, *J* = 8.5 Hz, 1H), 6.95–6.85 (m, 2H), 6.46–6.36 (m, 2H). ^13^C {^1^H} NMR (101 MHz, CDCl_3_) δ 150.1, 138.5, 138.3, 134.1, 131.69, 131.65, 130.52 (2C), 130.51, 130.3, 129.8, 129.59, 129.56 (3C), 129.51, 129.4, 129.19, 129.18, 129.1, 128.6, 127.1, 127.0, 126.5 (2C), 126.4, 126.2, 126.0, 125.30, 125.26, 125.23, 125.19, 125.0 (2C), 124.7 (2C), 124.2, 123.7, 123.5, 123.0, 122.6, 121.5, 121.0, 118.8. ^19^F NMR (376 MHz, CDCl_3_) δ −62.76. *R*_f_ = 0.61 (PE/EA 5:1). HRMS (APCI/QTOF) *m*/*z* [M + H]^+^ calculated for [C_44_H_26_N_2_F_3_]^+^ 639.2043; found 639.2041 (100%).

##### 1-(4-(*N*,*N*-Diphenylamino)­phenyl)-2-phenyl-1*H*-[7]­helicenoimidazole (**2-H**)

GP was followed with **DK­[7]** (30 mg, 1 eq, 0.0735 mmol), ammonium acetate (56 mg, 10 equiv), *N* ^1^,*N* ^1^-diphenylbenzene-1,4-diamine (21 mg, 1.1 eq, 0.0809 mmol), benzaldehyde (8.6 mg, 1.1 eq, 0.0809 mmol), and acetic acid (3 mL). Product **2-H** was obtained after column chromatography on silicagel with EA/PE (10 → 20 vol %) as a yellow solid (40 mg, 74% yield). The same procedure was followed with (*P*)-**DK­[7]** (10 mg, 0.024 mmol) yielding 12 mg (0.016 mmol, 66%) of (*P*)-**2-H** and (*M*)-**DK­[7]** (10 mg, 0.024 mmol) yielding 13 mg (0.018 mmol, 72%) of (*M*)-**2-H**. ^1^H NMR (400 MHz, CDCl_3_) δ 9.02 (d, *J* = 8.3 Hz, 1H), 8.09 (d, *J* = 8.3 Hz, 1H), 7.82–7.70 (m, 4H), 7.69–7.61 (m, 2H), 7.58 (dd, *J* = 8.6, 2.6 Hz, 1H), 7.46–7.23 (m, 17H), 7.20 (dd, *J* = 8.6, 2.7 Hz, 1H), 7.18–7.06 (m, 4H), 6.95–6.86 (m, 2H), 6.41 (dddd, *J* = 15.7, 8.4, 6.9, 1.4 Hz, 2H). ^13^C {^1^H} NMR (101 MHz, CDCl_3_) δ 152.2, 149.1, 147.1, 138.1, 131.7, 131.64, 131.59, 130.8, 130.4, 129.9, 129.8, 129.7 (4C), 129.60 (2C), 129.55, 129.4, 129.3, 129.2, 129.0, 128.4, 128.3 (2C), 127.0, 126.8, 126.5, 126.33, 126.31, 126.29, 126.0, 125.3, 125.2 (4C), 125.1, 124.9, 124.8, 124.6, 124.1 (2C), 123.8, 123.6, 123.4, 122.9 (2C), 122.7, 121.8, 121.1, 119.0. *R*_f_ = 0.38 (PE/EA 5:1). HRMS (APCI/QTOF) *m*/*z* [M + H]^+^ calculated for [C_55_H_36_N_3_]^+^ 738.2904; found 738.2902 (100%).

##### 1-(4-(*N*,*N*-Diphenylamino)­phenyl)-2-(4-cyanophenyl)-1*H*-[7]­helicenoimidazole (**2-CN**)

GP was followed with **DK­[7]** (30 mg, 1 eq, 0.0735 mmol), ammonium acetate (56 mg, 10 equiv), *N* ^1^,*N* ^1^-diphenylbenzene-1,4-diamine (21 mg, 1.1 eq, 0.0809 mmol), 4-cyanobenzaldehyde (10.6 mg, 1.1 eq, 0.0809 mmol), and acetic acid (3 mL). Product **2-CN** was obtained after column chromatography on silica gel with EA/PE (10 → 20 vol %) as a yellow solid (42 mg, 75% yield). The same procedure was followed with (*P*)-**DK­[7]** (10 mg, 0.024 mmol) yielding 13 mg (0.017 mmol, 70%) of (*P*)-**2-CN** and (*M*)-**DK­[7]** (10 mg, 0.024 mmol) yielding 14 mg (0.018 mmol, 75%) of (*M*)-**2-CN**. ^1^H NMR (400 MHz, CDCl_3_) δ 8.97 (d, *J* = 8.2 Hz, 1H), 8.10 (d, *J* = 8.2 Hz, 1H), 7.92 (d, *J* = 8.2 Hz, 2H), 7.81–7.67 (m, 4H), 7.63 (d, *J* = 8.3 Hz, 2H), 7.56 (dd, *J* = 8.6, 2.5 Hz, 1H), 7.46–7.38 (m, 6H), 7.36 (d, *J* = 2.7 Hz, 1H), 7.33–7.15 (m, 10H), 7.08 (dd, *J* = 13.1, 8.5 Hz, 2H), 6.91 (t, *J* = 7.4 Hz, 2H), 6.41 (ddd, *J* = 15.3, 8.7, 6.8 Hz, 2H). ^13^C {^1^H} NMR (101 MHz, CDCl_3_) δ 149.6, 146.8, 138.3, 135.1, 132.0 (2C), 131.70, 131.66, 130.58, 130.56, 130.2, 129.8 (4C), 129.7 (2C), 129.63, 129.59, 129.5, 129.4, 129.2, 128.6, 127.2, 127.1, 126.5 (2C), 126.4, 126.1, 126.0, 125.6 (4C), 125.2, 125.1, 125.0, 124.8, 124.7, 124.5 (2C), 124.4, 123.7, 123.5, 123.1, 122.4 (2C), 121.6, 120.9, 118.9, 118.7, 112.2. *R*_f_ = 0.43 (PE/EA 5:1). HRMS (ESI/QTOF) *m*/*z* [M + H]^+^ calculated for [C_56_H_35_N_4_]^+^ 763.2856; found 763.2853 (100%).

##### 1-(4-(*N*,*N*-Diphenylamino)­phenyl)-2-(4-(trifluoromethyl)­phenyl)-1*H*-[7]­helicenoimidazole (**2-CF_3_**)

GP was followed with **DK­[7]** (30 mg, 1 eq, 0.0735 mmol), ammonium acetate (56 mg, 10 equiv), *N* ^1^,*N* ^1^-diphenylbenzene-1,4-diamine (21 mg, 1.1 eq, 0.0809 mmol), 4-(trifluoromethyl)­benzaldehyde (14 mg, 1.1 eq, 0.0809 mmol), and acetic acid (3 mL). Product **2-CF**_**3**_ was obtained after column chromatography on silica gel with EA/PE (0 → 10 vol %) as a yellow solid (35 mg, 59% yield). ^1^H NMR (400 MHz, CD_2_Cl_2_) δ 8.95 (d, *J* = 8.2 Hz, 1H), 8.12 (d, *J* = 8.3 Hz, 1H), 7.96 (d, *J* = 8.1 Hz, 2H), 7.83–7.74 (m, 2H), 7.74–7.65 (m, 4H), 7.60 (dd, *J* = 8.6, 2.6 Hz, 1H), 7.46–7.14 (m, 17H), 7.07 (t, *J* = 9.4 Hz, 2H), 6.91 (t, *J* = 7.4 Hz, 2H), 6.45–6.36 (m, 2H). ^13^C {^1^H} NMR (101 MHz, CDCl_3_) δ 150.4, 149.4, 146.9, 138.2, 134.3, 131.69, 131.66, 130.9, 130.5, 130.4, 129.9, 129.7, 129.64, 129.62, 129.61, 129.5, 129.4, 129.2, 128.6, 127.1, 127.0, 126.5, 126.44, 126.38, 126.2, 126.0, 125.5, 125.3, 125.23, 125.20, 125.16, 125.0, 124.8, 124.7, 124.4, 124.2, 123.7, 123.5, 122.9, 122.6, 121.6, 121.0, 119.0, 29.70. ^19^F {^1^H} NMR (376 MHz, CDCl_3_) δ −62.67. *R*_f_ = 0.57 (PE/EA 10:1). HRMS (APCI/QTOF) *m*/*z* [M + H]^+^ calculated for [C_56_H_35_F_3_N_3_]^+^ 806.2778; found 806.2779 (100%).

##### 1-(9*H*-Carbazol-9-yl)-phenyl-4-ene-2-phenyl-1*H*-[7]­helicenoimidazole (**3-H**)

GP was followed with **DK­[7]** (30 mg, 1 eq, 0.0734 mmol), ammonium acetate (56.6 mg, 10 eq, 0.7345), 4-(9H-carbazol-9-yl)­aniline (20.9 mg, 1.1 eq, 0.0808 mmol), benzaldehyde (8.6 mg, 1.1 eq, 0.0808 mmol), and acetic acid (3 mL). Product **3-H** was obtained after column chromatography on silica gel with EA/PE (10 → 30 vol %) as a yellow solid (39 mg, 72% yield). The same procedure was followed with (*M*)- **DK­[7]** (9 mg, 0.0220 mmol) and 7 mg (43% yield) of (*M*)-**3-H** was obtained. The same procedure was followed with (*P*)-**DK­[7]** (9 mg, 0.0220 mmol) and 13 mg (80% yield) of (*P*)-**3-H** was obtained. ^1^H NMR (400 MHz, CDCl_3_) δ 9.07 (d, *J* = 9.2 Hz, 1H), 8.23 (d, *J* = 7.8 Hz, 2H), 8.13 (d, *J* = 8.3 Hz, 1H), 8.01 (dd, *J* = 8.3, 2.4 Hz, 1H), 7.93 (dd, *J* = 8.3, 2.3 Hz, 1H), 7.84–7.68 (m, 6H), 7.67–7.51 (m, 6H), 7.50–7.36 (m, 7H), 7.33–7.26 (m, 2H), 7.15 (dd, *J* = 13.6, 8.5 Hz, 2H), 6.92 (tdd, *J* = 6.7, 2.6, 1.1 Hz, 2H), 6.50–6.36 (m, 2H). ^13^C {^1^H} NMR (101 MHz, CDCl_3_) δ 152.1, 140.4, 139.1, 138.3, 137.3, 131.7, 130.9, 130.8, 130.5, 129.7, 129.6 (2C), 129.4, 129.24, 129.16, 128.6, 128.44 (2C), 128.38, 128.35, 127.2, 127.0, 126.5, 126.44, 126.38, 126.32 (2C), 126.25, 126.0, 125.3, 125.0, 124.8, 124.7, 124.0, 123.8, 123.7, 123.5, 122.9, 121.5, 121.1, 120.7, 120.6, 118.7, 109.5 (2C). *R*_f_ = 0.29 (PE/EA 8:1). HRMS (APCI/QTOF) *m*/*z* [M + H]^+^ calculated for [C_55_H_34_N_3_]^+^ 736.2747; found 736.2745 (100%).

##### 1-(9*H*-Carbazol-9-yl)-phenyl-4-ene-2-(4-cyanophenyl)-1*H*-[7]­helicenoimidazole (**3-CN**)

GP was followed with **DK­[7]** (30 mg, 1 eq, 0.0734 mmol), ammonium acetate (56.6 mg, 10 eq, 0.7345), 4-(9H-carbazol-9-yl)­aniline (20.9 mg, 1.1 eq, 0.0808 mmol), 4-cyanobenzaldehyde (10. 6 mg, 1.1 eq, 0.0808 mmol), and acetic acid (3 mL). Product **3-CN** was obtained after column chromatography on silica gel with EA/PE (10 → 30 vol %) as a yellow solid (47 mg, 84% yield). The same procedure was followed with (*M*)-**DK­[7]** (9 mg, 0.0220 mmol) and 11 mg (66% yield) of (*M*)-**3-CN** was obtained. The same procedure was followed with (*P*)-**DK**[7] (9 mg, 0.0220 mmol) and 12 mg (72% yield) of (*P*)-**3-CN** was obtained. ^1^H NMR (400 MHz, CDCl_3_) δ 9.01 (d, *J* = 8.2 Hz, 1H), 8.24 (d, *J* = 7.8 Hz, 2H), 8.14 (d, *J* = 8.3 Hz, 1H), 8.10–7.99 (m, 2H), 7.98–7.85 (m, 3H), 7.81–7.69 (m, 5H), 7.66–7.51 (m, 6H), 7.46–7.39 (m, 4H), 7.31–7.27 (m, 2H), 7.11 (d, *J* = 8.4 Hz, 1H), 7.08 (d, *J* = 8.5 Hz, 1H), 6.92 (dddd, *J* = 8.0, 6.9, 2.8, 1.2 Hz, 2H), 6.43 (dddd, *J* = 17.0, 8.4, 6.8, 1.4 Hz, 2H). ^13^C {^1^H} NMR (101 MHz, CDCl_3_) δ 149.5, 140.3, 140.0, 138.6, 136.8, 135.7, 134.8, 132.2, 131.74, 131.72, 130.8, 130.70, 130.67, 130.1, 129.8, 129.73, 129.71, 129.6, 129.3, 129.2, 128.8, 128.70, 128.69, 127.4, 127.3, 126.7, 126.6, 126.5, 126.0, 125.9, 125.21, 125.18, 125.0, 124.9, 124.7, 124.0, 123.8, 123.6, 123.2, 121.2, 120.9, 120.8, 118.6, 118.4, 112.6, 109.4. *R*_f_ = 0.16 (PE/EA 8:1). HRMS (APCI/QTOF) *m*/*z* [M + H]^+^ calculated for [C_56_H_33_N_4_]^+^ 761.2700; found 761.2702 (100%).

##### 1-(9*H*-carbazol-9-yl)-phenyl-4-ene-2-(4-(trifluoromethyl)­phenyl)-1*H*-[7]­helicenoimidazole (**3-CF_3_**)

GP was followed with **DK­[7]** (30 mg, 1 eq, 0.0734 mmol), ammonium acetate (56.6 mg, 10 eq, 0.7345), 4-(9H-carbazol-9-yl)­aniline (20.9 mg, 1.1 eq, 0.0808 mmol), 4-(trifluoromethyl)­benzaldehyde (14.1 mg, 1.1 eq, 0.0808 mmol) and acetic acid (3 mL). Product **3-CF**_**3**_ was obtained after column chromatography on silica gel with EA/PE (10 → 30 vol %) as a yellow solid (44 mg, 75% yield). ^1^H NMR (400 MHz, CDCl_3_) δ 9.03 (d, *J* = 8.2 Hz, 1H), 8.23 (d, *J* = 7.8 Hz, 2H), 8.14 (d, *J* = 8.2 Hz, 1H), 8.08–7.97 (m, 2H), 7.92 (d, *J* = 8.1 Hz, 2H), 7.87 (dd, *J* = 8.3, 2.3 Hz, 1H), 7.80–7.73 (m, 3H), 7.70 (d, *J* = 8.2 Hz, 2H), 7.65–7.53 (m, 6H), 7.46–7.37 (m, 4H), 7.32–7.26 (m, 2H), 7.18–7.06 (m, 2H), 6.98–6.86 (m, 2H), 6.43 (dddd, *J* = 16.8, 8.4, 6.9, 1.4 Hz, 2H). ^13^C {^1^H} NMR (101 MHz, CDCl_3_) δ 150.2, 140.3, 139.5, 138.4, 136.9, 134.0, 131.7 (q, *J*_*CF*_ = 1 Hz), 130.84, 130.78 (q, *J*_*CF*_ = 33 Hz), 130.7, 130.6, 129.8, 129.7, 129.61, 129.58, 129.4, 129.2, 128.7, 128.58, 128.55, 127.4, 127.2, 126.61, 126.56, 126.43, 126.41, 126.1, 126.0, 125.4 (q, *J*_*CF*_ = 3.7 Hz), 125.2, 125.1, 125.0, 124.8, 124.7, 124.4, 123.92, 123.91 (q, *J*_*CF*_ = 272.5 Hz), 123.8, 123.5, 123.1, 121.3, 121.0, 120.8, 120.7, 118.6, 109.5. ^19^F NMR (376 MHz, CDCl_3_) δ −62.73. *R*_f_ = 0.51 (PE/EA 8:1). HRMS (APCI/QTOF) *m*/*z* [M + H]^+^ calculated for [C_56_H_33_F_3_N_3_]^+^ 804.2621; found 804.2619 (100%).

## Supplementary Material





## Data Availability

The data underlying this study are available in the published article and its Supporting Information.

## Notes

^1^H, ^19^F, and ^13^C­{^1^H} NMR spectra, HPLC spectra for both racemic and enantiomerically pure materials, absorption and luminescence spectra, crystallographic data, and DFT calculations (PDF)
FAIR data, including the primary NMR FID files, for compounds **1-H**–**3-H**, **1-CN**–**3-CN**, and **1-CF**_**3**_–**3-CF**_**3**_ (ZIP)
