# Supplementary figures and images for: Tuning Photophysical and Chiroptical Properties of [7]Helicene through Formation of Imidazole-Based Push–Pull Systems
Source: J Org Chem. 2026 May 19;91(22):7391–9. doi: 10.1021/acs.joc.6c00092 (PMC13247978; doi:10.1021/acs.joc.6c00092)

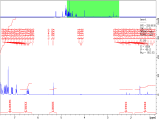

Supplement: Supplementary file 1 [file jo6c00092_si_001.zip › Imidazole_NMR/1-CF3/1H/pdata/1/thumb.png]

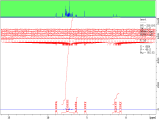

Supplement: Supplementary file 1 [file jo6c00092_si_001.zip › Imidazole_NMR/1-CN/1H/pdata/1/thumb.png]
