# Supplementary material for: Tuning Photophysical and Chiroptical Properties of [7]Helicene through Formation of Imidazole-Based Push–Pull Systems
Source: J Org Chem. 2026 May 19;91(22):7391–9. doi: 10.1021/acs.joc.6c00092 (PMC13247978; doi:10.1021/acs.joc.6c00092)
Supplement: Supplementary file 2 [file jo6c00092_si_002.pdf]

Supporting Information

Tuning Photophysical and Chiroptical Properties of [7]helicene through Formation of Imidazole-based Push-Pull Systems

Martin Kos[1,†], Tomáš Beránek[1,†], Sachika Takase[1], Jaroslav Žádný[1], Jan Sýkora[2], Ivana Císařová[3], Jan Storch[1], Vladimír Církva[1], Martin Jakubec[1,*]

[1]Department of Materials Chemistry, Research Group of Advanced Materials and Organic Synthesis, Institute of Chemical Process Fundamentals of the Czech Academy of Sciences, v. v. i., Rozvojová 135, 165 00 Prague 6, Czech Republic
[2]Department of Analytical Chemistry, University of Chemistry and Technology Prague, 166 28 Prague 6, Czech Republic
[3]Department of Inorganic Chemistry, Faculty of Science, Charles University in Prague, Hlavova 2030, 128 40 Prague 2, Czech Republic

† The authors contributed equally.

Corresponding author: *) Martin Jakubec (jakubecm@icpf.cas.cz)

Content

1. General Information 3
   - 1.1 Materials and Methods 3
   - 1.2 NMR spectrometry 3
   - 1.3 Mass spectrometry 3
   - 1.4 Absorption 3
   - 1.5 Luminescence 3
   - 1.6 Chiral-HPLC Analysis 4
   - 1.7 Crystallographic data 4
2. Synthetic Procedures 5
3. NMR Spectra 10
4. **DFT Calculation Details of 1-3** 21
   - **4.1** Cartesian coordinates of optimized structures of **1-H – 3-H, 1-CN – 3-CN and 1-CF_3 – 3-CF_3** .21
   - **1-H** 21
   - **1-CN** 23
   - **1-CF_3** 26
   - **2-H** 29
   - **2-CN** 32
   - **2-CF_3** 35
   - **3-H** 38
   - **3-CN** 42
   - **3-CF_3** 45
   - 7hel 48
5. Frontier MOs of compounds 1-3 51
6. Absorption and Luminiscence spectra 54
7. Summary of calculated and experimental properties 58
8. Chiral-HPLC Analysis 59
9. Crystallographic data 70
10. References 72

1. General Information

1.1 Materials and Methods

Commercially available reagent grade materials were used as obtained from Sigma-Aldrich, Acros Organics, Apollo Scientific, and Fluorochem. All solvents were of a reagent grade and used without any further purification, except for tetrahydrofuran and toluene, which were freshly distilled from sodium/benzophenone, and dichloromethane, which was freshly distilled from calcium hydride. TLC was carried out using silica gel 60 F254-coated aluminum sheets, and compounds were visualized with UV light (254 and 366 nm). Column chromatography was performed using Biotage HPFC systems (Isolera One) with prepacked flash silica gel columns.

1.2 NMR spectrometry

^{1}H and $^{13}C\{^{1}H\}$ NMR spectra were recorded using Bruker Avance spectrometer at 400 MHz (^{1}H NMR), 101 MHz (^{13}C NMR), and 376 MHz (^{19}F NMR). Chemical shifts (δ) are reported in parts per million (ppm) and referenced to residuals of $CDCl_3$ (δ = 7.26 and 77.00 ppm, respectively) or CD_2Cl_2 (δ = 5.30 and 54.00 ppm, respectively). The coupling constants (*J*) are given in hertz (Hz) and the corresponding multiplicity (s = singlet, d = doublet, t = triplet, m = multiplet).

1.3 Mass spectrometry

For exact mass measurement, the spectra were internally calibrated using Na-formate or APCI-TOF tuning mix. APCI high-resolution mass spectra were measured in a positive mode using a micrOTOF QIII mass spectrometer (Bruker) and determined by software Compass Data Analysis.

1.4 Absorption

The ECD and absorption spectra were recorded on an Olis DSM172 spectrophotometer. The ECD and absorption spectra were recorded over a spectral range of 190 nm to 650 nm in acetonitrile (10^{-5}-10^{-4} M). Absorption was recorded in a quartz cuvette with a 10 mm optical path at constant DIT of 0.1 sec, a bandpass width of 1 nm, and a data interval of 0.5 nm. ECD measurements were made in a quartz cell with a 2 mm path length using a fixed bandpass width of 1 nm and variable integration time dependent on the used high voltage to achieve the best S/N ratio under the used parameters. After a baseline correction (acetonitrile as baseline), ECD and UV-Vis spectra were expressed in terms of differential molar extinction ($\Delta\varepsilon$) and molar extinction (ε), respectively (see Figs. S22-S25A and B). The g_{abs} values were then obtained as $\Delta\varepsilon/\varepsilon$ from spectra of individual enantiomers and are plotted along with the CD.

1.5 Luminescence

Emission spectra in excitation and emission modes were recorded in a quartz cuvette with a 10 mm optical path using a FP-8300 spectrofluorometer (JASCO, Japan) controlled by the Spectra Manager II software. Data were acquired at room temperature using the following measurement conditions: excitation and emission bandwidths of 5 nm, scanning speed of 100 nm/min, and a data interval of 0.5 nm. The emissions of 10^{-5} M solutions were measured using excitation wavelengths from the 330 to 370 nm interval (for details, see Table 1 in the Manuscript and see Figs. S22-S25A).

Absolute quantum yields (QYs) were determined using the same spectrofluorometer equipped with an ILF-835 integrating sphere accessory (100 mm diameter) for precise quantification. Measurements were performed at room temperature in a standard 10 mm quartz cuvette. Instrument calibration for QY determination was carried out using Jasco light sources (ESC-842, ESC-843). Data collection was

conducted under a nitrogen atmosphere (4.8) with the following parameters: 1 mm aperture, excitation and emission spectral bandwidths of 5 nm, scanning speed of 100 nm/min, and a data interval of 0.5 nm. For all the measurements, the samples were prepared at 10^{-4} M concentrations in HPLC-grade acetonitrile. Prior to measurement, the solution was thoroughly degassed by bubbling acetonitrile-pre-moisturized argon (5.0) through it for at least 10 minutes, avoiding any concentration changes of the solution. The absolute QYs were measured using an excitation wavelength of 340 nm. Both the incident light and fluorescence intensities, under direct and indirect excitation, were recorded under the above-specified conditions. The QYs calculations were performed using Jasco's Quantum Yield Calculation Program (FWQE-880), with corrections applied for indirect excitation.

Chromaticity of the racemic solutions was obtained from emission spectra recorded as described above. To obtain and plot x, y coordinates, Chromaticity Diagram App and Origin2019b software by OriginLab were used, allowing free-format spectra processing. The results are reported in the CIE-1931 system with the inset of the zoomed blue region (see Figs. S22-S25C).

The circularly polarized luminescence (CPL) spectra were recorded in a screw cap-sealed glass cuvette with a 10 mm optical path with an Olis DSM172 spectrophotometer. For the measurements, an integration time of 4 s, a 10 nm emission bandpass, and a data interval of 1 nm were selected. The spectra were recorded in a 430-560 nm wavelength range at 10^{-5}-10^{-4} M concentration range in degassed HPLC-grade acetonitrile at room temperature under a nitrogen (4.8) atmosphere. Degassing procedure details can be seen above. A fixed wavelength of 410 nm provided by an LED source was employed as the excitation source. The resulting g_{lum} values were obtained as an average from 100 scans. To obtain more precise g_{lum} values, the CPL spectra were smoothed using a shape-preserving Savitzky-Golay smoothing (polynomial order 4, window size 60 pts with reflection at the boundaries) to reduce the influence of noise. Such obtained values were used for B_{CPL} calculation. Raw data are plotted along with ΔI (see Figs. S22D-S25D).

1.6 Chiral-HPLC Analysis

The optical purity of each enantiomer was checked by using a Thermo Ultimate 3000 ProStar with a PDA detector (200−800 nm) using a Chiralpak® IC™ (Chiral Technologies) column (250 × 4.6 mm, 10 µm) and *n*-heptane/dichloromethane/methyl *tert*-butyl ether 70:20:30 as a mobile phase at a flow rate of 1 mL/min. See Figs. S26-S46.

1.7 Crystallographic data

Diffraction data were collected on a Bruker D8 VENTURE Kappa Duo PHOTON III with the monochromated Mo/Cu-Kα radiation. The structures were solved by direct methods (SHELXT 2018/2)[1] and refined by full-matrix least-squares on F^2 values (SHELXL-2019/2)[2]. All heavy atoms were refined anisotropically. Hydrogen atoms were usually localized from the expected geometry and difference electron density maps. The hydrogen atoms were fixed into idealized positions (riding model) and assigned temperature factors Hiso(H) = 1.2 Ueq(pivot atom). ORTEP-3[3,4] was used for structure presentation.

The crystallographic data for the structures reported in this paper have been deposited with the Cambridge Crystallographic Data Centre as supplementary publication. Copies of the data can be obtained free of charge on application to CCDC, e-mail: deposit@ccdc.cam.ac.uk. See Figs. S47-S50.

2. Synthetic Procedures

General procedure – Imidazole formation

9,10-Diketo[7]helicene (30 mg, 1 eq, 0.0735 mmol), ammonium acetate (56 mg, 10 eq), aniline (1.1 eq), and benzaldehyde (1.1 eq) were charged into a 10 mL round-bottom flask and suspended in acetic acid (3 mL). The reaction mixture was heated to 100°C in an aluminum heating block until the full conversion was observed on TLC (usually around 2 hours). Upon cooling, the mixture was poured into a saturated solution of $NaHCO_3$ and extracted with ethyl acetate (3 x 20 mL). The combined organic phases were dried with $MgSO_4$, evaporated, and purified with flash column chromatography, using ethyl acetate (EA)/ petroleum ether (PE) mixture.

1,2-Diphenyl-1*H*-[7]helicenoimidazole (1-H)

GP was followed with **DK[7]** (30 mg, 1 eq, 0.0735 mmol), ammonium acetate (56 mg, 10 eq), aniline (7.5 mg, 1.1 eq, 0.0809 mmol), benzaldehyde (8.6 mg, 1.1 eq, 0.0809 mmol), and acetic acid (3 mL). Product **1-H** was obtained after column chromatography on silica gel with EA/PE (10 -> 30 vol%) as a yellow solid (33 mg, 79% yield).

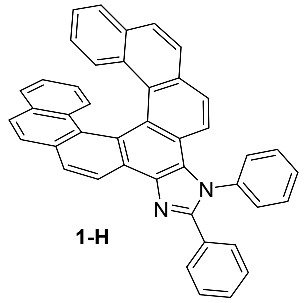

1-H

The same procedure was followed with (*P*)-**DK[7]** (10 mg, 0.024 mmol) yielding 9 mg (0.016 mmol, 66 %) of (*P*)-**1-H** and (*M*)-**DK[7]** (10 mg, 0.024 mmol) yielding 8 mg (0.014 mmol, 57%) of (*M*)-**1-H**.

^{1}H NMR (400 MHz, $CDCl_3$) δ 9.04 (d, *J* = 8.2 Hz, 1H), 8.10 (d, *J* = 8.3 Hz, 1H), 7.82 – 7.77 (m, 1H), 7.76 – 7.65 (m, 5H), 7.64 – 7.55 (m, 3H), 7.50 (d, *J* = 7.8, 1.6 Hz, 1H), 7.41 (d, *J* = 8.5 Hz, 1H), 7.39 – 7.31 (m, 5H), 7.28 (d, *J* = 8.1, 1.3 Hz, 1H), 7.25 (d, *J* = 9.2 Hz, 1H), 7.12 (d, *J* = 8.5 Hz, 1H), 7.08 (d, *J* = 8.5 Hz, 1H), 6.93 – 6.86 (m, 2H), 6.43 (ddd, *J* = 8.5, 6.9, 1.4 Hz, 1H), 6.38 (ddd, *J* = 8.4, 6.9, 1.4 Hz, 1H).

^{13}C {^{1}H} NMR (101 MHz, $CDCl_3$-*d*) δ 152.0, 138.8, 138.1, 131.7, 131.6, 130.6, 130.4, 130.22, 130.19, 129.9, 129.6, 129.55, 129.54 (2C), 129.4, 129.34, 129.33, 129.20, 129.16, 129.0, 128.4, 128.3 (2C), 127.0, 126.8, 126.5, 126.3 (2C), 126.2, 126.0, 125.3, 125.0, 124.9, 124.8, 124.7, 123.9, 123.6, 123.4, 122.8, 121.6, 121.1, 118.9.

R_f = 0.43 (PE/EA 5:1)

HRMS (APCI/QTOF) *m/z* $[M + H]^+$ calculated for $[C_{43}H_{27}N_2]^+$ 571.2169 ; found 571.2166 (100%).

1-Phenyl-2-(4-cyanophenyl)-1*H*-[7]helicenoimidazole (1-CN)

GP was followed with **DK[7]** (30 mg, 1 eq, 0.0735 mmol), ammonium acetate (56 mg, 10 eq), aniline (7.5 mg, 1.1 eq, 0.0809 mmol), 4-cyanobenzaldehyde (10.6 mg, 1.1 eq, 0.0809 mmol), and acetic acid (3 ml). Product **1-CN** was obtained after column chromatography on silicagel with EA/PE (10 -> 30 vol%) as a yellow solid (39 mg, 90% yield).

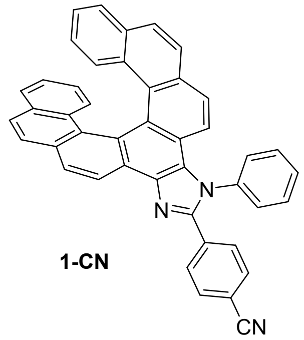

1-CN

The same procedure was followed with (*P*)-**DK[7]** (10 mg, 0.024 mmol) yielding 11 mg (0.016 mmol, 75 %) of (*P*)-**1-CN** and (*M*)-**DK[7]** (10 mg, 0.024 mmol) yielding 11 mg (0.016 mmol, 75%) of (*M*)-**1-CN**.

^{1}H NMR (400 MHz, $CDCl_3$) δ 8.98 (d, *J* = 8.2 Hz, 1H), 8.11 (d, *J* = 8.3 Hz, 1H), 7.84 – 7.71 (m, 6H), 7.70 – 7.60 (m, 4H), 7.57 (d, *J* = 8.5 Hz, 1H), 7.51 (d, *J* = 7.8 Hz, 1H), 7.42 (d, *J* = 8.5 Hz, 1H), 7.38 (d, *J* = 8.5 Hz, 1H), 7.32 (d, *J* = 8.5 Hz, 1H), 7.28 (d, *J* = 8.1 Hz, 1H), 7.25 (d, *J* = 8.9 Hz, 1H), 7.09 (d, *J* = 8.5 Hz, 1H), 7.05 (d, *J* = 8.5 Hz, 1H), 6.95 – 6.86 (m, 2H), 6.46 – 6.34 (m, 2H).

^{13}C {^{1}H} NMR (101 MHz, $CDCl_3$) δ 149.3, 138.43, 138.37, 134.9, 132.0 (2C), 131.69, 131.65, 130.64, 130.62, 130.60, 130.5, 130.0, 129.61 (3C), 129.57, 129.56, 129.3, 129.2, 129.1, 129.0, 128.7, 127.2, 127.1, 126.6, 126.5, 126.4, 126.1, 125.9, 125.2, 125.1, 125.0, 124.8, 124.7, 124.5, 123.7, 123.5, 123.1, 121.4, 120.9, 118.8, 118.5, 112.2.

R_f = 0.35 (PE/EA 5:1)

HRMS (APCI/QTOF) *m/z* $[M + H]^+$ calculated for $[C_{44}H_{26}N_3]^+$ 596.2121; found 596.2117 (100%).

1-Phenyl-2-(4-(trifluoromethyl)phenyl)-1*H*-[7]helicenoimidazole (1-CF₃)

GP was followed with **DK[7]** (30 mg, 1 eq, 0.0735 mmol), ammonium acetate (56 mg, 10 eq), aniline (7.5 mg, 1.1 eq, 0.0809 mmol), 4-trifluoromethylbenzaldehyde (14.1 mg, 1.1 eq, 0.0809 mmol), and acetic acid (3 ml). Product **1-CF₃** was obtained after column chromatography on silicagel with EA/PE (10 -> 30 vol%) as a yellow solid (42 mg, 89% yield).

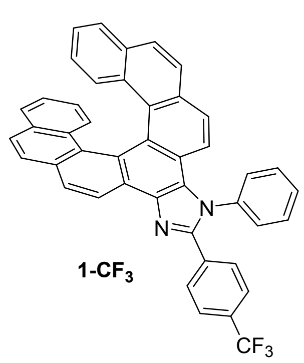

1-CF_3

^{1}H NMR (400 MHz, $CDCl_3$) δ 9.01 (d, *J* = 8.2 Hz, 1H), 8.11 (d, *J* = 8.2 Hz, 1H), 7.84 – 7.71 (m, 6H), 7.67 – 7.55 (m, 5H), 7.52 (d, *J* = 7.9 Hz, 1H), 7.42 (d, *J* = 8.5 Hz, 1H), 7.38 (d, *J* = 8.5 Hz, 1H), 7.34 (d, *J* = 8.5 Hz, 1H), 7.28 (d, *J* = 7.9 Hz, 1H), 7.25 (d, *J* = 8.3 Hz, 1H), 7.11 (d, *J* = 8.5 Hz, 1H), 7.07 (d, *J* = 8.5 Hz, 1H), 6.95 – 6.85 (m, 2H), 6.46 – 6.36 (m, 2H).

^{13}C {^{1}H} NMR (101 MHz, $CDCl_3$) δ 150.1, 138.5, 138.3, 134.1, 131.69, 131.65, 130.52 (2C), 130.51, 130.3, 129.8, 129.59, 129.56 (3C), 129.51, 129.4, 129.19, 129.18, 129.1, 128.6, 127.1, 127.0, 126.5 (2C), 126.4, 126.2, 126.0, 125.30, 125.26, 125.23, 125.19, 125.0 (2C), 124.7 (2C), 124.2, 123.7, 123.5, 123.0, 122.6, 121.5, 121.0, 118.8.

^{19}F NMR (376 MHz, $CDCl_3$) δ -62.76.

R_f = 0.61 (PE/EA 5:1)

HRMS (APCI/QTOF) *m/z* $[M + H]^+$ calculated for $[C_{44}H_{26}N_2F_3]^+$ 639.2043 ; found 639.2041 (100%).

1-(4-(*N*,*N*-Diphenylamino)phenyl)-2-phenyl-1*H*-[7]helicenoimidazole (2-H)

GP was followed with **DK[7]** (30 mg, 1 eq, 0.0735 mmol), ammonium acetate (56 mg, 10 eq), N^1,N^1-diphenylbenzene-1,4-diamine (21 mg, 1.1 eq, 0.0809 mmol), benzaldehyde (8.6 mg, 1.1 eq, 0.0809 mmol), and acetic acid (3 mL). Product **2-H** was obtained after column chromatography on silicagel with EA/PE (10 -> 20 vol%) as a yellow solid (40 mg, 74% yield).

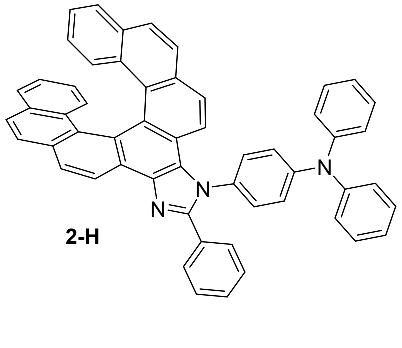

2-H

The same procedure was followed with (*P*)-**DK[7]** (10 mg, 0.024 mmol) yielding 12 mg (0.016 mmol, 66 %) of (*P*)-**2-H** and (*M*)-**DK[7]** (10 mg, 0.024 mmol) yielding 13 mg (0.018 mmol, 72%) of (*M*)-**2-H**.

^1H NMR (400 MHz, $CDCl_3$) δ 9.02 (d, *J* = 8.3 Hz, 1H), 8.09 (d, *J* = 8.3 Hz, 1H), 7.82 – 7.70 (m, 4H), 7.69 – 7.61 (m, 2H), 7.58 (dd, *J* = 8.6, 2.6 Hz, 1H), 7.46 – 7.23 (m, 17H), 7.20 (dd, *J* = 8.6, 2.7 Hz, 1H), 7.18 – 7.06 (m, 4H), 6.95 – 6.86 (m, 2H), 6.41 (dddd, *J* = 15.7, 8.4, 6.9, 1.4 Hz, 2H).

^{13}C {^1H} NMR (101 MHz, $CDCl_3$) δ 152.2, 149.1, 147.1, 138.1, 131.7, 131.64, 131.59, 130.8, 130.4, 129.9, 129.8, 129.7 (4C), 129.60 (2C), 129.55, 129.4, 129.3, 129.2, 129.0, 128.4, 128.3 (2C), 127.0, 126.8, 126.5, 126.33, 126.31, 126.29, 126.0, 125.3, 125.2 (4C), 125.1, 124.9, 124.8, 124.6, 124.1 (2C), 123.8, 123.6, 123.4, 122.9 (2C), 122.7, 121.8, 121.1, 119.0.

R_f = 0.38 (PE/EA 5:1)

HRMS (APCI/QTOF) *m/z* [M + H]$^+$ calculated for $[C_{55}H_{36}N_3]^+$ 738.2904 ; found 738.2902 (100%).

1-(4-(*N*,*N*-Diphenylamino)phenyl)-2-(4-cyanophenyl)-1*H*-[7]helicenoimidazole (2-CN)

GP was followed with **DK[7]** (30 mg, 1 eq, 0.0735 mmol), ammonium acetate (56 mg, 10 eq), N^1,N^1-diphenylbenzene-1,4-diamine (21 mg, 1.1 eq, 0.0809 mmol), 4-cyanobenzaldehyde (10.6 mg, 1.1 eq, 0.0809 mmol), and acetic acid (3 mL). Product **2-CN** was obtained after column chromatography on silica gel with EA/PE (10 -> 20 vol%) as a yellow solid (42 mg, 75% yield).

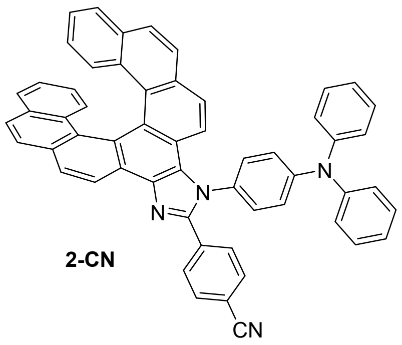

2-CN

The same procedure was followed with (*P*)-**DK[7]** (10 mg, 0.024 mmol) yielding 13 mg (0.017 mmol, 70 %) of (*P*)-**2-CN** and (*M*)-**DK[7]** (10 mg, 0.024 mmol) yielding 14 mg (0.018 mmol, 75%) of (*M*)-**2-CN**.

^1H NMR (400 MHz, $CDCl_3$) δ 8.97 (d, *J* = 8.2 Hz, 1H), 8.10 (d, *J* = 8.2 Hz, 1H), 7.92 (d, *J* = 8.2 Hz, 2H), 7.81 – 7.67 (m, 4H), 7.63 (d, *J* = 8.3 Hz, 2H), 7.56 (dd, *J* = 8.6, 2.5 Hz, 1H), 7.46 – 7.38 (m, 6H), 7.36 (d, *J* = 2.7 Hz, 1H), 7.33 – 7.15 (m, 10H), 7.08 (dd, *J* = 13.1, 8.5 Hz, 2H), 6.91 (t, *J* = 7.4 Hz, 2H), 6.41 (ddd, *J* = 15.3, 8.7, 6.8 Hz, 2H).

^{13}C {^1H} NMR (101 MHz, $CDCl_3$) δ 149.6, 146.8, 138.3, 135.1, 132.0 (2C), 131.70, 131.66, 130.58, 130.56, 130.2, 129.8 (4C), 129.7 (2C), 129.63, 129.59, 129.5, 129.4, 129.2, 128.6, 127.2, 127.1, 126.5 (2C), 126.4, 126.1, 126.0, 125.6 (4C), 125.2, 125.1, 125.0, 124.8, 124.7, 124.5 (2C), 124.4, 123.7, 123.5, 123.1, 122.4 (2C), 121.6, 120.9, 118.9, 118.7, 112.2.

R_f = 0.43 (PE/EA 5:1)

HRMS (ESI/QTOF) *m/z* [M + H]$^+$ calculated for $[C_{56}H_{35}N_4]^+$ 763.2856 ; found 763.2853 (100%).

1-(4-(*N*,*N*-Diphenylamino)phenyl)-2-(4-(trifluoromethyl)phenyl)-1*H*-[7]helicenoimidazole (2-CF_3)

GP was followed with **DK[7]** (30 mg, 1 eq, 0.0735 mmol), ammonium acetate (56 mg, 10 eq), N^1,N^1-diphenylbenzene-1,4-diamine (21 mg, 1.1 eq, 0.0809 mmol), 4-(trifluoromethyl)benzaldehyde (14 mg, 1.1 eq, 0.0809 mmol), and acetic acid (3 mL). Product **2-CF_3** was obtained after column chromatography on silica gel with EA/PE (0 -> 10 vol%) as a yellow solid (35 mg, 59% yield).

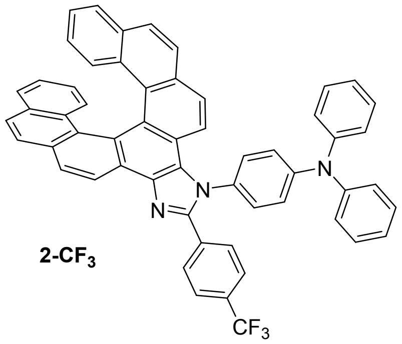

2-CF_3

^1H NMR (400 MHz, CD_2Cl_2) δ 8.95 (d, *J* = 8.2 Hz, 1H), 8.12 (d, *J* = 8.3 Hz, 1H), 7.96 (d, *J* = 8.1 Hz, 2H), 7.83 – 7.74 (m, 2H), 7.74 – 7.65 (m, 4H), 7.60 (dd, *J* = 8.6, 2.6 Hz, 1H), 7.46 – 7.14 (m, 17H), 7.07 (t, *J* = 9.4 Hz, 2H), 6.91 (t, *J* = 7.4 Hz, 2H), 6.45 – 6.36 (m, 2H).

^{13}C {^1H} NMR (101 MHz, $CDCl_3$) δ 150.4, 149.4, 146.9, 138.2, 134.3, 131.69, 131.66, 130.9, 130.5, 130.4, 129.9, 129.7, 129.64, 129.62, 129.61, 129.5, 129.4, 129.2, 128.6, 127.1, 127.0, 126.5, 126.44, 126.38, 126.2, 126.0, 125.5, 125.3, 125.23, 125.20, 125.16, 125.0, 124.8, 124.7, 124.4, 124.2, 123.7, 123.5, 122.9, 122.6, 121.6, 121.0, 119.0, 29.70..

^{19}F {^1H} NMR (376 MHz, $CDCl_3$) δ -62.67.

R_f = 0.57 (PE/EA 10:1)

HRMS (APCI/QTOF) *m/z* $[M + H]^+$ calculated for $[C_{56}H_{35}F_3N_3]^+$ 806.2778 ; found 806.2779 (100%).

1-(9H-Carbazol-9-yl)-phenyl-4-ene-2-phenyl-1*H*-[7]helicenoimidazole (3-H)

GP was followed with **DK[7]** (30 mg, 1 eq, 0.0734 mmol), ammonium acetate (56.6 mg, 10 eq, 0.7345), 4-(9H-carbazol-9-yl)aniline (20.9 mg, 1.1 eq, 0.0808 mmol), benzaldehyde (8.6 mg, 1.1 eq, 0.0808 mmol), and acetic acid (3 mL). Product **3-H** was obtained after column chromatography on silica gel with EA/PE (10 -> 30 vol%) as a yellow solid (39 mg, 72% yield).

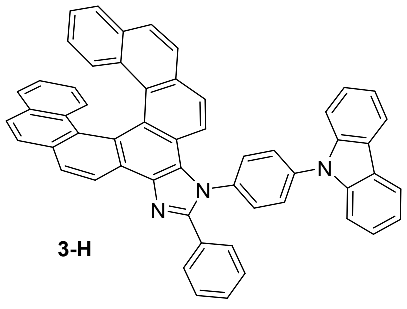

3-H

The same procedure was followed with (*M*)- **DK[7]** (9 mg, 0.0220 mmol) and 7 mg (43% yield) of (*M*)-**3-H** was obtained. The same procedure was followed with (*P*)-**DK[7]** (9 mg, 0.0220 mmol) and 13 mg (80% yield) of (*P*)-**3-H** was obtained.

^1H NMR (400 MHz, $CDCl_3$) δ 9.07 (d, *J* = 9.2 Hz, 1H), 8.23 (d, *J* = 7.8 Hz, 2H), 8.13 (d, *J* = 8.3 Hz, 1H), 8.01 (dd, *J* = 8.3, 2.4 Hz, 1H), 7.93 (dd, *J* = 8.3, 2.3 Hz, 1H), 7.84 – 7.68 (m, 6H), 7.67 – 7.51 (m, 6H), 7.50 – 7.36 (m, 7H), 7.33 – 7.26 (m, 2H), 7.15 (dd, *J* = 13.6, 8.5 Hz, 2H), 6.92 (tdd, *J* = 6.7, 2.6, 1.1 Hz, 2H), 6.50 – 6.36 (m, 2H).

^{13}C {^1H} NMR (101 MHz, $CDCl_3$) δ 152.1, 140.4, 139.1, 138.3, 137.3, 131.7, 130.9, 130.8, 130.5, 129.7, 129.6 (2C), 129.4, 129.24, 129.16, 128.6, 128.44 (2C), 128.38, 128.35, 127.2, 127.0, 126.5, 126.44, 126.38, 126.32 (2C), 126.25, 126.0, 125.3, 125.0, 124.8, 124.7, 124.0, 123.8, 123.7, 123.5, 122.9, 121.5, 121.1, 120.7, 120.6, 118.7, 109.5 (2C)..

R_f = 0.29 (PE/EA 8:1)

HRMS (APCI/QTOF) *m/z* $[M + H]^+$ calculated for $[C_{55}H_{34}N_3]^+$ 736.2747 ; found 736.2745 (100%).

1-(9H-Carbazol-9-yl)-phenyl-4-ene-2-(4-cyanophenyl)-1*H*-[7]helicenoimidazole (3-CN)

GP was followed with **DK[7]** (30 mg, 1 eq, 0.0734 mmol), ammonium acetate (56.6 mg, 10 eq, 0.7345), 4-(9H-carbazol-9-yl)aniline (20.9 mg, 1.1 eq, 0.0808 mmol), 4-cyanobenzaldehyde (10. 6mg, 1.1 eq, 0.0808 mmol), and acetic acid (3 mL). Product **3-CN** was obtained after column chromatography on silica gel with EA/PE (10 -> 30 vol%) as a yellow solid (47 mg, 84% yield).

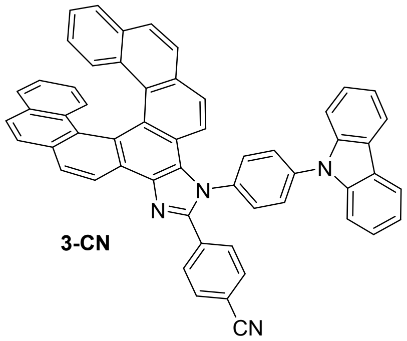

3-CN

The same procedure was followed with (*M*)-**DK[7]**(9 mg, 0.0220 mmol) and 11 mg (66% yield) of (*M*)-**3-CN** was obtained. The same

procedure was followed with (*P*)-**DK**[7] (9 mg, 0.0220 mmol) and 12 mg (72% yield) of (*P*)-**3-CN** was obtained.

^{1}H NMR (400 MHz, $CDCl_3$) δ 9.01 (d, *J* = 8.2 Hz, 1H), 8.24 (d, *J* = 7.8 Hz, 2H), 8.14 (d, *J* = 8.3 Hz, 1H), 8.10 – 7.99 (m, 2H), 7.98 – 7.85 (m, 3H), 7.81 – 7.69 (m, 5H), 7.66 – 7.51 (m, 6H), 7.46 – 7.39 (m, 4H), 7.31 – 7.27 (m, 2H), 7.11 (d, *J* = 8.4 Hz, 1H), 7.08 (d, *J* = 8.5 Hz, 1H), 6.92 (dddd, *J* = 8.0, 6.9, 2.8, 1.2 Hz, 2H), 6.43 (dddd, *J* = 17.0, 8.4, 6.8, 1.4 Hz, 2H).

^{13}C {^{1}H} NMR (101 MHz, $CDCl_3$) δ 149.5, 140.3, 140.0, 138.6, 136.8, 135.7, 134.8, 132.2, 131.74, 131.72, 130.8, 130.70, 130.67, 130.1, 129.8, 129.73, 129.71, 129.6, 129.3, 129.2, 128.8, 128.70, 128.69, 127.4, 127.3, 126.7, 126.6, 126.5, 126.0, 125.9, 125.21, 125.18, 125.0, 124.9, 124.7, 124.0, 123.8, 123.6, 123.2, 121.2, 120.9, 120.8, 118.6, 118.4, 112.6, 109.4.

R_f = 0.16 (PE/EA 8:1)

HRMS (APCI/QTOF) *m/z* [M + H]$^+$ calculated for $[C_{56}H_{33}N_4]^+$ 761.2700 ; found 761.2702 (100%).

1-(9H-carbazol-9-yl)-phenyl-4-ene-2-(4-(trifluoromethyl)phenyl)-1H-[7]helicenoimidazole (3-CF$_3$)

GP was followed with **DK[7]** (30 mg, 1 eq, 0.0734 mmol), ammonium acetate (56.6 mg, 10 eq, 0.7345), 4-(9H-carbazol-9-yl)aniline (20.9 mg, 1.1 eq, 0.0808 mmol), 4-(trifluoromethyl)benzaldehyde (14.1 mg, 1.1 eq, 0.0808 mmol) and acetic acid (3 mL). Product **3-CF$_3$** was obtained after column chromatography on silica gel with EA/PE (10 -> 30 vol%) as a yellow solid (44 mg, 75% yield).

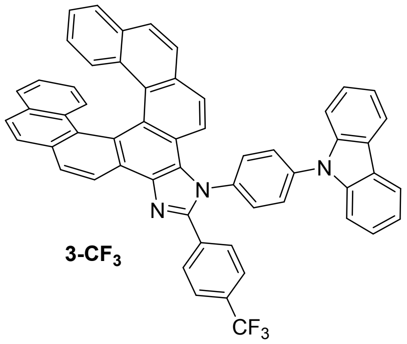

3-CF$_3$

^{1}H NMR (400 MHz, $CDCl_3$) δ 9.03 (d, *J* = 8.2 Hz, 1H), 8.23 (d, *J* = 7.8 Hz, 2H), 8.14 (d, *J* = 8.2 Hz, 1H), 8.08 – 7.97 (m, 2H), 7.92 (d, *J* = 8.1 Hz, 2H), 7.87 (dd, *J* = 8.3, 2.3 Hz, 1H), 7.80 – 7.73 (m, 3H), 7.70 (d, *J* = 8.2 Hz, 2H), 7.65 – 7.53 (m, 6H), 7.46 – 7.37 (m, 4H), 7.32 – 7.26 (m, 2H), 7.18 – 7.06 (m, 2H), 6.98 – 6.86 (m, 2H), 6.43 (dddd, *J* = 16.8, 8.4, 6.9, 1.4 Hz, 2H).

^{13}C {^{1}H} NMR (101 MHz, $CDCl_3$) δ 150.24, 140.33, 139.54, 138.44, 136.90, 133.97, 131.72 (q, J_{CF} = 1 Hz), 130.84, 130.78 (q, J_{CF} = 33 Hz), 130.73, 130.61, 129.80, 129.71, 129.61, 129.58, 129.36, 129.19, 128.73, 128.58, 128.55, 127.36, 127.18, 126.61, 126.56, 126.43, 126.41, 126.13, 125.95, 125.42 (q, J_{CF} = 3.7 Hz), 125.24, 125.12, 124.98, 124.82, 124.72, 124.44, 123.92, 123.91 (q, J_{CF} = 272.5 Hz), 123.77, 123.53, 123.10, 121.32, 120.97, 120.82, 120.70, 118.63, 109.46.

^{19}F NMR (376 MHz, $CDCl_3$) δ -62.73.

R_f = 0.51 (PE/EA 8:1)

HRMS (APCI/QTOF) *m/z* [M + H]$^+$ calculated for $[C_{56}H_{33}F_3N_3]^+$ 804.2621; found 804.2619 (100%).

3. NMR Spectra

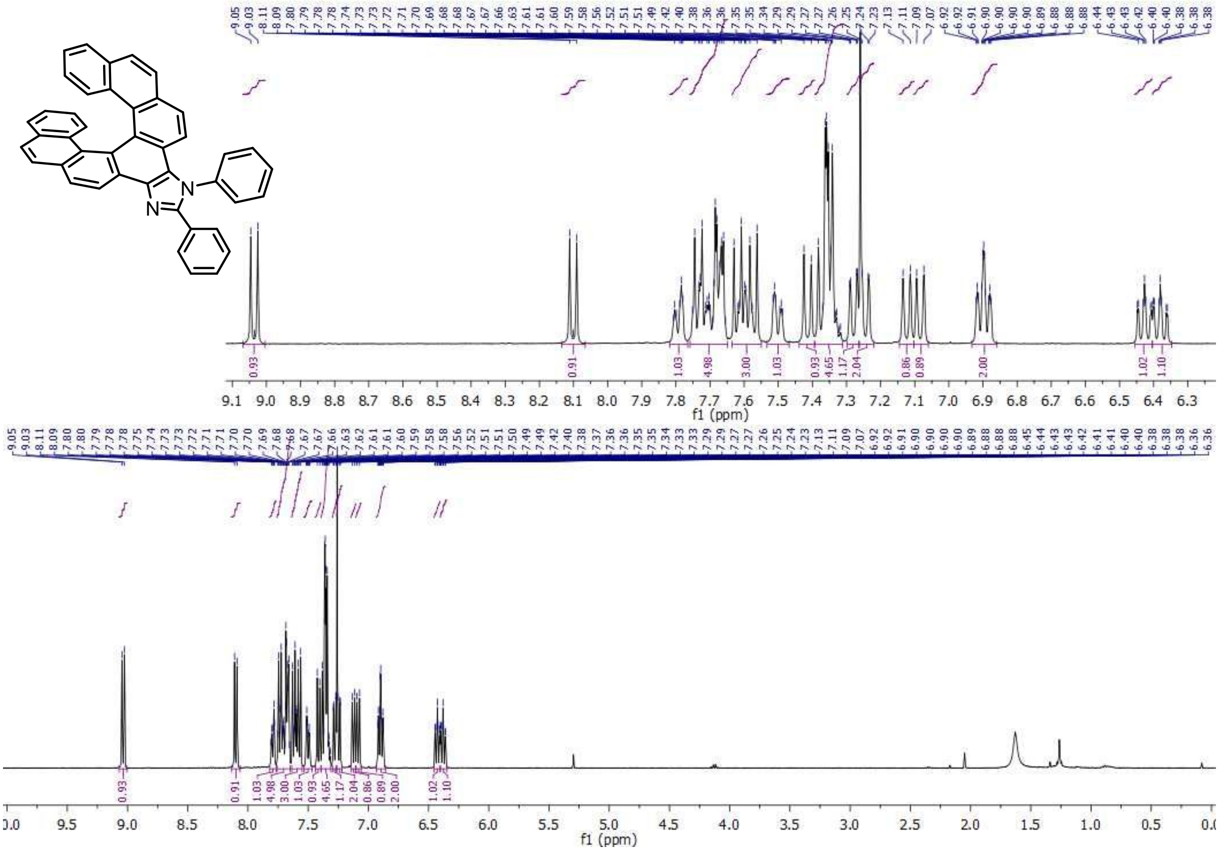

Figure S1 *^{1}H NMR spectrum of **1-H** (400 MHz, $CDCl_3$)*

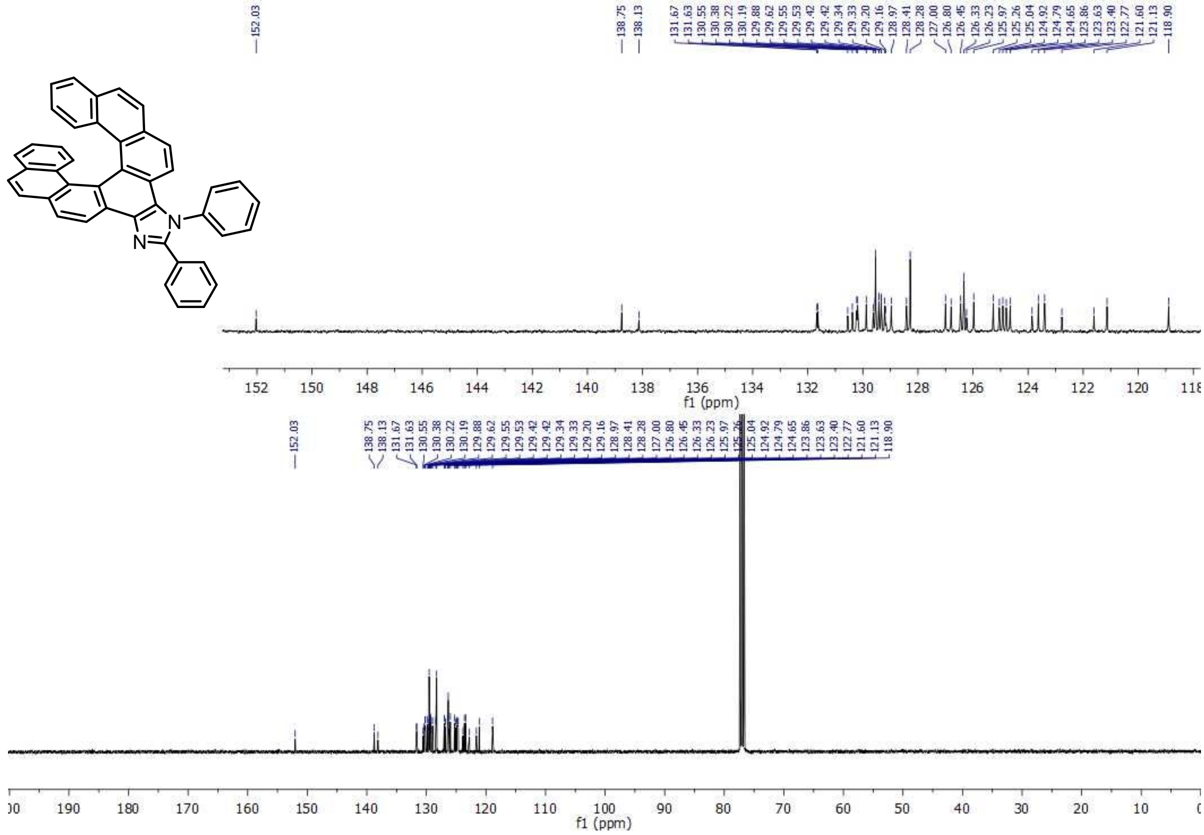

Figure S2 *^{13}C {^{1}H} NMR spectrum of* ***1-H*** *(101 MHz, $CDCl_3$)*

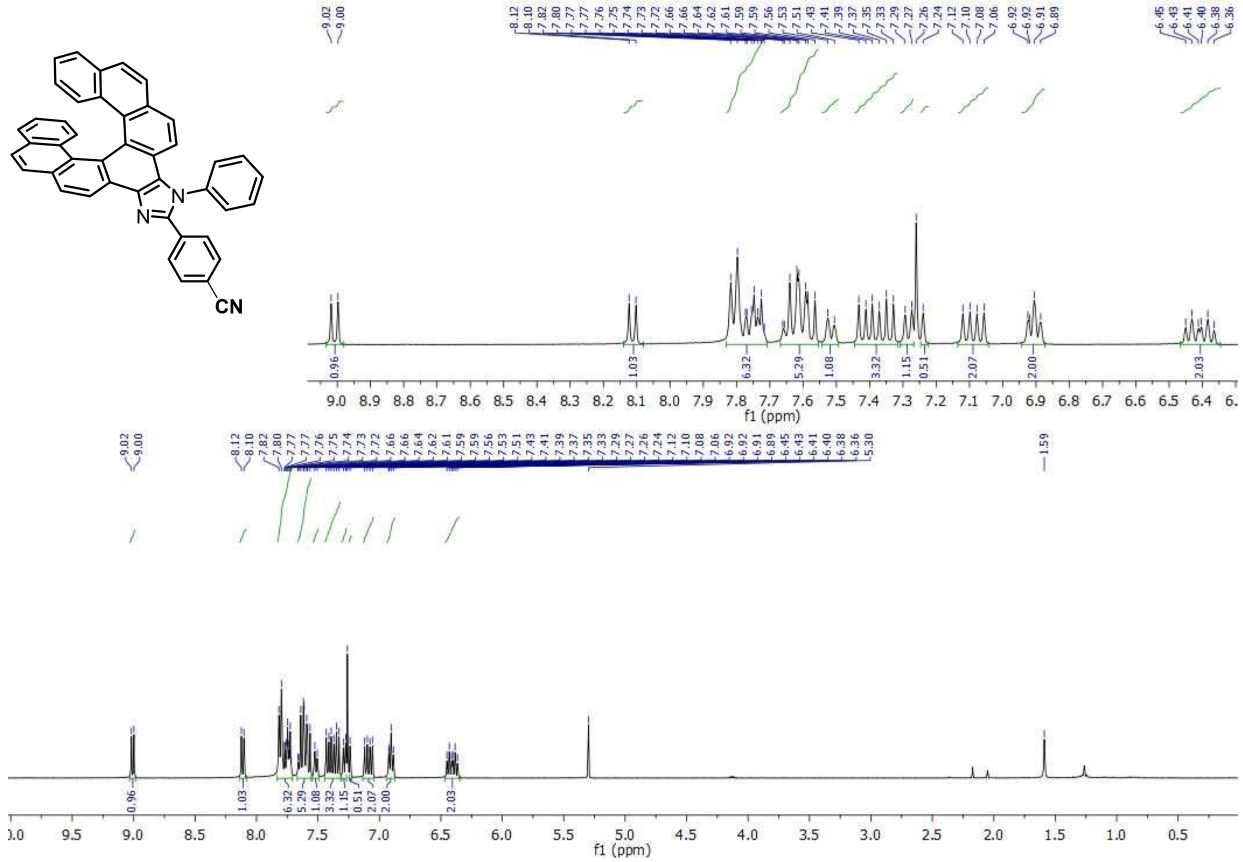

Figure S3 *^{1}H NMR spectrum of* ***1-CN*** *(400 MHz, $CDCl_3$)*

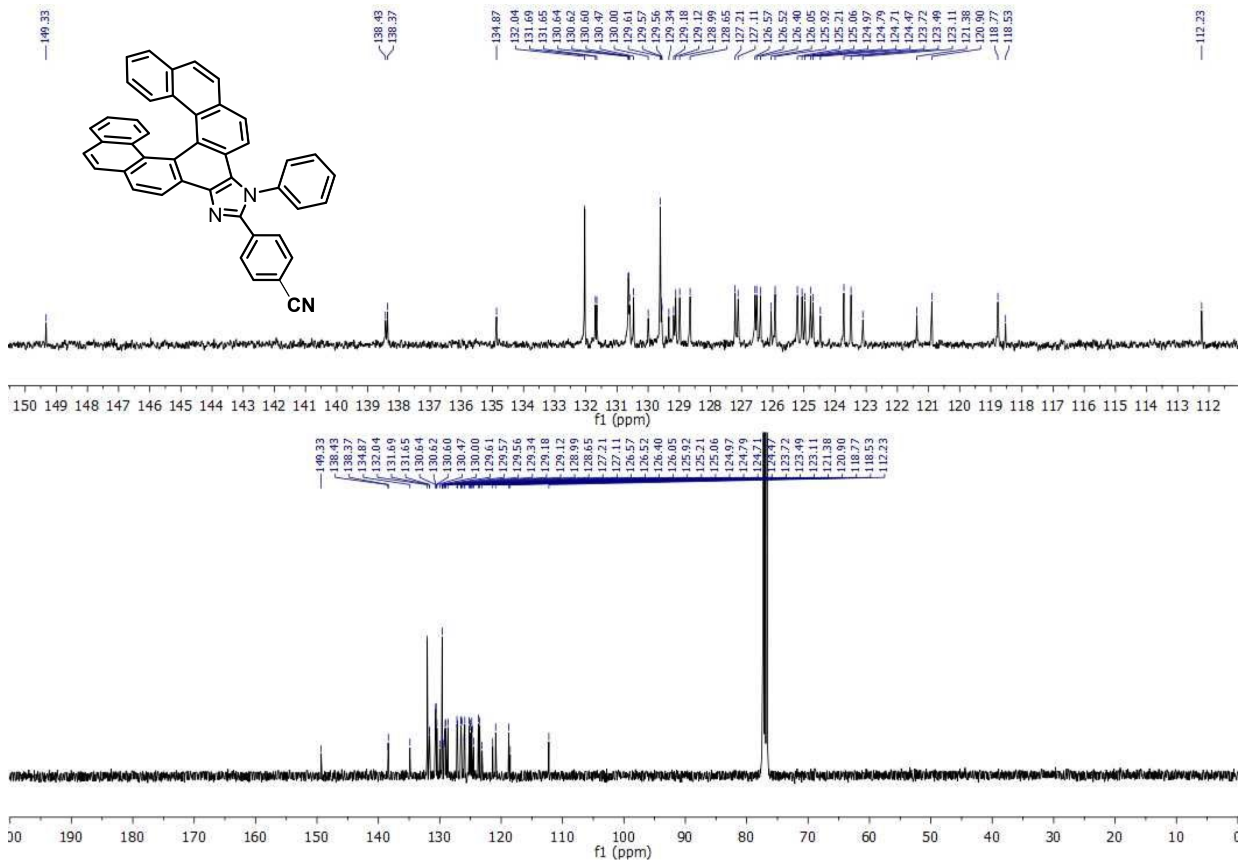

Figure S4 ^{13}C $\{^{1}H\}$ *NMR spectrum of* ***1-CN*** *(101 MHz, $CDCl_3$)*

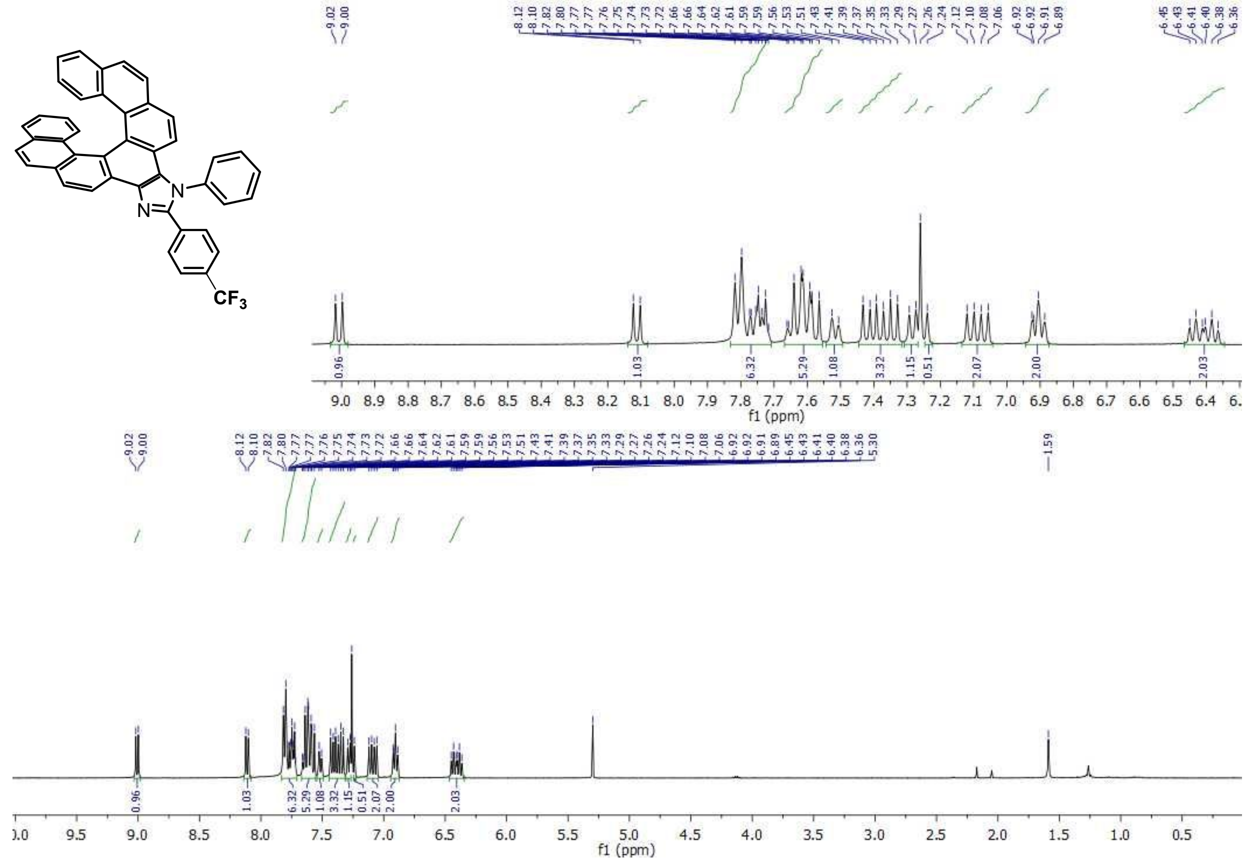

Figure S5 ^{1}H *NMR spectrum of* ***1-CF₃*** *(400 MHz, $CDCl_3$)*

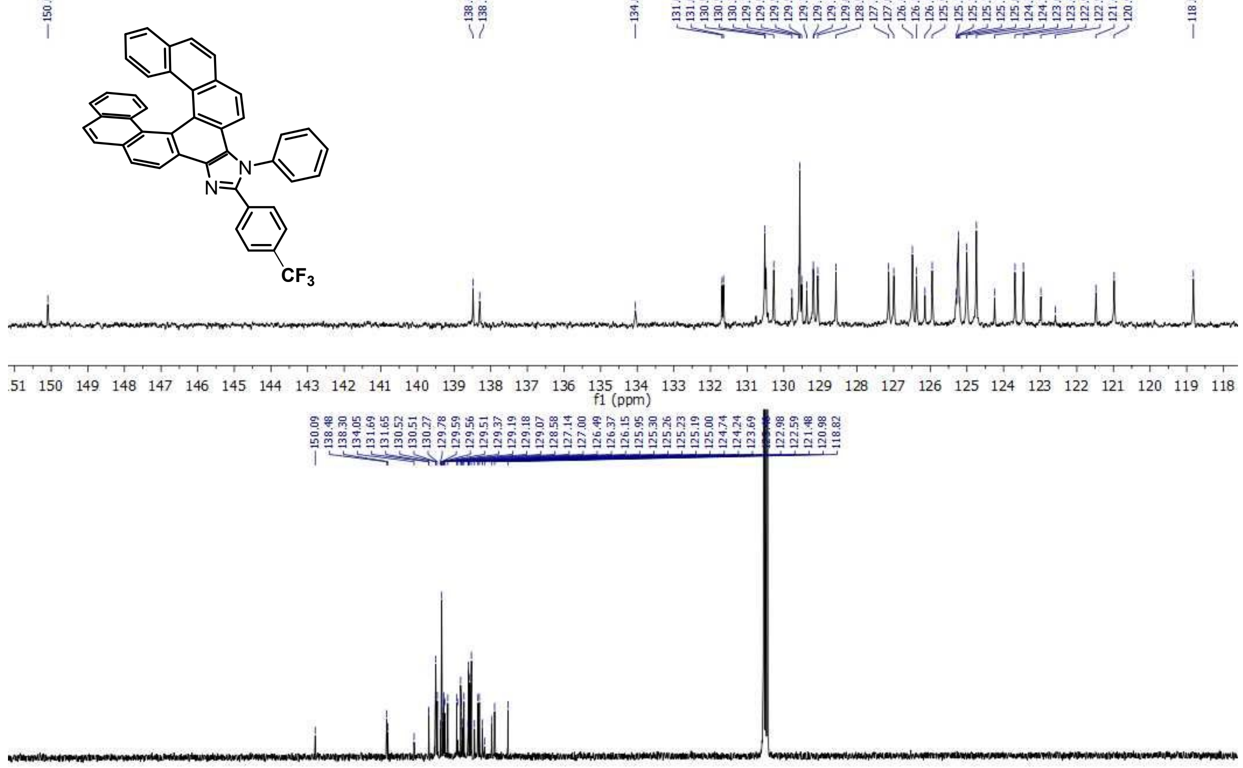

Figure S6 *^{13}C {^{1}H} NMR spectrum of* ***1-CF₃*** *(101 MHz, $CDCl_3$)*

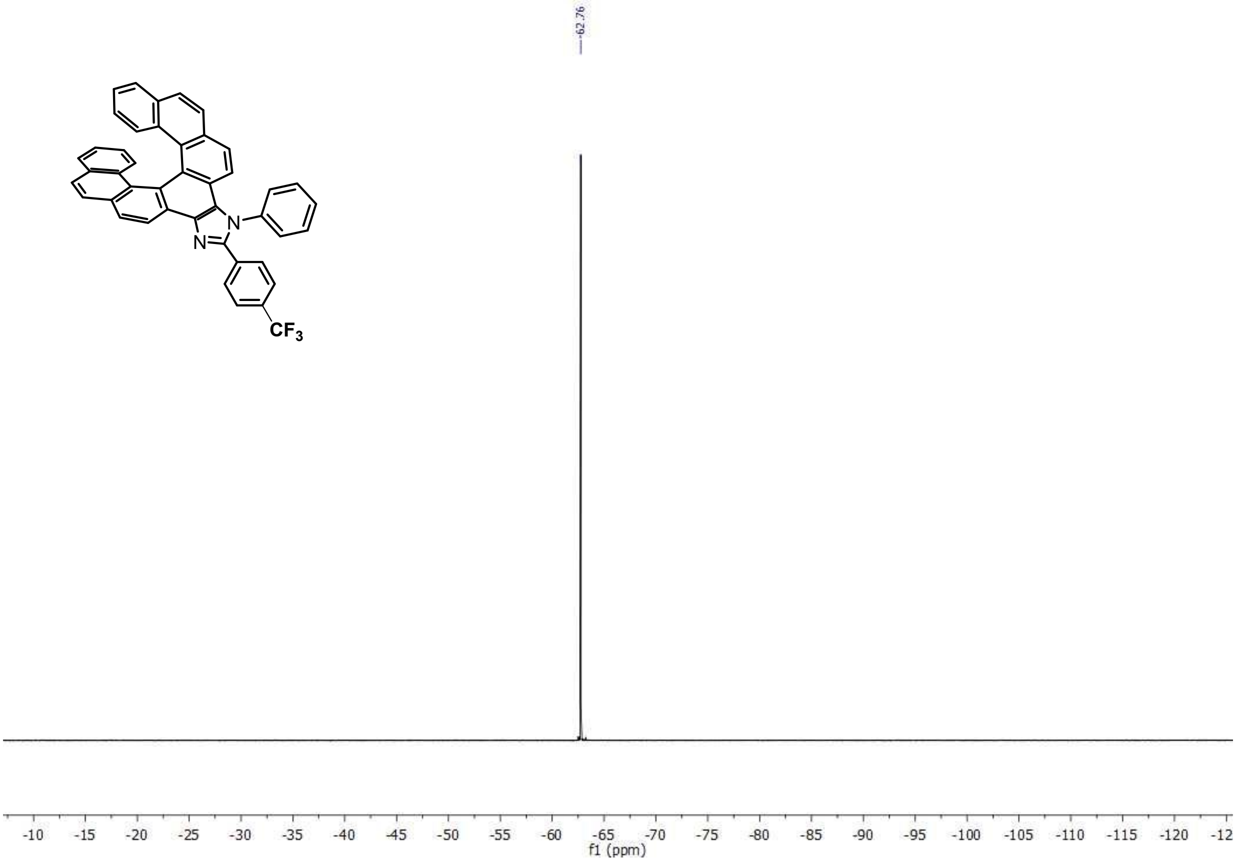

Figure S7 ^{19}F NMR spectrum of **1-CF₃** (376 MHz, $CDCl_3$)

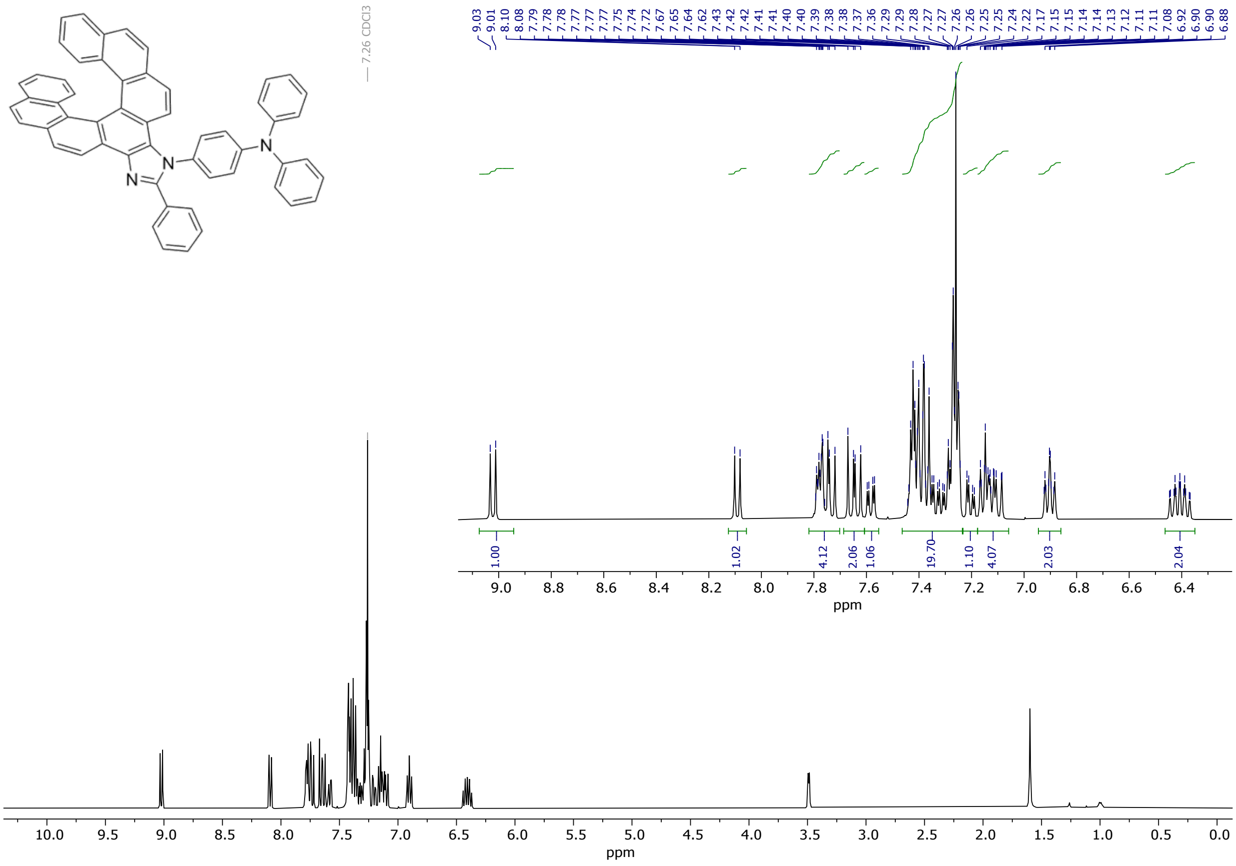

Figure S8 ^{1}H *NMR spectrum of* ***2-H*** *(400 MHz, $CDCl_3$)*

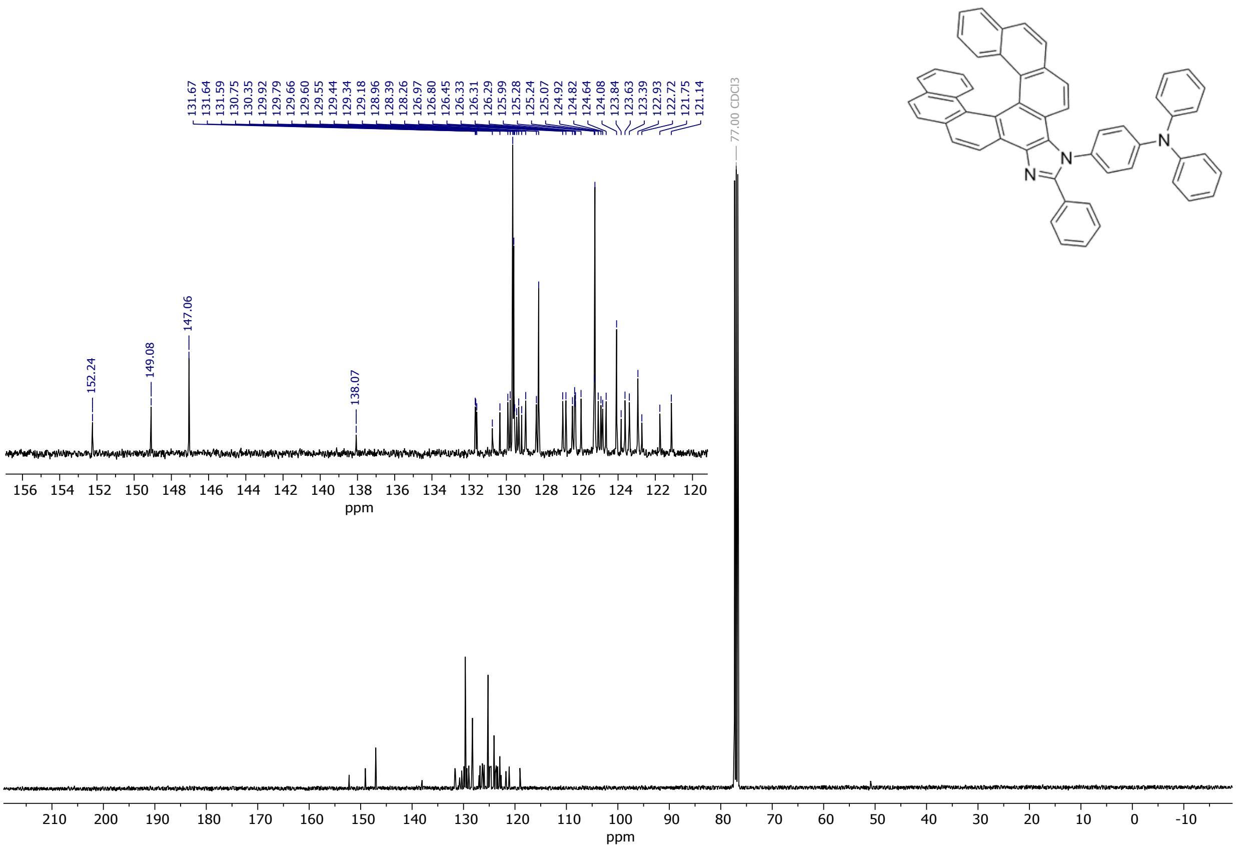

Figure S9 ^{13}C *{^{1}H} NMR spectrum of* ***2-H*** *(101 MHz, $CDCl_3$)*

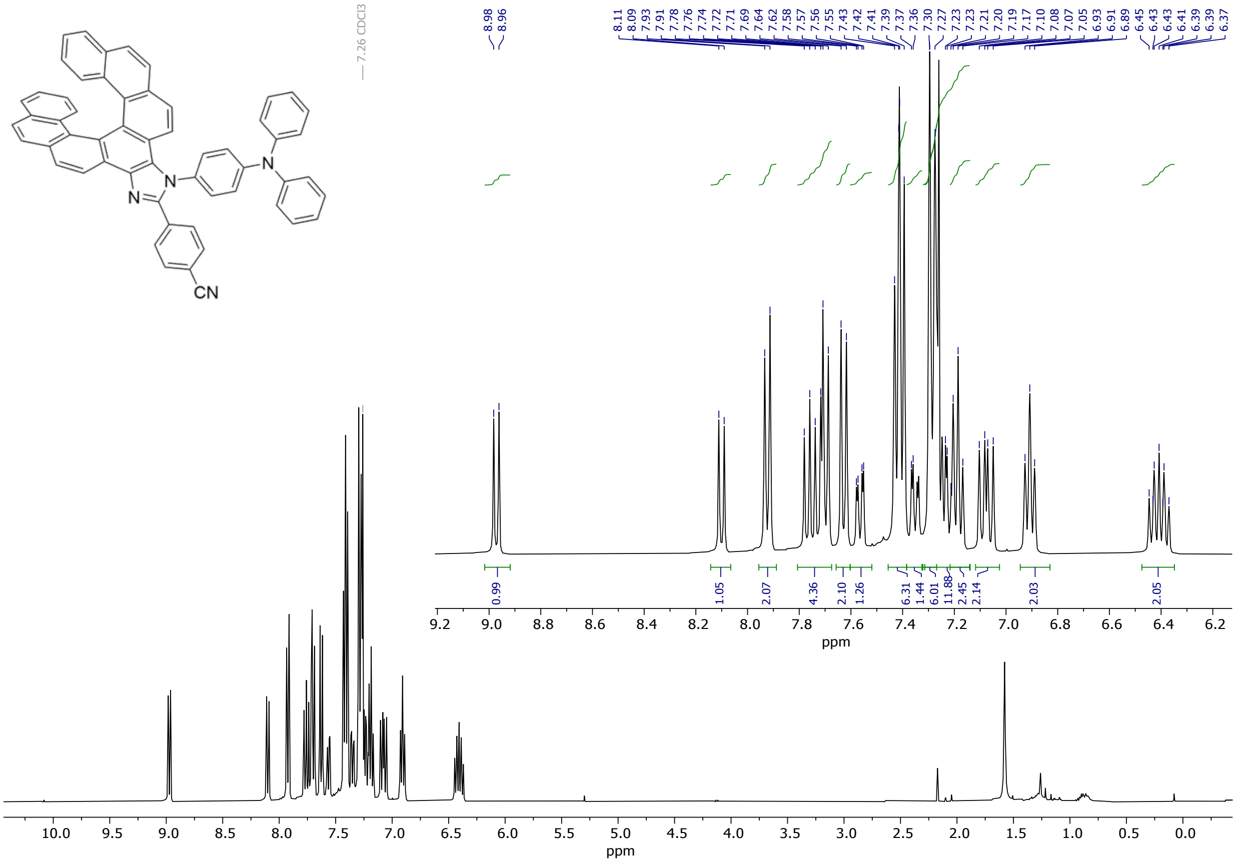

Figure S10 ^{1}H NMR spectrum of **2-CN** (400 MHz, $CDCl_3$)

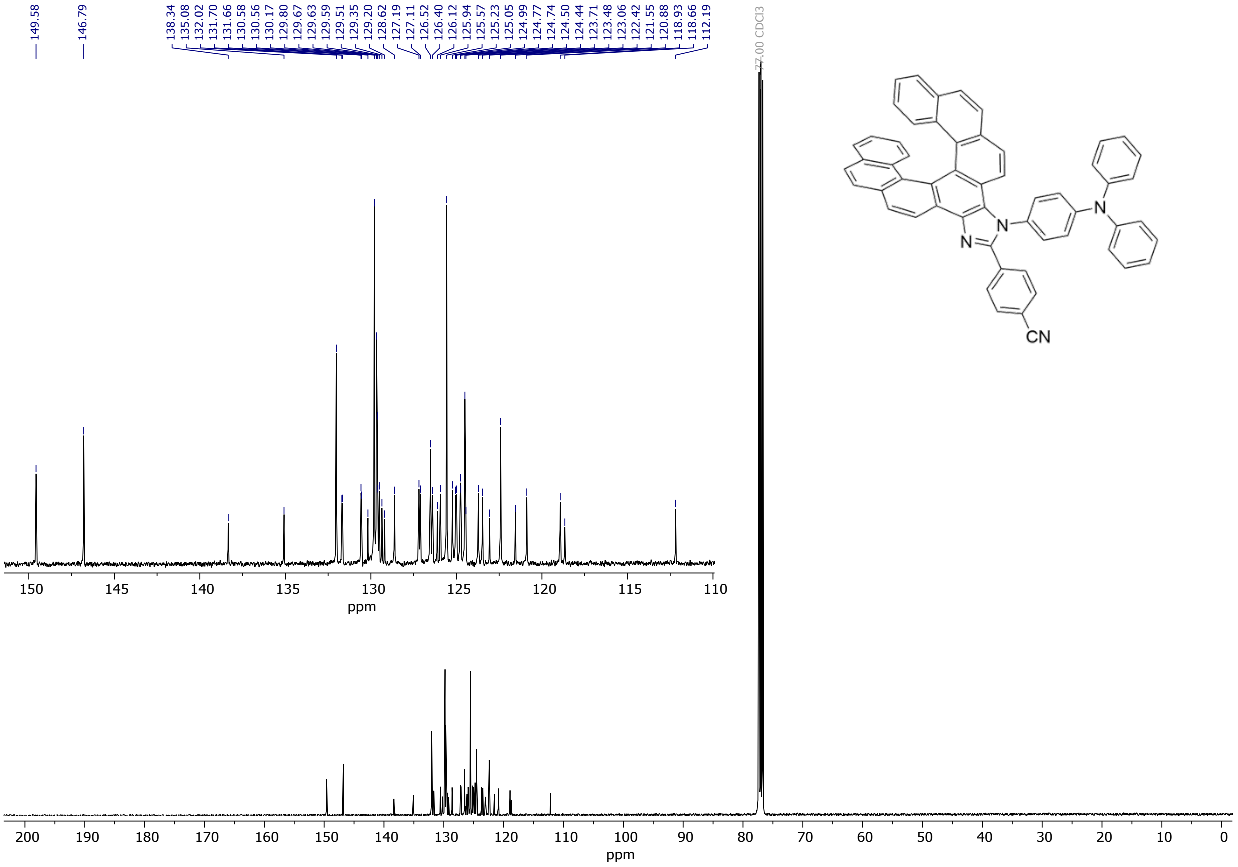

Figure S11 ^{13}C {^{1}H} NMR spectrum of **2-CN** (101 MHz, $CDCl_3$)

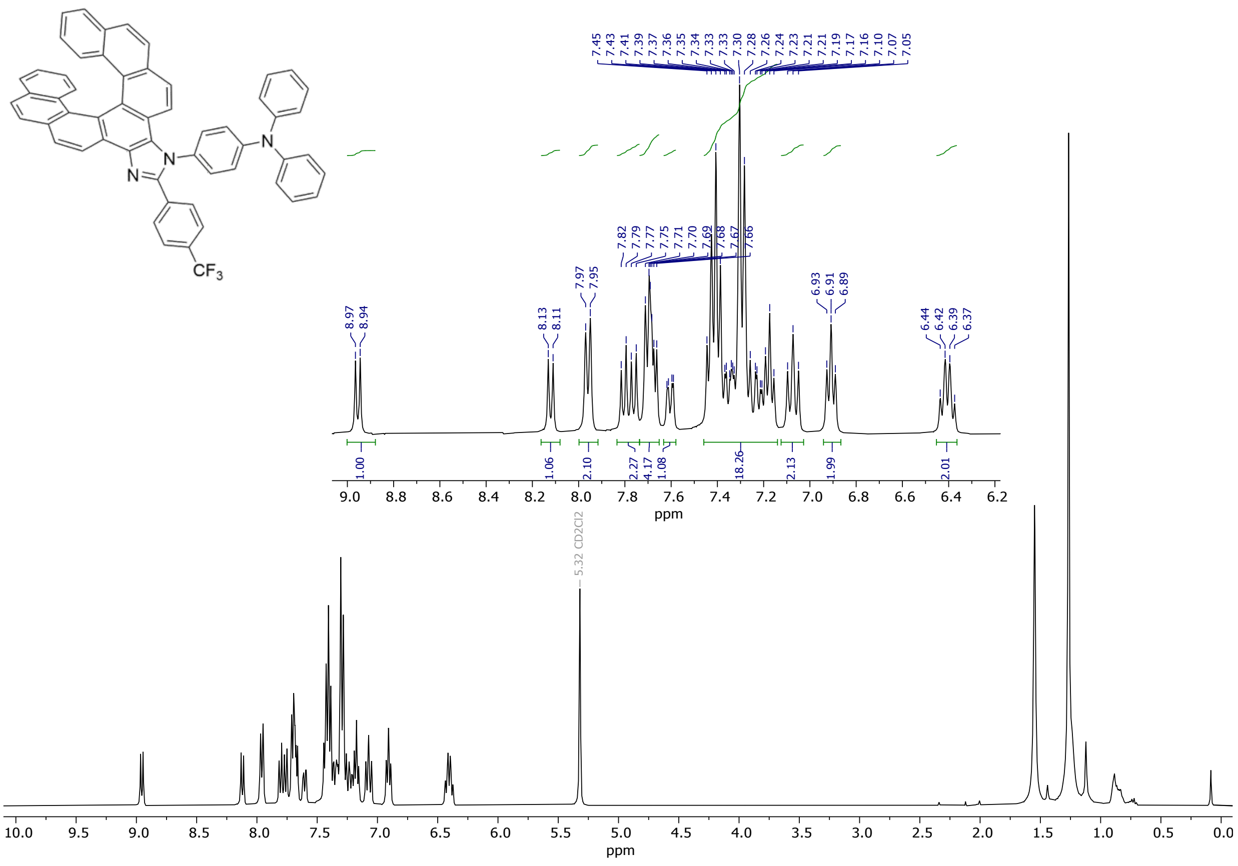

Figure S12 ^{1}H NMR spectrum of **2-CF$_3$** (400 MHz, CD_2Cl_2)

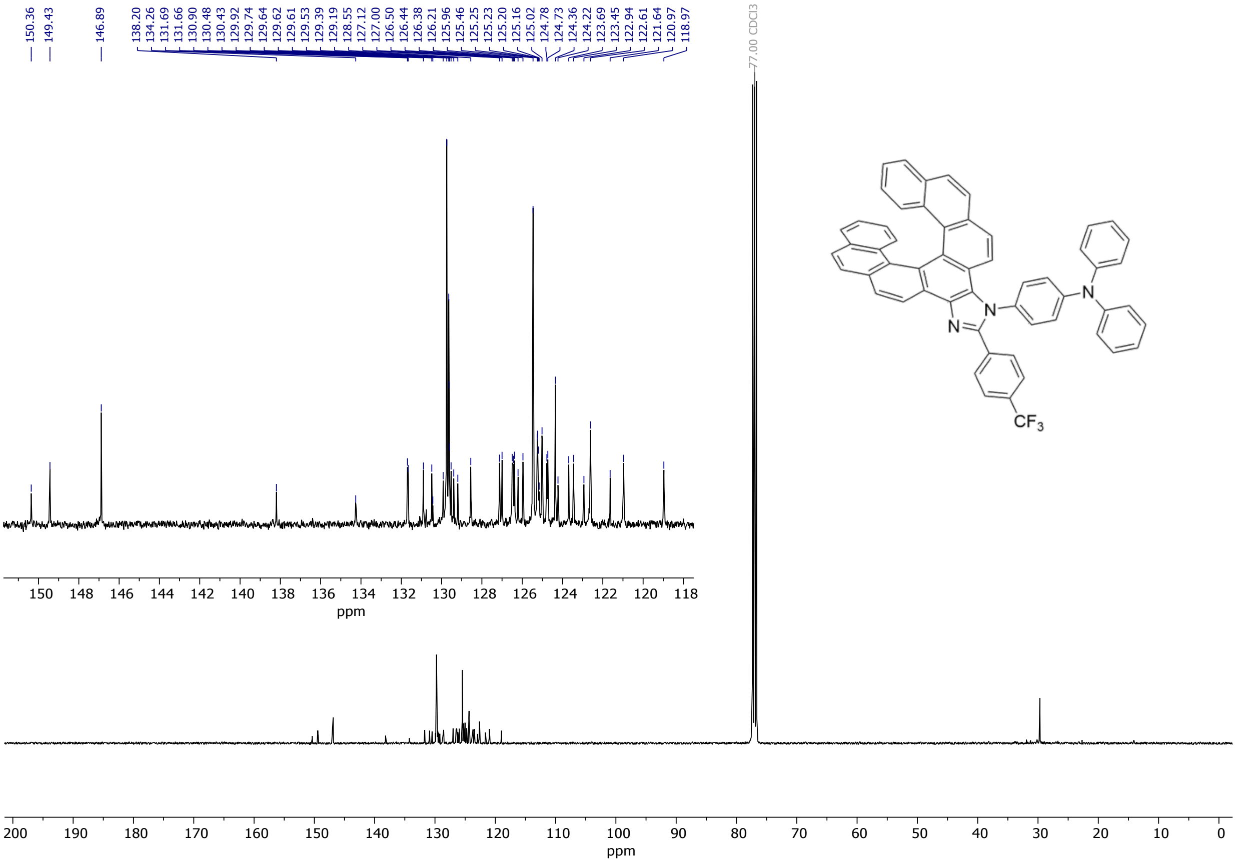

Figure S13 ^{13}C {^{1}H} NMR spectrum of **2-CF$_3$** (101 MHz, $CDCl_3$)

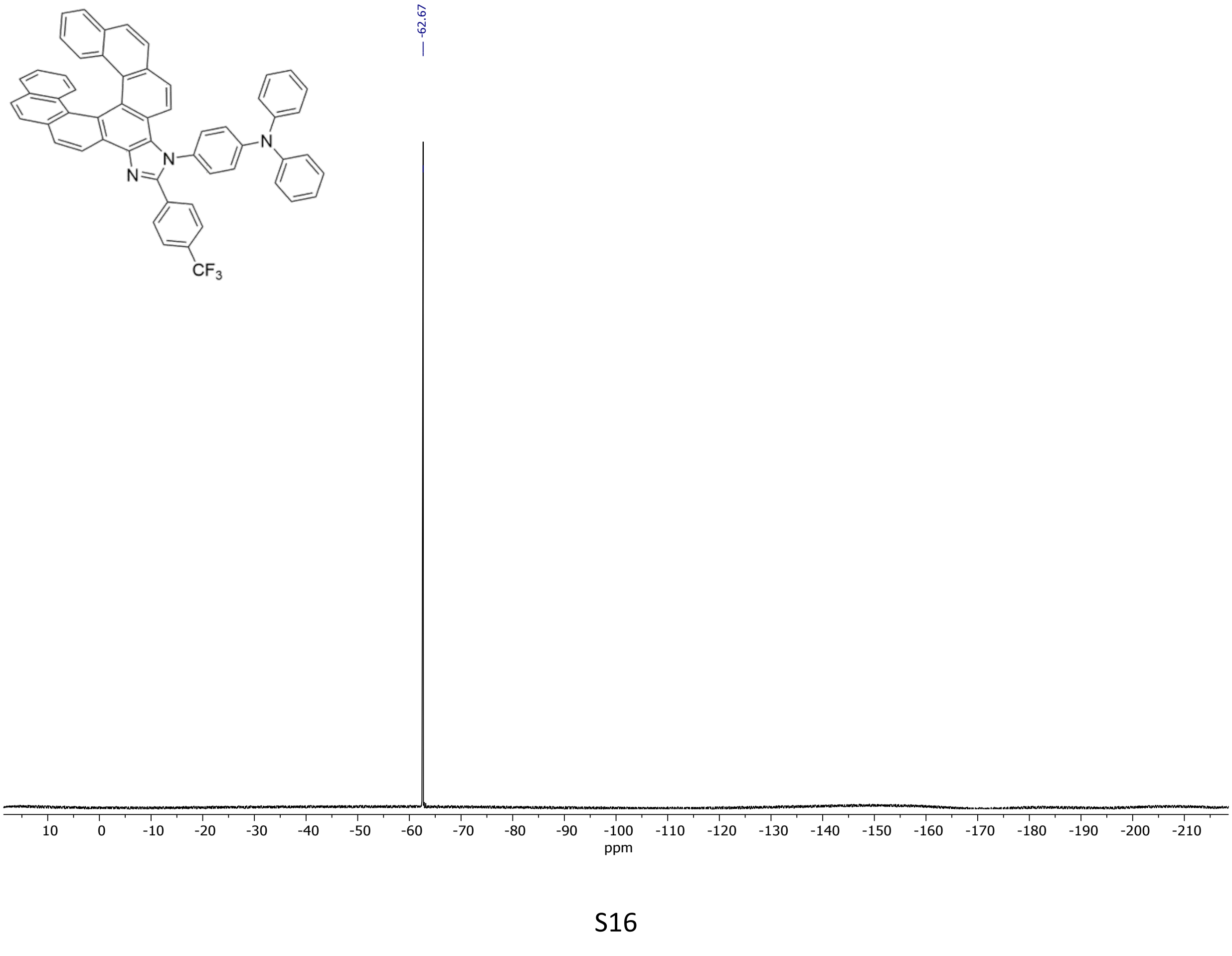

Figure S14 ^{19}F NMR spectrum of **2-CF$_3$** (376 MHz, CDCl$_3$)

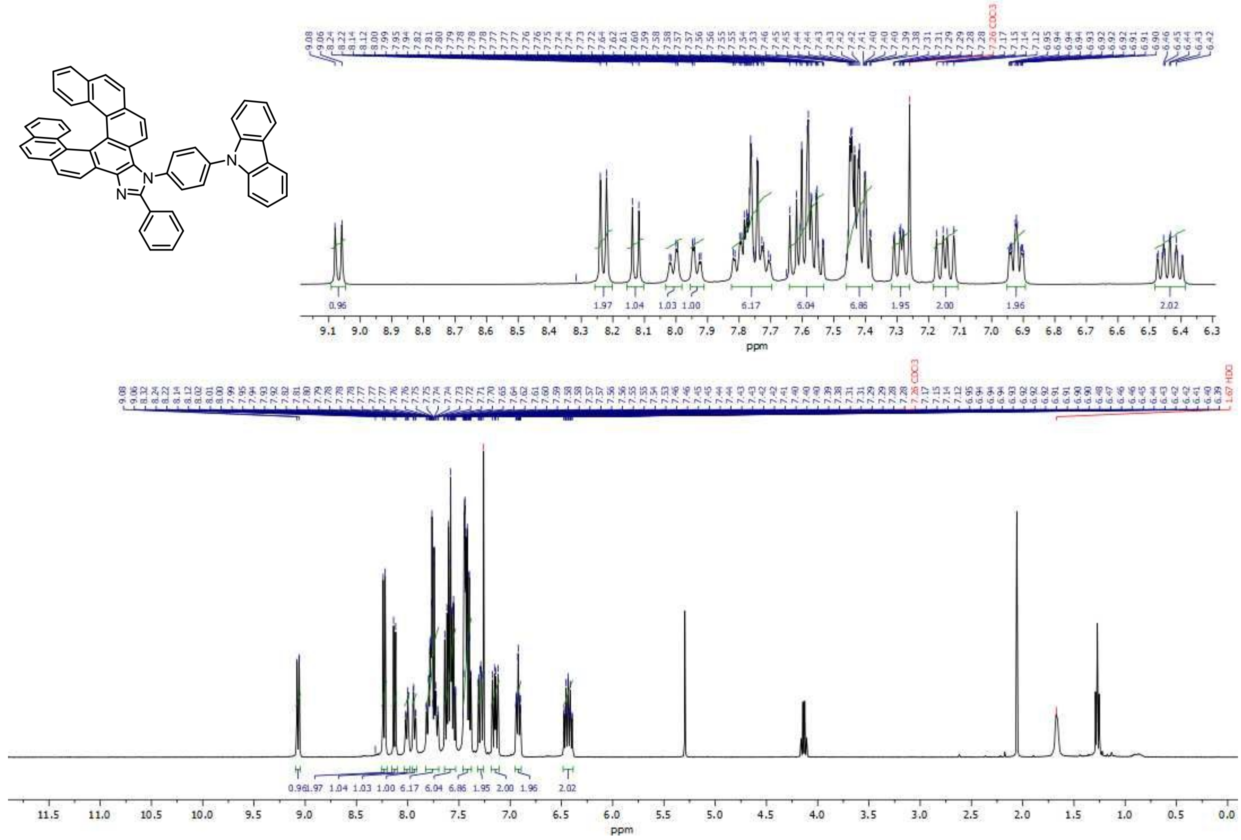

Figure S15 ^{1}H NMR spectrum of **3-H** (400 MHz, CDCl$_3$)

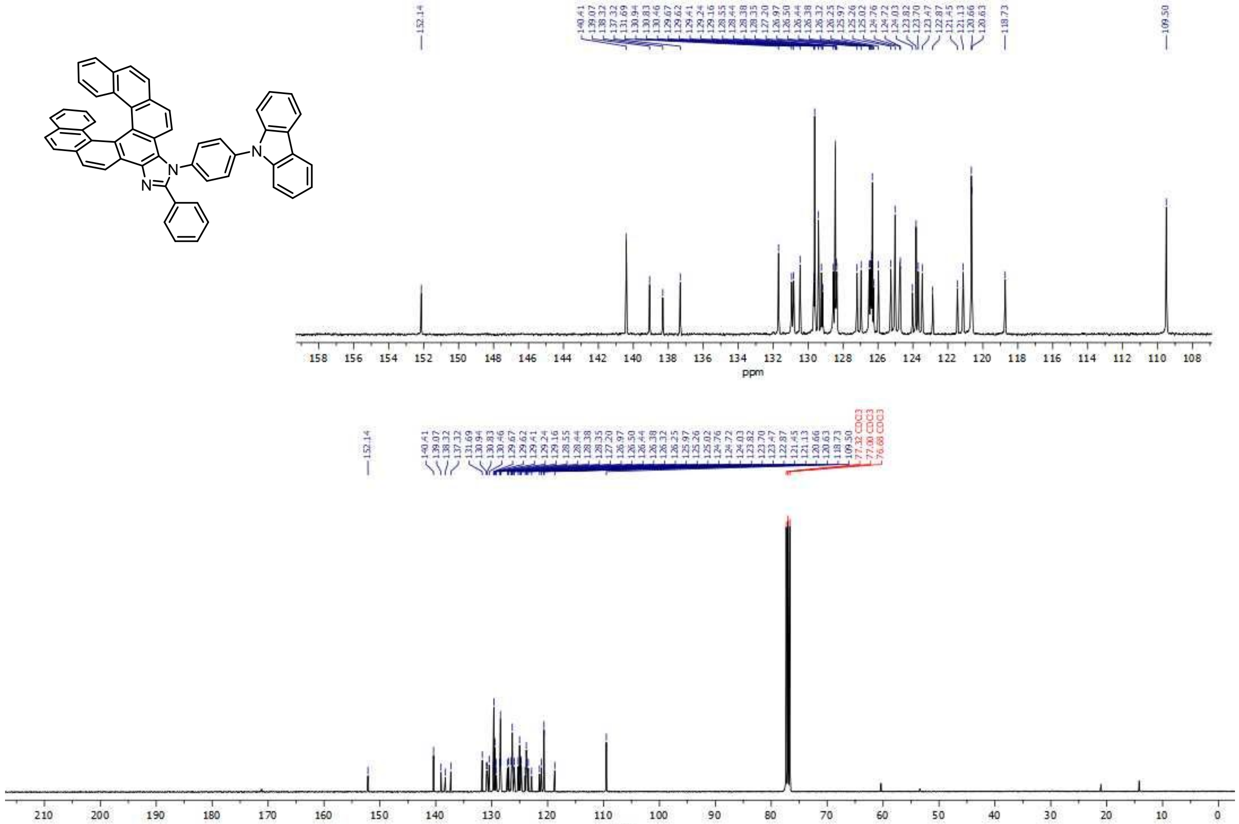

Figure S16 ^{13}C {^{1}H} NMR spectrum of **3-H** (101 MHz, CDCl$_3$)

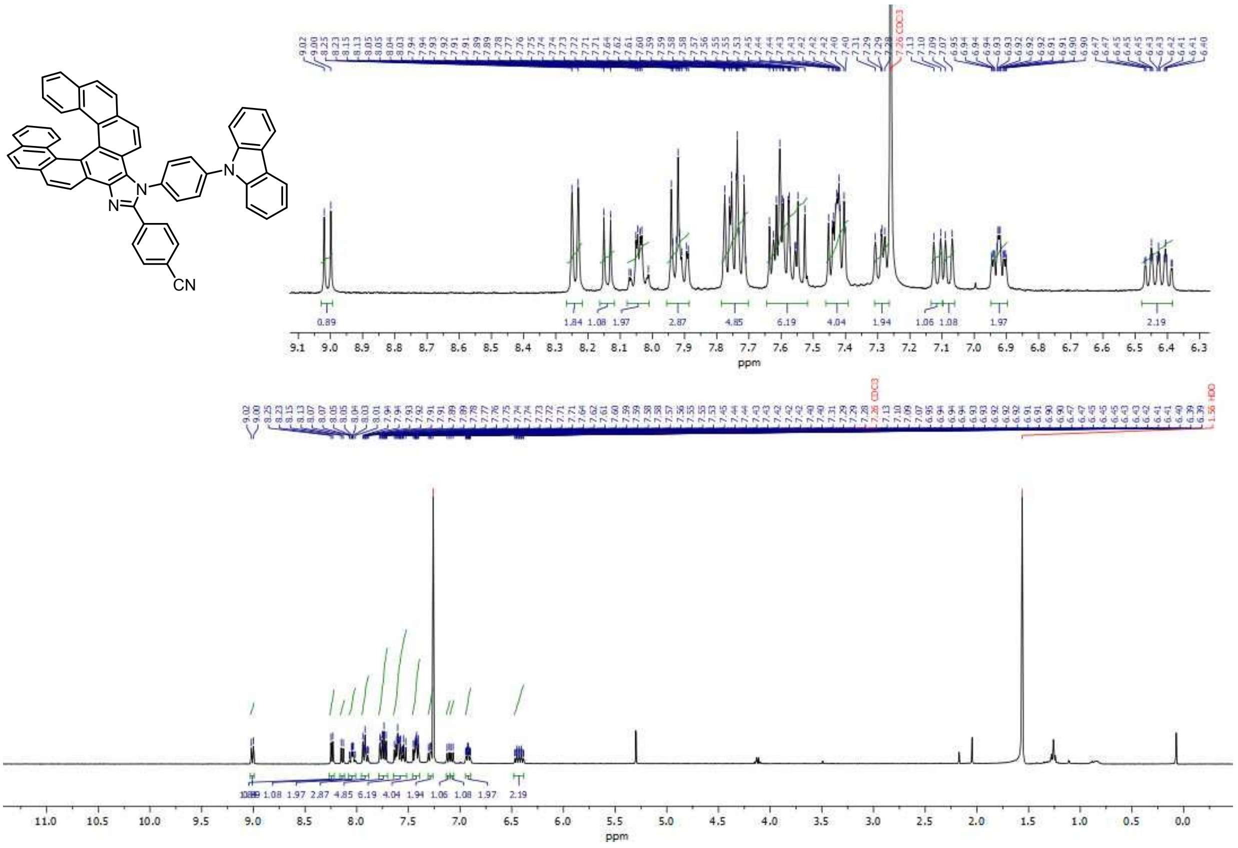

Figure S17 ^{1}*H NMR spectrum of* ***3-CN*** *(400 MHz, $CDCl_3$)*

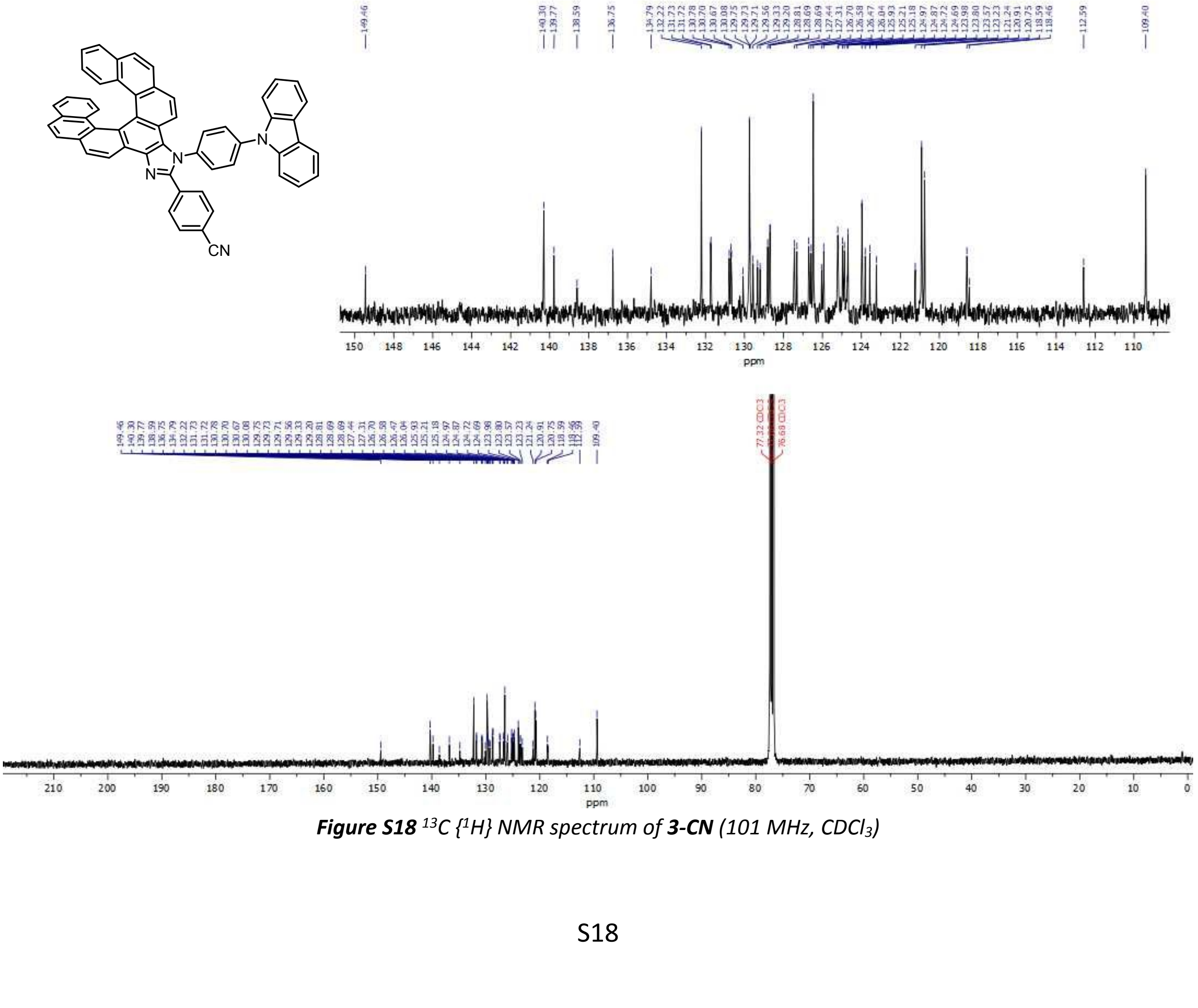

Figure S18 ^{13}*C {^{1}H} NMR spectrum of* ***3-CN*** *(101 MHz, $CDCl_3$)*

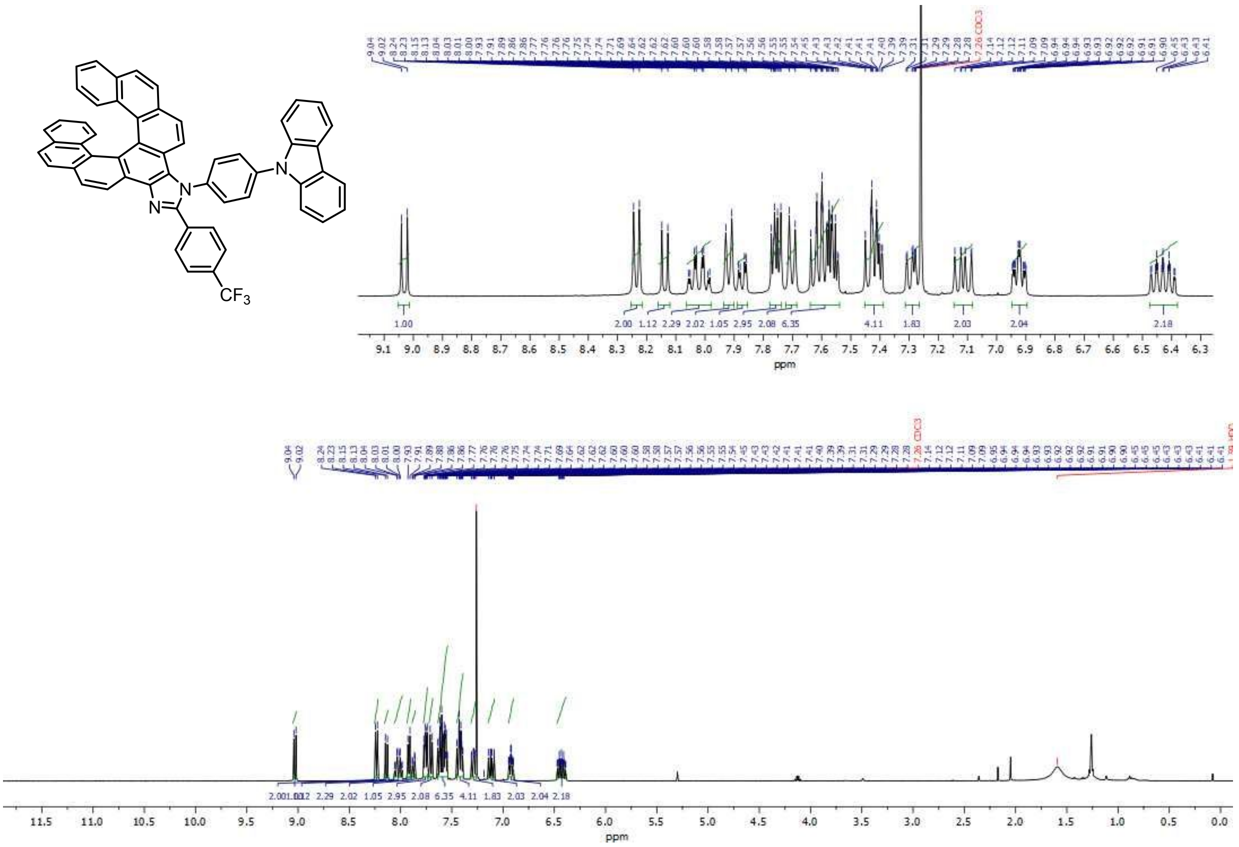

Figure S19 ^{1}H *NMR spectrum of* ***3-CF₃*** *(400 MHz, $CDCl_3$)*

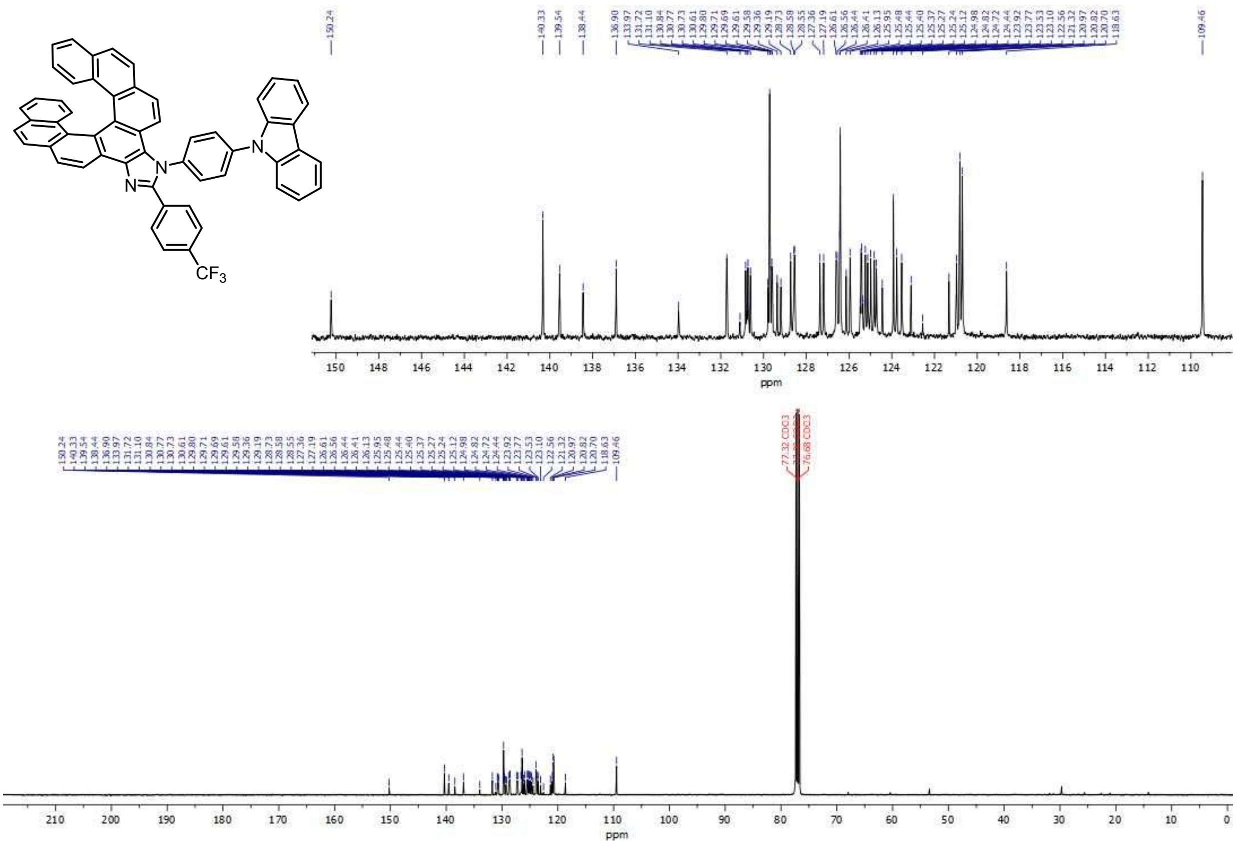

Figure S20 ^{13}C *{^{1}H} NMR spectrum of* ***3-CF₃*** *(101 MHz, $CDCl_3$)*

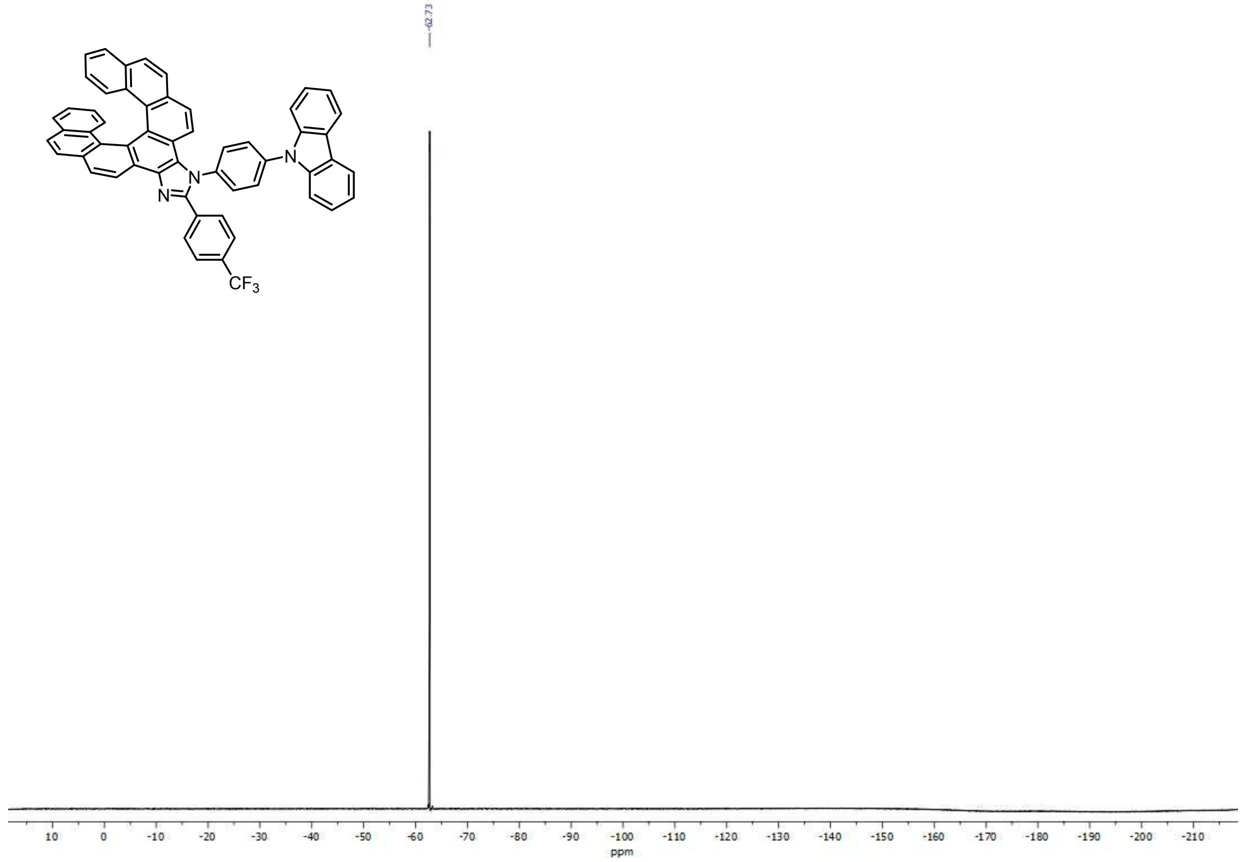

Figure S21 ^{19}*F {*^{1}*H} NMR spectrum of* ***3-CF₃*** *(376 MHz, CDCl*$_3$*)*

4. DFT Calculation Details of **1-3**

Optimizations of structures, calculations of HOMO/LUMO energies and TD-DFT calculations were performed with the package Gaussian09[5]. B3LYP[6,7] was used as a functional in combination with 6-311G(d,p)++ basis set for optimization and energy calculations. The vibrational analysis showed that all structures correspond to the local minima in the potential energy surface. Cartesian coordinates all optimized structures are following.

4.1 Cartesian coordinates of optimized structures of **1-H – 3-H, 1-CN – 3-CN and 1-CF$_3$ – 3-CF$_3$**

1-H

Total energy= -1764.21186178

Number of imaginary frequencies= 0

Zero-point correction= 0.557268 (Hartree/Particle)

Thermal correction to Energy= 0.589043

Thermal correction to Enthalpy= 0.589987

Thermal correction to Gibbs Free Energy= 0.493742

Sum of electronic and zero-point Energies= -1763.654594

Sum of electronic and thermal Energies= -1763.622819

Sum of electronic and thermal Enthalpies= -1763.621875

Sum of electronic and thermal Free Energies= -1763.718120

0 1

C	4.12468900	1.98859300	-1.34739400
C	3.98157400	3.28293900	-0.75899600
C	2.79591300	3.65862300	-0.21334900
C	1.70859900	2.73686100	-0.09834600
C	1.87356200	1.37846900	-0.47289500
C	3.05659000	1.04464500	-1.25707300
C	0.44950000	3.18871900	0.37583500
C	-0.63370800	2.35812200	0.37307000
C	-0.48405000	0.97312900	0.08083300
C	0.82790700	0.43791300	-0.12330100

C	-1.57336600	0.05251700	-0.02400700
C	-1.36886700	-1.29754900	-0.28088700
C	-0.06050000	-1.86667700	-0.26552400
C	1.03693900	-1.00840700	0.00011100
C	0.13038600	-3.25445200	-0.48848300
C	1.38611200	-3.78349500	-0.42326000
C	2.47301500	-3.00324700	0.06099700
C	2.27843600	-1.63979300	0.40612600
C	3.75560700	-3.61404600	0.22942200
C	4.78822900	-2.94202000	0.80129500
C	4.57599200	-1.64824700	1.36865500
C	3.30615900	-1.01022600	1.22465000
C	3.16143700	-0.12539200	-2.04854100
C	4.28821600	-0.39751800	-2.79576600
C	5.37873000	0.49091100	-2.79477500
C	5.28543600	1.67033200	-2.09123700
C	5.58749600	-1.03701500	2.14694700
C	5.34879800	0.13112000	2.83469700
C	4.06740000	0.70955500	2.78360400
C	3.07819700	0.15335300	2.00012200
N	-2.96239000	0.20626900	-0.00023100
C	-3.50133100	-1.05888300	-0.24368200
N	-2.55859300	-1.96206700	-0.41331500
C	-3.69790600	1.40735500	0.27390100
C	-4.26312300	1.59773400	1.53331400
C	-4.98913800	2.75837800	1.79476500
C	-5.14341800	3.72635500	0.80392500
C	-4.57123900	3.53296000	-0.45385300
C	-3.84997600	2.37265300	-0.72262900
C	-4.93524700	-1.39730700	-0.28180100
C	-5.30592700	-2.68410800	0.14394600
C	-6.63468200	-3.08947700	0.10592300
C	-7.62174000	-2.22163300	-0.36186200

C -7.26385000 -0.94808300 -0.79764200

C -5.93356000 -0.53615000 -0.75998500

H 4.81099300 3.97948800 -0.82040900

H 2.64995100 4.66998400 0.15167300

H 0.34282000 4.22691800 0.67166000

H -1.60619200 2.74840400 0.63183700

H -0.73257500 -3.85779900 -0.74079800

H 1.56235400 -4.82577600 -0.66756100

H 3.88394300 -4.63493000 -0.11522400

H 5.76464500 -3.40395900 0.90221500

H 2.33120000 -0.81418000 -2.09302000

H 4.32591900 -1.29984000 -3.39558700

H 6.26902400 0.26327400 -3.37018500

H 6.09407800 2.39337800 -2.12128000

H 6.55238900 -1.52858100 2.21787800

H 6.12817200 0.58264600 3.43825000

H 3.84944500 1.59420700 3.37136700

H 2.09736100 0.60433300 2.00549400

H -4.13410300 0.83891200 2.29561800

H -5.43120400 2.90557600 2.77335600

H -4.68837100 4.28375600 -1.22681000

H -3.40088300 2.20990800 -1.69544400

H -4.53449300 -3.35582100 0.49788700

H -6.90079400 -4.08500300 0.44321600

H -8.02073000 -0.27082000 -1.17725900

H -5.67990000 0.45020000 -1.12203300

H -8.65846300 -2.53746100 -0.39121700

H -5.70747000 4.62872600 1.01009000

1-CN

Total energy= -1856.47874091

Number of imaginary frequencies= 0

Zero-point correction= 0.555909 (Hartree/Particle)

Thermal correction to Energy= 0.589502

Thermal correction to Enthalpy= 0.590446

Thermal correction to Gibbs Free Energy= 0.489875

Sum of electronic and zero-point Energies= -1855.922832

Sum of electronic and thermal Energies= -1855.889239

Sum of electronic and thermal Enthalpies= -1855.888295

Sum of electronic and thermal Free Energies= -1855.988866

0 1

C 4.61618500 1.73879800 -1.40567500

C 4.57834400 3.04855600 -0.83520300

C 3.43192800 3.52037600 -0.28081000

C 2.28047000 2.68422200 -0.13810000

C 2.33866300 1.31177300 -0.49359600

C 3.48226200 0.87860100 -1.28748300

C 1.06554300 3.23642300 0.34416800

C -0.07672800 2.48933000 0.36825500

C -0.03319900 1.09275300 0.09866000

C 1.23150500 0.45773400 -0.11439400

C -1.18893700 0.25303200 0.01984700

C -1.08759200 -1.11364400 -0.21662000

C 0.17571100 -1.77891100 -0.20736500

C 1.33522900 -0.99902600 0.03199000

C 0.26147200 -3.17966100 -0.40982800

C 1.47659900 -3.79760200 -0.35190000

C 2.62378500 -3.09139500 0.10497300

C 2.53307500 -1.71241200 0.43179100

C 3.86207700 -3.79010400 0.26334600

C 4.94920700 -3.18440100 0.80727100

C 4.83942800 -1.87038200 1.35731800

C 3.61603300 -1.14561600 1.22425000

C 3.48809000 -0.30695300 -2.06202000

C 4.58032000 -0.67203600 -2.82088300

C 5.73358500 0.13274500 -2.84817300

C	5.73880000	1.32522100	-2.16074900
C	5.90533900	-1.32126100	2.10869900
C	5.76182600	-0.12870900	2.78093000
C	4.52393500	0.53853900	2.74208200
C	3.48408500	0.04244400	1.98427200
N	-2.56053200	0.51220700	0.05132100
C	-3.19503600	-0.71217300	-0.16524300
N	-2.32343400	-1.68746800	-0.32493900
C	-3.19520900	1.77583700	0.29970000
C	-3.70638800	2.05499900	1.56560300
C	-4.33009000	3.27914900	1.80134500
C	-4.43510700	4.22038200	0.77871900
C	-3.91675500	3.93701500	-0.48540500
C	-3.29758600	2.71378100	-0.72874500
C	-4.64583800	-0.95438600	-0.19763100
C	-5.08814700	-2.24427200	0.15047000
C	-6.43311300	-2.57165300	0.11931200
C	-7.38164400	-1.61191900	-0.26786800
C	-6.95190400	-0.32857200	-0.62909600
C	-5.60180400	-0.00585000	-0.59442000
H	5.45671400	3.67973800	-0.91812300
H	3.36694600	4.54503400	0.07002300
H	1.04061400	4.28414100	0.62392500
H	-1.01368000	2.95559400	0.63174200
H	-0.64569800	-3.72326700	-0.64169300
H	1.57305900	-4.85335100	-0.58197800
H	3.91169300	-4.82240200	-0.06697500
H	5.89203000	-3.71295500	0.89967100
H	2.60835300	-0.93255200	-2.08474400
H	4.54213400	-1.58285600	-3.40761700
H	6.59554800	-0.16838300	-3.43275400
H	6.59836100	1.98542300	-2.21278000
H	6.83414100	-1.87887400	2.17187400

H 6.58162200 0.27541400 3.36409500
H 4.37977700 1.44487800 3.31934400
H 2.53741300 0.56124200 1.99859700
H -3.61426800 1.31727300 2.35368500
H -4.73053900 3.49650100 2.78467500
H -3.99518500 4.66717400 -1.28250800
H -2.88906100 2.48188900 -1.70537500
H -4.35333500 -2.98287400 0.44164600
H -6.75891800 -3.56681000 0.39577600
H -7.67798900 0.41085600 -0.94359500
H -5.30082600 0.98606800 -0.89711700
C -8.77279000 -1.94056700 -0.29712500
N -9.89802700 -2.20478900 -0.31970200
H -4.91873100 5.17221100 0.96544000

1-CF_3

Total energy= -2101.36166295

Number of imaginary frequencies= 0

Zero-point correction= 0.561679 (Hartree/Particle)

Thermal correction to Energy= 0.597136

Thermal correction to Enthalpy= 0.598080

Thermal correction to Gibbs Free Energy= 0.492120

Sum of electronic and zero-point Energies= -2100.799984

Sum of electronic and thermal Energies= -2100.764527

Sum of electronic and thermal Enthalpies= -2100.763583

Sum of electronic and thermal Free Energies= -2100.869543

0 1
C 5.26948400 1.56920400 -1.40330100
C 5.30429600 2.87767400 -0.82976900
C 4.18530800 3.41149900 -0.27556600
C 2.98873200 2.64082200 -0.13630200
C 2.97080600 1.26793500 -0.49423900
C 4.08905000 0.77351900 -1.28828200

C	1.80551900	3.25863800	0.34545200
C	0.62359800	2.57595200	0.36656300
C	0.58974000	1.17943400	0.09500600
C	1.81768900	0.47557300	-0.11734600
C	-0.61043500	0.40476300	0.01707800
C	-0.58520500	-0.96518000	-0.21855700
C	0.63922400	-1.69901100	-0.21057100
C	1.84056100	-0.98448200	0.02779900
C	0.64754600	-3.10253000	-0.41349400
C	1.82669600	-3.78658000	-0.35724700
C	3.01168300	-3.14501100	0.09889000
C	2.99730300	-1.76340100	0.42657900
C	4.20929400	-3.91145300	0.25649000
C	5.32872800	-3.36725100	0.79994500
C	5.29180600	-2.04964800	1.35111700
C	4.11004700	-1.25836500	1.21967200
C	4.02934400	-0.40929300	-2.06484800
C	5.10010200	-0.83323500	-2.82360900
C	6.29670600	-0.09418400	-2.84784800
C	6.36811600	1.09477200	-2.15799400
C	6.38651500	-1.56125700	2.10303900
C	6.30912700	-0.36370700	2.77715600
C	5.10972500	0.37067100	2.73989400
C	4.04380500	-0.06634200	1.98191300
N	-1.96609400	0.73868600	0.05250000
C	-2.66707800	-0.44921800	-0.16066000
N	-1.85194000	-1.47080900	-0.32303800
C	-2.53214400	2.03403000	0.30229500
C	-3.03302000	2.33657200	1.56693300
C	-3.59028200	3.59199400	1.80460700
C	-3.63972100	4.54095600	0.78500800
C	-3.13273100	4.23369600	-0.47812600
C	-2.58021800	2.97936700	-0.72341600

C	-4.13102000	-0.60684400	-0.18022900
C	-4.64799200	-1.85444300	0.21067700
C	-6.01328900	-2.09696000	0.19576400
C	-6.89517900	-1.09637300	-0.21851200
C	-6.39789700	0.14128700	-0.62161100
C	-5.02841200	0.38350200	-0.60548400
H	6.21678300	3.45882200	-0.90992800
H	4.17724400	4.43734500	0.07776300
H	1.83823400	4.30560200	0.62729300
H	-0.28659700	3.09269800	0.62977300
H	-0.28868800	-3.59492700	-0.64425400
H	1.86432800	-4.84602400	-0.58763200
H	4.20120600	-4.94479400	-0.07430400
H	6.24098800	-3.94705400	0.89180100
H	3.11591800	-0.98444600	-2.08867200
H	5.01187600	-1.73946000	-3.41207200
H	7.14117100	-0.44210400	-3.43202700
H	7.26355900	1.70570100	-2.20770500
H	7.28326200	-2.16922400	2.16494400
H	7.15014300	-0.00606800	3.36050000
H	5.01569000	1.28264700	3.31863800
H	3.12692300	0.50352000	1.99773300
H	-2.98479900	1.59228700	2.35273800
H	-3.98268700	3.82724600	2.78706300
H	-3.16851700	4.96947800	-1.27313800
H	-2.18158100	2.72883100	-1.69953300
H	-3.95837300	-2.62836100	0.52034500
H	-6.39398100	-3.06358200	0.50131500
H	-7.07614500	0.91336300	-0.96237300
H	-4.66860600	1.34340700	-0.94553600
C	-8.37775500	-1.33675500	-0.17557500
F	-9.04775400	-0.58987400	-1.08275200
F	-8.90413700	-1.02789100	1.03971400

F -8.69575300 -2.62993100 -0.41116500

H -4.07135800 5.51721300 0.97310800

2-H

Total energy= -2281.78649157

Number of imaginary frequencies= 0

Zero-point correction= 0.734021 (Hartree/Particle)

Thermal correction to Energy= 0.776510

Thermal correction to Enthalpy= 0.777454

Thermal correction to Gibbs Free Energy= 0.654982

Sum of electronic and zero-point Energies= -2281.052471

Sum of electronic and thermal Energies= -2281.009981

Sum of electronic and thermal Enthalpies= -2281.009037

Sum of electronic and thermal Free Energies= -2281.131510

0 1

C -4.96080400 -3.00910700 -1.16602200

C -4.20202400 -4.11486500 -0.67238800

C -2.91643000 -3.93703600 -0.27224700

C -2.33327200 -2.63269600 -0.21163500

C -3.11558700 -1.48088600 -0.48394900

C -4.40708700 -1.69315800 -1.12682500

C -0.95689800 -2.49050100 0.10385800

C -0.35245300 -1.26801900 0.04521300

C -1.12429200 -0.08671600 -0.14051500

C -2.55203300 -0.18037300 -0.18205100

C -0.56343000 1.21984100 -0.29261600

C -1.36255300 2.34551300 -0.45012600

C -2.77725800 2.28203400 -0.27439000

C -3.35416800 1.02638100 0.04601100

C -3.57707800 3.44731800 -0.39512000

C -4.92155900 3.37094200 -0.17696800

C -5.49877100 2.18731600 0.36189900

C -4.69244600 1.04420800 0.60563500

C	-6.89173400	2.17030100	0.68732100
C	-7.45804200	1.10560000	1.31231400
C	-6.64183500	0.03006600	1.77909700
C	-5.24540500	0.01744700	1.47895200
C	-5.09924400	-0.68062100	-1.83525300
C	-6.30858800	-0.92384700	-2.45210100
C	-6.89668800	-2.20029300	-2.39428400
C	-6.22102000	-3.22535900	-1.77183100
C	-7.19288600	-0.97334300	2.61098800
C	-6.39452500	-1.92637600	3.20156600
C	-5.00353000	-1.88183500	2.99577400
C	-4.44890200	-0.93650600	2.15877400
N	0.74714800	1.68842800	-0.41515000
C	0.65036600	3.06281700	-0.63722500
N	-0.60315000	3.46570400	-0.66053100
C	1.95830400	0.93697900	-0.25274900
C	2.65386400	0.98362700	0.95395000
C	3.83031900	0.26216800	1.11650400
C	4.34538900	-0.51626700	0.06694900
C	3.63497800	-0.55574400	-1.14598800
C	2.45439400	0.15899300	-1.29921100
C	1.78320000	3.99195000	-0.79669800
C	1.62553300	5.29799200	-0.30420200
C	2.63825300	6.23886200	-0.45047800
C	3.82652500	5.89827400	-1.09707200
C	3.98774100	4.60972100	-1.60128500
C	2.97729000	3.66218500	-1.45396800
H	-4.64685900	-5.10414800	-0.69214600
H	-2.30351600	-4.78528600	0.01477100
H	-0.37495600	-3.38027300	0.31975400
H	0.71605500	-1.19072700	0.17700900
H	-3.09702600	4.37091000	-0.69325700
H	-5.55965400	4.23314300	-0.33988100

H	-7.48916000	3.03515400	0.41791400
H	-8.52024200	1.08987700	1.53241900
H	-4.66124900	0.30244300	-1.92113000
H	-6.80218900	-0.12438100	-2.99304100
H	-7.85642900	-2.38110500	-2.86521900
H	-6.63268700	-4.22941300	-1.76412000
H	-8.26104000	-0.95684200	2.80200900
H	-6.82712500	-2.68253900	3.84709100
H	-4.35823000	-2.58805200	3.50607000
H	-3.37566500	-0.90950200	2.04492900
H	2.26549800	1.57872600	1.77205600
H	4.35474800	0.30080700	2.06259200
H	4.01421600	-1.14569800	-1.97050000
H	1.91693300	0.12258100	-2.23992300
H	0.69714200	5.55895600	0.18753500
H	2.49969400	7.24061400	-0.05932600
H	4.90003600	4.34000200	-2.12177300
H	3.11745500	2.67428300	-1.86975300
N	5.54697000	-1.23879000	0.22486500
C	6.60367100	-0.72190900	1.03050000
C	7.27741500	-1.55834300	1.92928000
C	6.99184300	0.62008400	0.92597400
C	8.32227100	-1.06065600	2.70286100
C	8.02656300	1.11402700	1.71510600
H	6.47984400	1.27284900	0.22926200
C	8.70035200	0.27772900	2.60463900
H	8.83387200	-1.72084400	3.39432300
H	8.31528700	2.15513600	1.62301500
H	9.50974600	0.66430200	3.21274200
C	5.72859200	-2.49192900	-0.43100100
C	4.72674700	-3.46989300	-0.38931300
C	6.91917200	-2.76386500	-1.11629800
C	4.90989500	-4.68991000	-1.03389800

H 3.80770600 -3.26991100 0.14847200

C 7.10159900 -3.99267600 -1.74456800

C 6.09811800 -4.96015600 -1.71192700

H 4.12546300 -5.43737100 -0.99298000

H 8.02853700 -4.18872400 -2.27187700

H 6.24070500 -5.91346500 -2.20715600

H 4.61577900 6.63264000 -1.21259800

H 6.98146900 -2.59696500 2.01508700

H 7.69796900 -2.01153800 -1.15045900

2-CN

Total energy= -2374.05354021

Number of imaginary frequencies= 0

Zero-point correction= 0.732551 (Hartree/Particle)

Thermal correction to Energy= 0.776932

Thermal correction to Enthalpy= 0.777876

Thermal correction to Gibbs Free Energy= 0.650315

Sum of electronic and zero-point Energies= -2373.320989

Sum of electronic and thermal Energies= -2373.276608

Sum of electronic and thermal Enthalpies= -2373.275664

Sum of electronic and thermal Free Energies= -2373.403225

0 1

C -5.22110200 -2.64801200 -1.87136000

C -4.53625900 -3.86956800 -1.58650100

C -3.25707700 -3.84651800 -1.13110900

C -2.60992800 -2.61366800 -0.80409500

C -3.32328600 -1.38853800 -0.86455000

C -4.60207000 -1.39806900 -1.56511800

C -1.23966700 -2.61327800 -0.43584700

C -0.57254400 -1.43887600 -0.23653200

C -1.27752600 -0.20354800 -0.20240000

C -2.70538100 -0.20671800 -0.29940600

C -0.64918200 1.07667400 -0.08389600

C	-1.38894400	2.25384600	-0.04498500
C	-2.81129700	2.23590300	0.08032500
C	-3.45606400	0.97501100	0.14143300
C	-3.54985200	3.44301100	0.16825800
C	-4.90283800	3.39663600	0.33934500
C	-5.55171300	2.16402900	0.62763200
C	-4.80842700	0.95444000	0.66377700
C	-6.95364200	2.15809800	0.91267200
C	-7.58909400	1.02378700	1.30533800
C	-6.84034500	-0.16371900	1.57097300
C	-5.43681400	-0.19144200	1.30763100
C	-5.21739500	-0.23006700	-2.07785300
C	-6.41694000	-0.28060800	-2.75665600
C	-7.07153700	-1.50859700	-2.96106700
C	-6.47045200	-2.67132100	-2.53493600
C	-7.46568400	-1.27904100	2.17691100
C	-6.73302300	-2.36846800	2.59043500
C	-5.33518700	-2.35717900	2.43253100
C	-4.70891200	-1.29850500	1.80892700
N	0.68490100	1.48719600	-0.07824600
C	0.66225600	2.88160700	-0.03453800
N	-0.57075300	3.34801000	-0.01451700
C	1.84764200	0.64664600	-0.02737200
C	2.48257800	0.40562100	1.18972700
C	3.61646500	-0.39533300	1.24499300
C	4.13255600	-0.99098600	0.08116000
C	3.47668600	-0.74765000	-1.13980300
C	2.35388100	0.06734600	-1.19165800
C	1.82908100	3.77627400	0.01025200
C	1.64797500	5.03924000	0.60427800
C	2.68277400	5.95743700	0.66259000
C	3.93767500	5.63791300	0.12057800
C	4.12586700	4.38859000	-0.48457300

C	3.08507500	3.47116700	-0.53802500
H	-5.03044700	-4.80930700	-1.80892800
H	-2.69848200	-4.76775800	-1.00216500
H	-0.71044200	-3.55905700	-0.38913300
H	0.49356300	-1.44960600	-0.06926700
H	-3.01863700	4.38062200	0.06342600
H	-5.49393000	4.30632400	0.33165200
H	-7.50071000	3.08878500	0.80389500
H	-8.65724000	1.02173300	1.49481100
H	-4.72767300	0.72501100	-1.96042000
H	-6.85089700	0.63477500	-3.14279100
H	-8.02307500	-1.54027800	-3.47972600
H	-6.93352800	-3.63278900	-2.73158300
H	-8.53779800	-1.24424500	2.34111700
H	-7.22221200	-3.21145100	3.06538700
H	-4.74125400	-3.18061500	2.81295400
H	-3.63206600	-1.30589100	1.73112300
H	2.09546200	0.85979400	2.09434700
H	4.10717800	-0.56246000	2.19510700
H	3.85024500	-1.20234500	-2.04822800
H	1.85360200	0.24123500	-2.13751600
H	0.67725200	5.28403400	1.01421500
H	2.52957400	6.92333800	1.12793800
H	5.08762700	4.14405300	-0.91838200
H	3.25498000	2.52309000	-1.02606100
C	5.01322700	6.57782200	0.18078800
N	5.88498900	7.33566300	0.22938200
N	5.27546300	-1.81181400	0.13402800
C	6.19048100	-1.85346700	-0.96066400
C	6.64141900	-3.08317800	-1.45467100
C	6.66027800	-0.66926900	-1.54178000
C	7.54904400	-3.12386500	-2.50955400
C	7.55552900	-0.71796200	-2.60660500

H 6.32501300 0.28511400 -1.15355200

C 8.00705700 -1.94393200 -3.09424500

H 7.89038800 -4.08313400 -2.88219900

H 7.91340300 0.20658800 -3.04567000

H 8.70938200 -1.97879800 -3.91877000

C 5.54469700 -2.61271400 1.28400900

C 6.82892800 -2.63496500 1.84097500

C 4.53686800 -3.39720200 1.85865900

C 7.09748500 -3.43065100 2.95134700

H 7.61251900 -2.03131800 1.39903300

C 4.80901800 -4.17769900 2.97857700

C 6.08993600 -4.20161500 3.52942500

H 8.09660000 -3.43764000 3.37236500

H 4.01918300 -4.78038200 3.41286800

H 6.30037900 -4.81532500 4.39741600

H 6.28245800 -4.00179800 -1.00615400

H 3.54360600 -3.39292100 1.42587500

2-CF_3

Total energy= -2618.93663345

Number of imaginary frequencies= 0

Zero-point correction= 0.738413 (Hartree/Particle)

Thermal correction to Energy= 0.784619

Thermal correction to Enthalpy= 0.785563

Thermal correction to Gibbs Free Energy= 0.653363

Sum of electronic and zero-point Energies= -2618.198221

Sum of electronic and thermal Energies= -2618.152015

Sum of electronic and thermal Enthalpies= -2618.151070

Sum of electronic and thermal Free Energies= -2618.283271

0 1

C -5.87069800 -2.46168400 -1.39113000

C -5.33045100 -3.71709600 -0.97389300

C -4.04378800 -3.79984200 -0.54750200

C	-3.24092000	-2.62745900	-0.38519800
C	-3.80095400	-1.33830100	-0.58000800
C	-5.09551900	-1.27070600	-1.24755300
C	-1.86837600	-2.75599000	-0.04893000
C	-1.05502700	-1.66024500	-0.01352800
C	-1.60150300	-0.35063300	-0.11712000
C	-3.02157100	-0.18449800	-0.17902600
C	-0.81572900	0.84380900	-0.17066600
C	-1.40141100	2.10167100	-0.25864500
C	-2.80889000	2.27960100	-0.09847000
C	-3.60228600	1.12713900	0.13117700
C	-3.38821600	3.57312400	-0.14064300
C	-4.72849500	3.72074100	0.06790500
C	-5.51329700	2.62396100	0.52050300
C	-4.92513500	1.34177600	0.68539900
C	-6.89242500	2.83172400	0.83889500
C	-7.64779600	1.84283200	1.38317100
C	-7.04272900	0.60863300	1.77259700
C	-5.66520500	0.36998000	1.47947700
C	-5.58448300	-0.10215100	-1.88118200
C	-6.80411700	-0.08192500	-2.52474200
C	-7.60736100	-1.23566100	-2.57067100
C	-7.13544300	-2.40700600	-2.02300000
C	-7.77779500	-0.33960400	2.52273500
C	-7.17148000	-1.45996700	3.04401400
C	-5.79058300	-1.64708400	2.85147300
C	-5.06212800	-0.75645500	2.09122900
N	0.55838300	1.07849600	-0.24609400
C	0.71047600	2.45942900	-0.37246000
N	-0.45262200	3.07894800	-0.38135300
C	1.61011500	0.10924800	-0.12411700
C	2.25112700	-0.07619400	1.09915500
C	3.27227100	-1.01006600	1.22389400

C	3.68476500	-1.77571300	0.11979400
C	3.03040300	-1.57805000	-1.11028600
C	2.00278700	-0.65147900	-1.22590400
C	1.98432300	3.19334700	-0.45614700
C	1.99520600	4.51837200	0.01437900
C	3.14492400	5.29055400	-0.05314100
C	4.31343600	4.75578600	-0.60070100
C	4.31574000	3.44889000	-1.08334900
C	3.16207900	2.67485700	-1.01379500
H	-5.94248200	-4.60742200	-1.07168800
H	-3.59734000	-4.76169400	-0.31741400
H	-1.45845800	-3.74841000	0.10564200
H	0.00730700	-1.78528800	0.13019800
H	-2.74901600	4.41588500	-0.37189300
H	-5.20199000	4.69123500	-0.03626900
H	-7.32317600	3.80551800	0.63039200
H	-8.69943100	2.00027800	1.59802700
H	-4.98000100	0.79232900	-1.88701700
H	-7.13856500	0.82979300	-3.00662700
H	-8.57319400	-1.20929900	-3.06254000
H	-7.71694900	-3.32033000	-2.09552900
H	-8.82983800	-0.14840900	2.70791500
H	-7.74316500	-2.17308500	3.62724400
H	-5.28946800	-2.49137700	3.31144100
H	-3.99863000	-0.91143300	1.98793800
H	1.93994900	0.50433800	1.95972600
H	3.75378100	-1.14985800	2.18294300
H	3.33311900	-2.15186600	-1.97661900
H	1.50758000	-0.50788400	-2.17953000
H	1.08379900	4.92939600	0.42763800
H	3.13416600	6.30908300	0.31483500
H	5.21290900	3.03767600	-1.52860800
H	3.18550400	1.67235600	-1.41439800

N	4.72771500	-2.71419000	0.24092400
C	5.79861700	-2.49497100	1.15939300
C	6.21353200	-3.52304800	2.01403500
C	6.45486100	-1.25883000	1.20769400
C	7.26904200	-3.31611400	2.89810000
C	7.49857000	-1.05413900	2.10558500
H	6.14393500	-0.46143700	0.54336100
C	7.91380700	-2.08117900	2.95267300
H	7.57982500	-4.12116600	3.55452700
H	7.99767400	-0.09198700	2.13202400
H	8.73141600	-1.92091400	3.64561600
C	4.73070700	-3.90567200	-0.54389800
C	3.57531600	-4.68919900	-0.65607100
C	5.89900900	-4.31413800	-1.19857600
C	3.58807800	-5.85155700	-1.42171900
H	2.67126200	-4.38495100	-0.14232100
C	5.90856300	-5.48671300	-1.94896700
C	4.75388700	-6.25871600	-2.06959200
H	2.68625800	-6.44848400	-1.49982000
H	6.82057400	-5.79073800	-2.45034900
H	4.76248900	-7.16758700	-2.65971000
C	5.57476400	5.57134400	-0.61685800
F	6.43131200	5.17957100	-1.58791900
F	6.25518000	5.47282100	0.55705400
F	5.33293400	6.88859100	-0.80639700
H	5.70922600	-4.48133500	1.97957200
H	6.79620300	-3.71293700	-1.11241800

3-H

Total energy= -2280.60168648

Number of imaginary frequencies= 0

Zero-point correction= 0.712918 (Hartree/Particle)

Thermal correction to Energy= 0.754157

Thermal correction to Enthalpy= 0.755101

Thermal correction to Gibbs Free Energy= 0.636175

Sum of electronic and zero-point Energies= -2279.888769

Sum of electronic and thermal Energies= -2279.847530

Sum of electronic and thermal Enthalpies= -2279.846586

Sum of electronic and thermal Free Energies= -2279.965512

0 1

C 4.83132400 -2.82159100 1.90829700

C 4.05006100 -3.98225900 1.61797500

C 2.78707400 -3.85317700 1.13565000

C 2.25484300 -2.57210900 0.78822900

C 3.06859800 -1.41182500 0.85554600

C 4.32916100 -1.52556700 1.57939200

C 0.89619900 -2.45731700 0.39402900

C 0.33489200 -1.23107800 0.18323400

C 1.14086600 -0.05817400 0.16305800

C 2.56235800 -0.18317900 0.27718700

C 0.62468700 1.26915800 0.03243400

C 1.46001700 2.37821700 -0.01453700

C 2.87565900 2.23794600 -0.12411400

C 3.41485200 0.92671000 -0.16093600

C 3.71320500 3.37917700 -0.21333900

C 5.05996000 3.21952400 -0.36063200

C 5.60938800 1.93388200 -0.62477800

C 4.76951200 0.78944700 -0.66147900

C 7.01031100 1.80956400 -0.88636600

C 7.55625000 0.62300800 -1.25897100

C 6.71615100 -0.50096900 -1.52689300

C 5.31113500 -0.41019900 -1.28588600

C 5.03484600 -0.41037700 2.09416300

C 6.21176500 -0.55849200 2.79760400

C 6.75239200 -1.83678900 3.02624500

C 6.06057700 -2.94680500 2.59752000

C	7.25665300	-1.66984000	-2.11339200
C	6.44329100	-2.69925800	-2.52941500
C	5.04879100	-2.57121400	-2.39420400
C	4.50240300	-1.45853200	-1.78987400
N	-0.67086700	1.79607300	0.01358500
C	-0.52838100	3.18483500	-0.04366500
N	0.73804200	3.54124300	-0.06024000
C	-1.90455900	1.06775200	-0.01687500
C	-2.60101600	0.93165200	-1.21625100
C	-3.80745500	0.23910100	-1.24626100
C	-4.31465500	-0.34209100	-0.08048900
C	-3.60628000	-0.21187600	1.11952200
C	-2.41280200	0.50027300	1.15243700
C	-1.62793600	4.16373600	-0.10703500
C	-1.40231000	5.34531400	-0.83286700
C	-2.37996500	6.33050400	-0.90615100
C	-3.60080700	6.16011900	-0.25293000
C	-3.83010700	4.99815600	0.48021400
C	-2.85418400	4.00699900	0.55518000
H	4.45711000	-4.95902900	1.85680700
H	2.15394200	-4.72394800	1.00072900
H	0.28971000	-3.35484000	0.33614200
H	-0.72474800	-1.15533800	-0.00739500
H	3.25870000	4.35810700	-0.12713200
H	5.72419300	4.07726700	-0.35263500
H	7.63041700	2.69316800	-0.77646200
H	8.62348200	0.53117200	-1.43071800
H	4.63238200	0.58243900	1.95977900
H	6.71649800	0.31944100	3.18472600
H	7.68705000	-1.94599000	3.56473600
H	6.43404600	-3.94302900	2.81115300
H	8.33051900	-1.72496800	-2.25978900
H	6.86848000	-3.58448700	-2.98886600

H	4.39484600	-3.34669700	-2.77700300
H	3.42757900	-1.37657400	-1.72968900
H	-2.20492500	1.38148700	-2.11856700
H	-4.36345500	0.15394300	-2.17171700
H	-3.98924900	-0.67617400	2.01993000
H	-1.86088800	0.60185200	2.07945200
H	-0.44932400	5.47624400	-1.32926700
H	-2.18901600	7.23342200	-1.47522100
H	-4.76961600	4.86303300	1.00427600
H	-3.04653000	3.12381100	1.14827600
N	-5.53734200	-1.05985100	-0.11395900
C	-6.66456300	-0.79563400	0.67384000
C	-7.67990500	-1.72744200	0.34805300
C	-6.87325700	0.21175700	1.61676400
C	-8.91630400	-1.65384600	0.99631000
C	-8.11278700	0.26287200	2.24760000
H	-6.10348400	0.93638300	1.85019400
C	-9.12518300	-0.66101400	1.94648400
H	-9.70416400	-2.35980600	0.75817200
H	-8.29840300	1.03577800	2.98482300
H	-10.07879900	-0.59422100	2.45686600
C	-5.82243400	-2.15356200	-0.94125000
C	-5.00559500	-2.80406600	-1.86720300
C	-7.14263800	-2.59388500	-0.67961900
C	-5.53746300	-3.89639100	-2.54602900
H	-3.98978000	-2.47895700	-2.05317800
C	-7.65259000	-3.69395200	-1.37536500
C	-6.84827400	-4.33766500	-2.30851300
H	-4.92254400	-4.41696500	-3.27139500
H	-8.66155000	-4.04357200	-1.18676000
H	-7.23239500	-5.19071900	-2.85538100
H	-4.36355400	6.92836300	-0.31046000

3-CN

Total energy= -2372.43699700

Number of imaginary frequencies= 0

Zero-point correction= 0.714056 (Hartree/Particle)

Thermal correction to Energy= 0.757066

Thermal correction to Enthalpy= 0.758010

Thermal correction to Gibbs Free Energy= 0.635052

Sum of electronic and zero-point Energies= -2371.722941

Sum of electronic and thermal Energies= -2371.679931

Sum of electronic and thermal Enthalpies= -2371.678987

Sum of electronic and thermal Free Energies= -2371.801945

0 1

C 5.17505100 -2.72982300 1.88456900

C 4.46849600 -3.93929400 1.58988600

C 3.19230300 -3.89035100 1.11661200

C 2.57379100 -2.64192700 0.78337600

C 3.31169100 -1.42817700 0.85640200

C 4.58439100 -1.46435400 1.57146800

C 1.20686300 -2.61493300 0.39472800

C 0.56300900 -1.42405300 0.19094900

C 1.29312300 -0.20011900 0.17284600

C 2.72263500 -0.23117800 0.28573400

C 0.69264900 1.09614000 0.05545400

C 1.45759900 2.26000400 0.02110200

C 2.88258200 2.21402800 -0.09106100

C 3.50276700 0.93722400 -0.14791600

C 3.64610700 3.40870800 -0.17047400

C 5.00331900 3.33428900 -0.32824700

C 5.63035500 2.08673300 -0.61272300

C 4.86160000 0.88951900 -0.65854800

C 7.03700600 2.05381200 -0.88403200

C 7.65520400 0.90410900 -1.27316600

C	6.88403400	-0.26979300	-1.54754700
C	5.47468200	-0.27028700	-1.29724800
C	5.22123400	-0.30736800	2.09077900
C	6.41613300	-0.38351600	2.78296900
C	7.04372500	-1.62785200	2.99449500
C	6.41958500	-2.78026200	2.56127200
C	7.49577600	-1.39986600	-2.14630700
C	6.74548100	-2.47955000	-2.56642300
C	5.34357800	-2.44137200	-2.42184500
C	4.72973600	-1.36667100	-1.80487500
N	-0.63415500	1.53685400	0.04213900
C	-0.58343300	2.93352400	-0.00181800
N	0.66249300	3.37312000	-0.01365100
C	-1.81169700	0.71918300	0.00548000
C	-2.46585300	0.49602800	-1.20863600
C	-3.62056500	-0.28549600	-1.24486800
C	-4.11484900	-0.86940900	-0.06972800
C	-3.44634800	-0.65107800	1.14509900
C	-2.30608600	0.14907600	1.18358400
C	-1.73046100	3.85571200	-0.05253800
C	-1.50476600	5.12727600	-0.62053400
C	-2.51736600	6.07443900	-0.68472000
C	-3.79523600	5.77534900	-0.17692700
C	-4.02939500	4.51702100	0.40042000
C	-3.00926000	3.57193000	0.46229600
H	4.94176100	-4.89058100	1.81765900
H	2.61595700	-4.80142200	0.97884100
H	0.65924200	-3.55152000	0.33519200
H	-0.50187800	-1.41720100	0.00566600
H	3.13203800	4.35831500	-0.06952400
H	5.61388300	4.23313300	-0.31324700
H	7.60226300	2.97488600	-0.76839500
H	8.72674600	0.88098000	-1.45244100

H	4.75248500	0.65983800	1.96984200
H	6.86669900	0.52469700	3.17229300
H	7.99127000	-1.68045800	3.52258000
H	6.86033900	-3.75339100	2.76214000
H	8.57185000	-1.38495000	-2.29969700
H	7.22477200	-3.33408600	-3.03503100
H	4.73620500	-3.25588600	-2.80563500
H	3.65028200	-1.35386500	-1.73899100
H	-2.07873600	0.94667300	-2.11687700
H	-4.14649600	-0.43838500	-2.18120700
H	-3.81560200	-1.11852000	2.05170200
H	-1.78454500	0.31753600	2.12061100
H	-0.51781600	5.35540500	-1.00531500
H	-2.32915700	7.04622700	-1.12869700
H	-5.00886200	4.28605200	0.80562300
H	-3.21565400	2.61995400	0.93210100
C	-4.84794100	6.74650400	-0.24372300
N	-5.70326200	7.53465800	-0.29800500
N	-5.27799700	-1.67676600	-0.10843200
C	-6.43216800	-1.49288000	0.66785800
C	-7.37085900	-2.50425800	0.34067400
C	-6.72781500	-0.49251000	1.59933700
C	-8.61918300	-2.51981000	0.97607000
C	-7.97795900	-0.52973800	2.21783000
H	-6.01787700	0.29298800	1.83389500
C	-8.91521800	-1.53398800	1.91545200
H	-9.34897300	-3.28757000	0.73568100
H	-8.23063300	0.23612300	2.94505700
H	-9.87887600	-1.53545100	2.41521200
C	-5.47062600	-2.79877900	-0.92867800
C	-4.59428400	-3.39453700	-1.84139000
C	-6.75761600	-3.33727100	-0.67416900
C	-5.03498700	-4.53392400	-2.51547500

H -3.60241300 -2.99464800 -2.02140200

C -7.17641700 -4.48194200 -1.36450100

C -6.31381400 -5.07282200 -2.28572300

H -4.37297400 -5.01334500 -3.23046300

H -8.15944600 -4.90580400 -1.18067800

H -6.62722400 -5.95932600 -2.82825700

3-CF_3

Total energy= -2617.75127021

Number of imaginary frequencies= 0

Zero-point correction= 0.717487 (Hartree/Particle)

Thermal correction to Energy= 0.762354

Thermal correction to Enthalpy= 0.763299

Thermal correction to Gibbs Free Energy= 0.635515

Sum of electronic and zero-point Energies= -2617.033784

Sum of electronic and thermal Energies= -2616.988916

Sum of electronic and thermal Enthalpies= -2616.987972

Sum of electronic and thermal Free Energies= -2617.115756

0 1

C 5.73359200 -2.48888800 1.83083800

C 5.15841400 -3.75944700 1.51926200

C 3.88345000 -3.83873600 1.05858800

C 3.13273500 -2.66044400 0.75318900

C 3.73619500 -1.37914800 0.84078600

C 5.00955100 -1.29141700 1.54570200

C 1.76906100 -2.77274600 0.37792200

C 1.00257700 -1.65714500 0.19986900

C 1.59628400 -0.36399400 0.19452500

C 3.01899900 -0.24438300 0.29596300

C 0.85895300 0.85841100 0.09854600

C 1.49151100 2.09594300 0.08023200

C 2.90981300 2.20463600 -0.04096400

C 3.66353400 1.00648000 -0.12161400

C	3.53848800	3.47395100	-0.10523700
C	4.89046900	3.55049900	-0.27202100
C	5.64647800	2.38547400	-0.57967100
C	5.01382000	1.11546200	-0.63971200
C	7.04373900	2.50964700	-0.86126100
C	7.77755000	1.44389200	-1.27369700
C	7.13744500	0.20000400	-1.56374800
C	5.74225800	0.04178000	-1.30251500
C	5.52137200	-0.08445000	2.08143300
C	6.71951400	-0.04423700	2.76346500
C	7.47646200	-1.21513100	2.94854200
C	6.97863000	-2.41707500	2.49892900
C	7.85981800	-0.84291500	-2.19038700
C	7.22764700	-1.98589900	-2.62449400
C	5.83473600	-2.10364400	-2.46640400
C	5.11656400	-1.11720900	-1.82370000
N	-0.50722300	1.15308100	0.09423300
C	-0.60677400	2.54589400	0.07182000
N	0.58015100	3.11586100	0.06427500
C	-1.59267700	0.21748400	0.04561300
C	-2.23642600	-0.03722900	-1.16371900
C	-3.29918500	-0.93420200	-1.21138300
C	-3.71034000	-1.60125100	-0.05302500
C	-3.05224400	-1.34967200	1.15689300
C	-2.00618200	-0.43566200	1.20746900
C	-1.85094700	3.33214400	0.03009300
C	-1.78822200	4.60103000	-0.57337000
C	-2.90557600	5.41950800	-0.62941700
C	-4.11516000	4.98905400	-0.07840700
C	-4.19146500	3.73984200	0.53259700
C	-3.06869700	2.91999100	0.58969000
H	5.73193000	-4.65689000	1.72542400
H	3.40729000	-4.80213900	0.90899800

H	1.32435000	-3.75966000	0.30798900
H	-0.05739000	-1.76127500	0.02601200
H	2.92597600	4.35879700	0.01352000
H	5.39873300	4.50854900	-0.24650500
H	7.50565500	3.48307300	-0.73359400
H	8.84174500	1.54034000	-1.46088400
H	4.95161200	0.82699500	1.97958900
H	7.07249400	0.89782100	3.16750200
H	8.42583100	-1.17455000	3.47052600
H	7.52201100	-3.33872200	2.67995100
H	8.92426800	-0.70879300	-2.35321500
H	7.78998800	-2.77237700	-3.11509200
H	5.31689100	-2.97029200	-2.86169100
H	4.04509700	-1.22355800	-1.74482700
H	-1.91419500	0.47812100	-2.06050000
H	-3.81834400	-1.11351900	-2.14460800
H	-3.35611900	-1.87981800	2.05091600
H	-1.49240000	-0.24198000	2.14169500
H	-0.84613900	4.93203800	-0.98956300
H	-2.83836900	6.39418600	-1.09697100
H	-5.12169700	3.41019100	0.97737300
H	-3.14918000	1.96710300	1.09167300
N	-4.78042700	-2.52795100	-0.10349400
C	-5.93203100	-2.49633500	0.69494500
C	-6.75915900	-3.59175500	0.34803300
C	-6.31935200	-1.56734500	1.66166000
C	-7.98400400	-3.76433400	0.99910300
C	-7.54350100	-1.76168500	2.29481900
H	-5.69781700	-0.71690400	1.91147500
C	-8.36826200	-2.85021700	1.97307500
H	-8.62893100	-4.59803400	0.74470200
H	-7.86540800	-1.05333700	3.04967900
H	-9.31505100	-2.97307300	2.48564000

C	-4.86483300	-3.63603400	-0.95795600
C	-3.94903600	-4.10335900	-1.90160700
C	-6.07857200	-4.31870000	-0.70275600
C	-4.27588600	-5.25855600	-2.60583600
H	-3.01120800	-3.59366300	-2.08247100
C	-6.38268900	-5.47695600	-1.42372000
C	-5.48113000	-5.93891600	-2.37542000
H	-3.58103300	-5.63976000	-3.34549500
H	-7.30836300	-6.01103200	-1.24045500
H	-5.70605300	-6.83471900	-2.94220100
C	-5.33605800	5.85812900	-0.19151300
F	-6.27118800	5.55874900	0.73772600
F	-5.93898000	5.72721200	-1.40260500
F	-5.04218300	7.17171900	-0.05289300

7hel

Total energy= -1154.34397505 a.u.

Number of imaginary frequencies= 0

Zero-point correction= 0.379098 (Hartree/Particle)

Thermal correction to Energy= 0.399237

Thermal correction to Enthalpy= 0.400182

Thermal correction to Gibbs Free Energy= 0.331804

Sum of electronic and zero-point Energies= -1153.964877

Sum of electronic and thermal Energies= -1153.944738

Sum of electronic and thermal Enthalpies= -1153.943793

Sum of electronic and thermal Free Energies= -1154.012171

C	-1.85730700	2.07016400	1.03978800
C	-1.84896400	3.28687100	0.29220900
C	-0.70866000	3.71038800	-0.31339000
C	0.46788500	2.89795000	-0.33006200
C	0.44041500	1.58739400	0.21200800
C	-0.69678300	1.23743100	1.05094000
C	1.67784100	3.42904500	-0.86229400

C	2.83464500	2.71394600	-0.78542400
C	2.81825300	1.35702700	-0.35339900
C	1.58170000	0.72546700	-0.03532200
C	4.04141900	0.63802700	-0.22379900
C	4.03981900	-0.64798600	0.22377200
C	2.81487200	-1.36394800	0.35336300
C	1.57989800	-0.72933100	0.03524000
C	2.82787900	-2.72087700	0.78547200
C	1.66929000	-3.43306900	0.86243100
C	0.46066100	-2.89899000	0.33017300
C	0.43648100	-1.58843800	-0.21206000
C	-0.71793300	-3.70845400	0.31366300
C	-1.85719400	-3.28212500	-0.29192200
C	-1.86248000	-2.06552000	-1.03969300
C	-0.69984100	-1.23574200	-1.05104400
C	-2.98058400	-1.72300200	-1.83650800
C	-0.67644500	-0.17025400	-1.98435500
C	-1.76443400	0.12064300	-2.77994800
C	-2.94415400	-0.64012600	-2.68514000
C	-2.97624100	1.73034600	1.83659600
C	-0.67607000	0.17168300	1.98401600
C	-1.76475300	-0.11658400	2.77961600
C	-2.94252600	0.64722400	2.68502800
H	-2.74510100	3.89819000	0.27826400
H	-0.66865600	4.67928800	-0.80010000
H	1.67374500	4.43939600	-1.25709100
H	3.78200200	3.15309100	-1.07918700
H	4.97198200	1.15597400	-0.42946200
H	4.96909000	-1.16823300	0.42947000
H	3.77413500	-3.16236000	1.07928100
H	1.66266700	-4.44336400	1.25734000
H	-0.68036100	-4.67738400	0.80050800
H	-2.75487800	-3.89116700	-0.27784700
H	-3.86188100	-2.35432900	-1.78904900
H	0.22340400	0.41443700	-2.10194000
H	-1.70315400	0.93687400	-3.49092800

H	-3.80250300	-0.39516500	-3.30050500
H	-3.85593100	2.36392300	1.78929700
H	0.22228400	-0.41534300	2.10141100
H	-1.70552700	-0.93311500	3.49042600
H	-3.80146300	0.40434000	3.30039500

5. Frontier MOs of compounds 1-3

	**1-CF_3**	**1-CN**	**1-H**
**ESPs**<br>0.03<br>-0.03	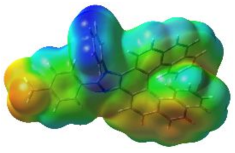	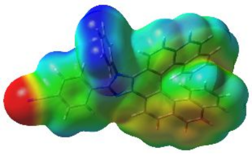	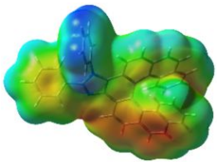
	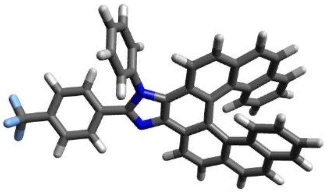	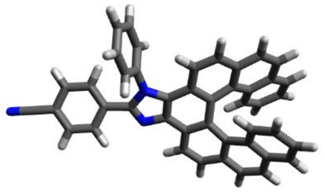	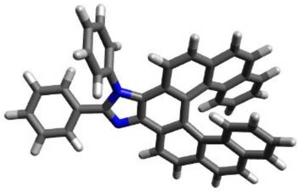
**LUMO+1**<br>**(eV)**	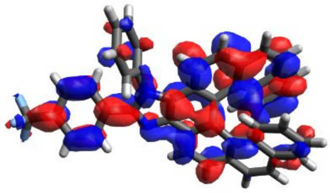<br>-1.87	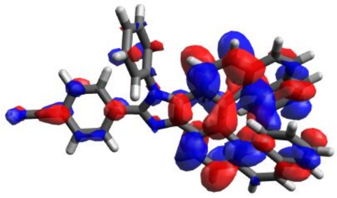<br>-1.97	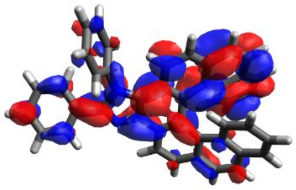<br>-1.62
**LUMO**<br>**(eV)**	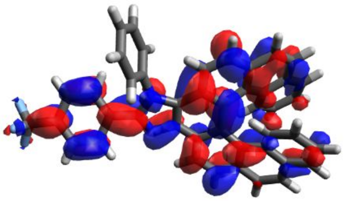<br>-2.05	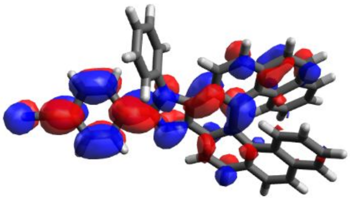<br>-2.27	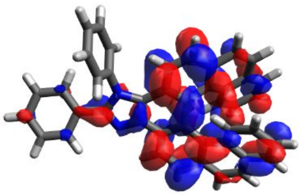<br>-1.85
**HOMO**<br>**(eV)**	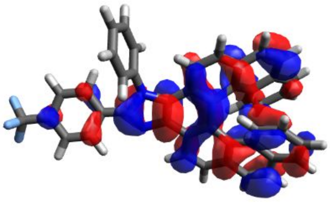<br>-5.54 (E_g = 3.49)	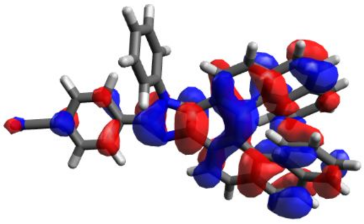<br>-5.60 (E_g = 3.33)	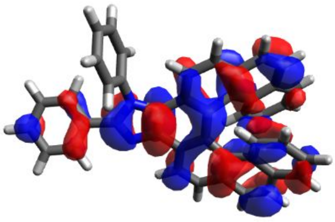<br>-5.37 (E_g = 3.52)
**HOMO–1**<br>**(eV)**	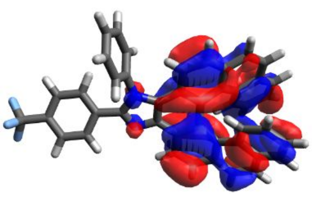<br>-5.74	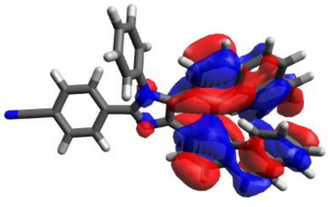<br>-5.79	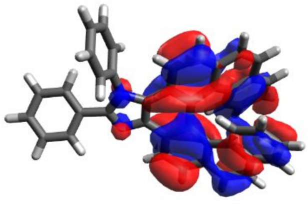<br>-5.60

	**2-CF$_3$**	**2-CN**	**2-H**
**ESPs** 0.03 -0.03	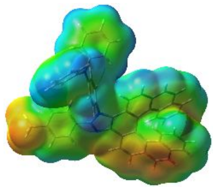	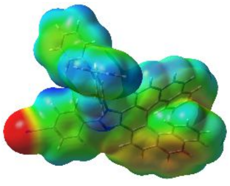	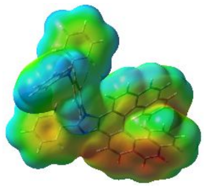
	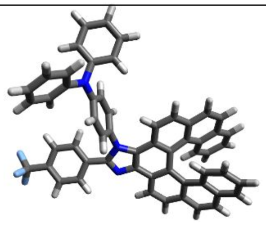	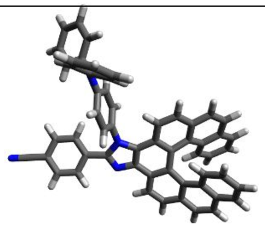	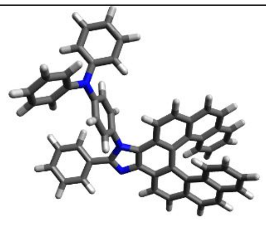
**LUMO+1 (eV)**	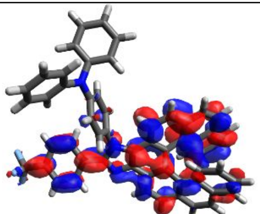 -1.81	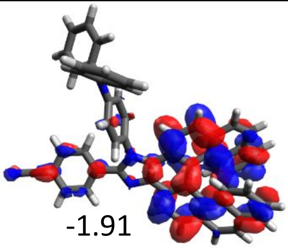 -1.91	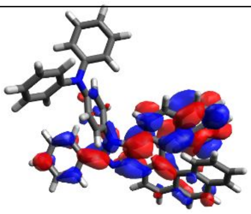 -1.57
**LUMO (eV)**	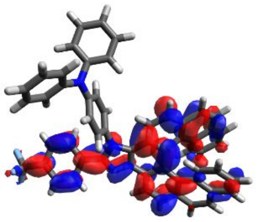 -2.00	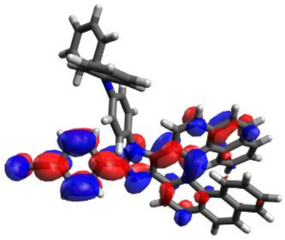 -2.21	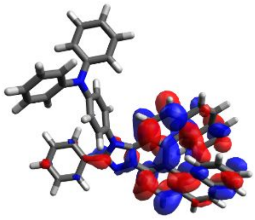 -1.81
**HOMO (eV)**	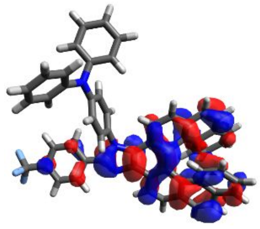 -5.49 (E_g = 3.49)	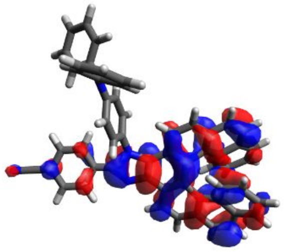 -5.53 (E_g = 3.32)	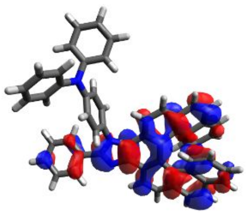 -5.33 (E_g = 3.52)
**HOMO–1 (eV)**	- 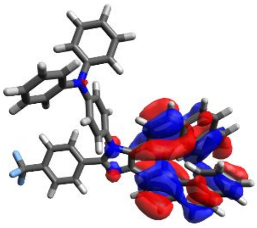 5.69	- 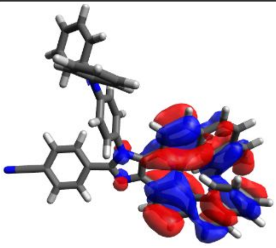 5.73	-5.56 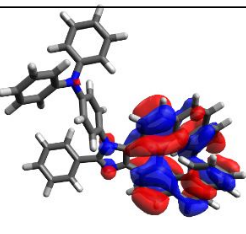

	**3-CF_3**	**3-CN**	**3-H**
**ESPs**<br>0.03<br>-0.03	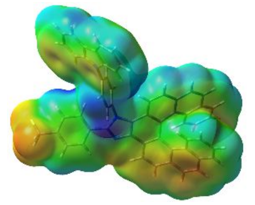	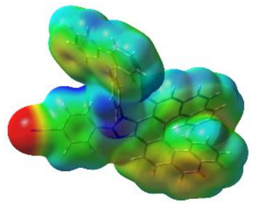	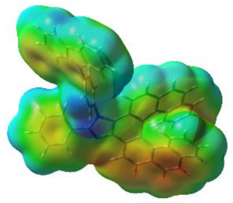
	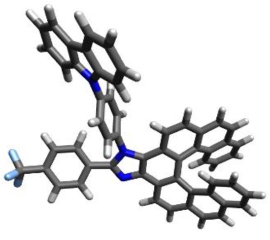	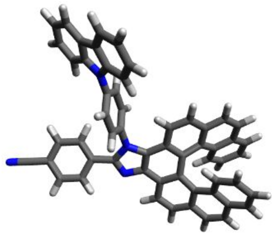	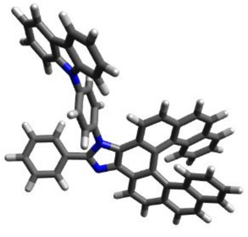
**LUMO+1**<br>**(eV)**	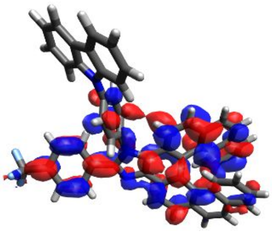<br>-1.93	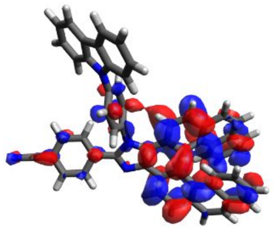<br>-2.02	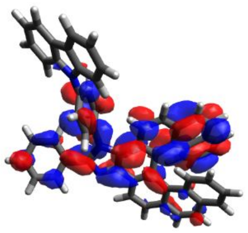<br>-1.69
**LUMO**<br>**(eV)**	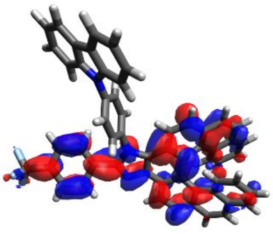<br>-2.11	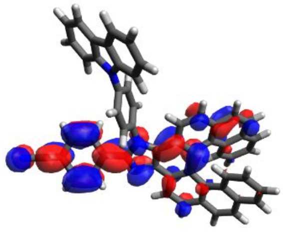<br>-2.32	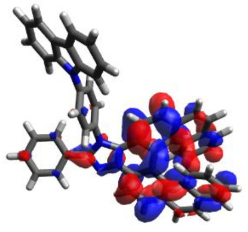<br>-1.90
**HOMO**<br>**(eV)**	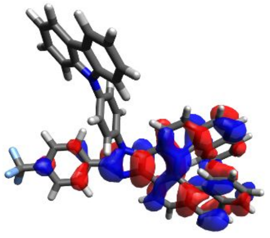<br>-5.59 (E_g = 3.48)	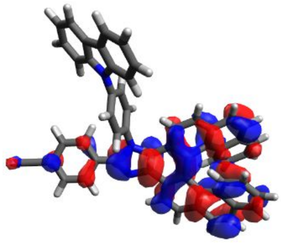<br>-5.64 (E_g = 3.32)	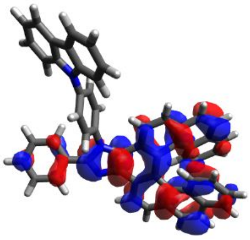<br>-5.43 (E_g = 3.53)
**HOMO–1**<br>**(eV)**	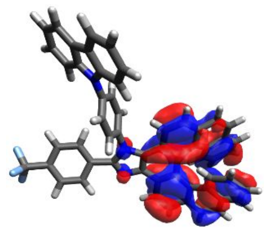<br>-5.78	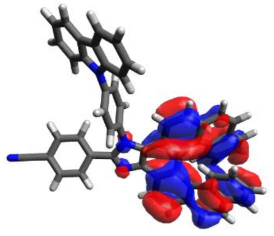<br>-5.83	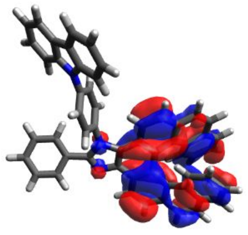<br>-5.66

6. Absorption and Luminiscence spectra

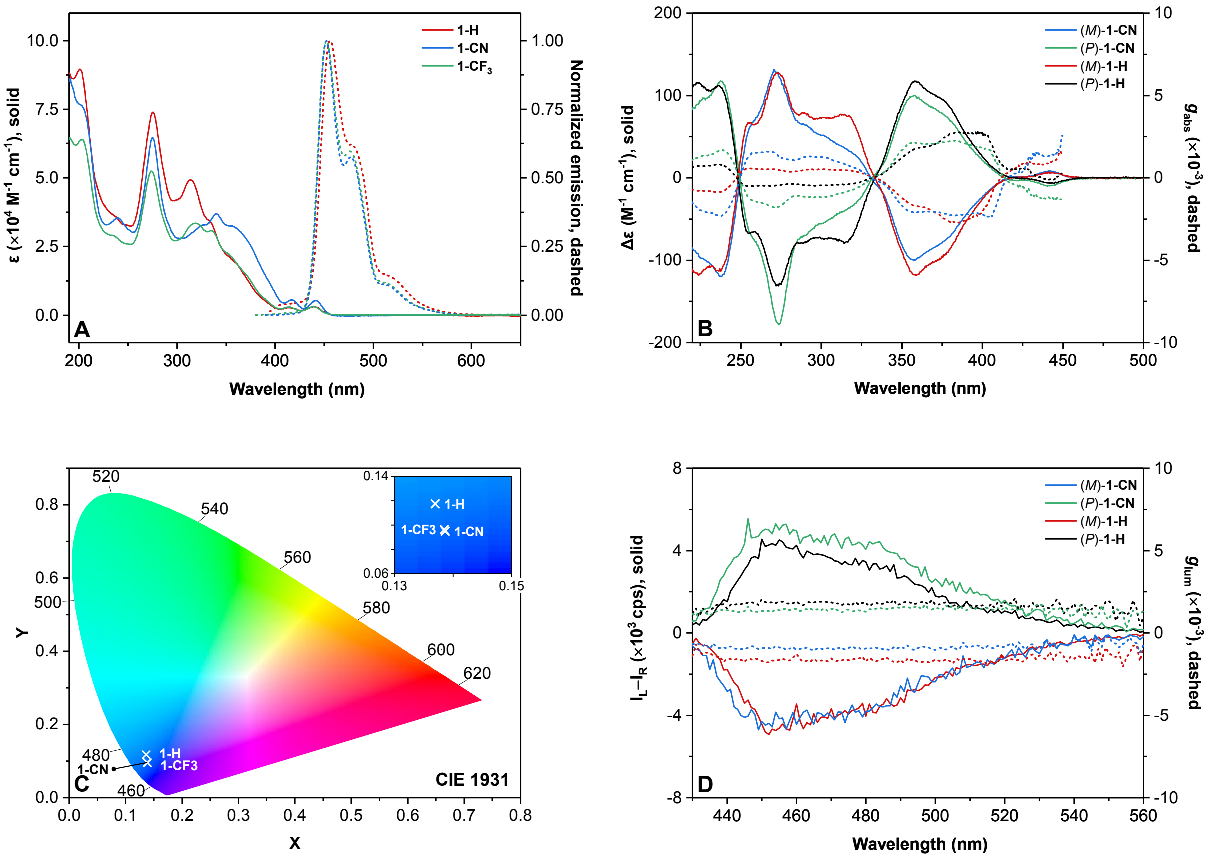

Figure S22 Optical properties of **1-H** and **1-CN**. a) UV/Vis and normalized emission spectra, b) CD spectra and g_{abs}, c) x,y coordinates of fluorescence emission with zoomed blue-region inset in CIE1931 system, d) ΔI spectra and g_{lum}.

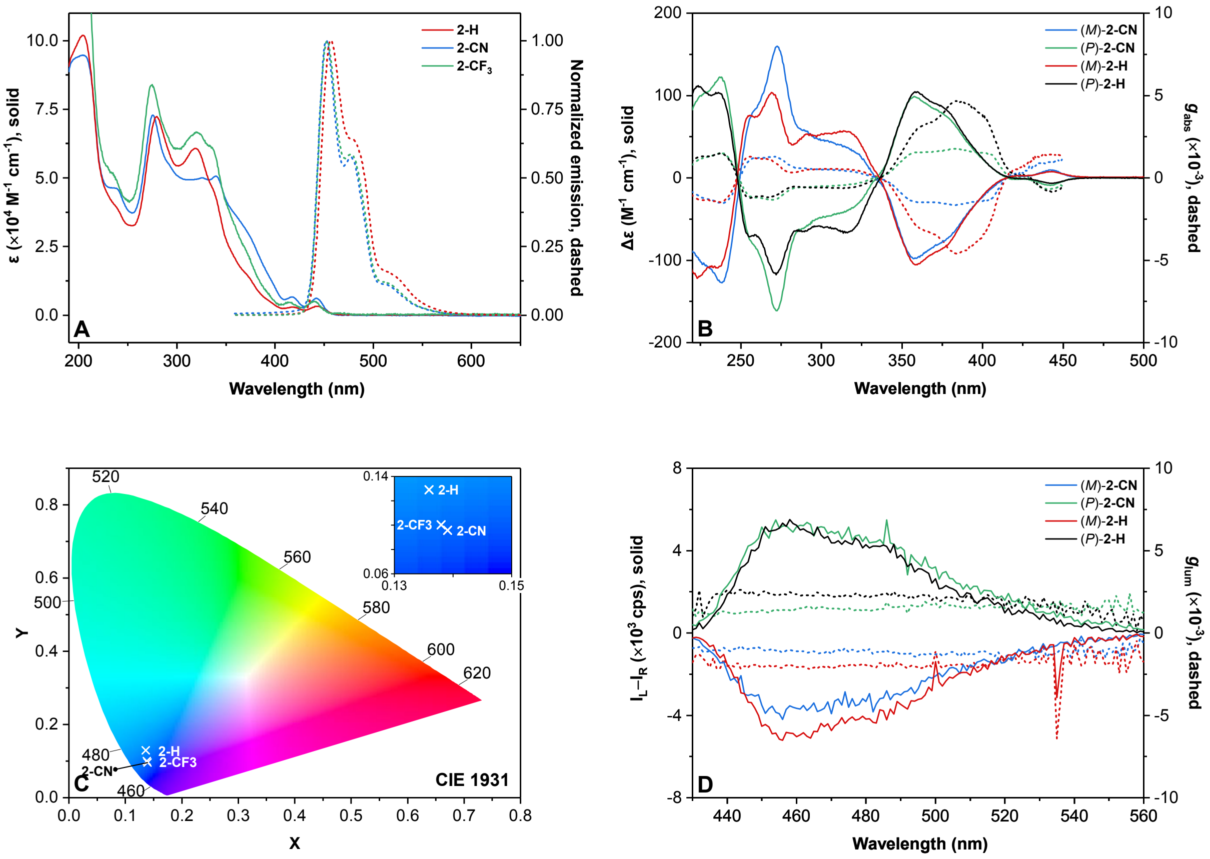

Figure S23 Optical properties of **2-H** and **2-CN**. a) UV/Vis and normalized emission spectra, b) CD spectra and g_{abs}, c) x,y coordinates of fluorescence emission with zoomed blue-region inset in CIE1931 system, d) ΔI spectra and g_{lum}.

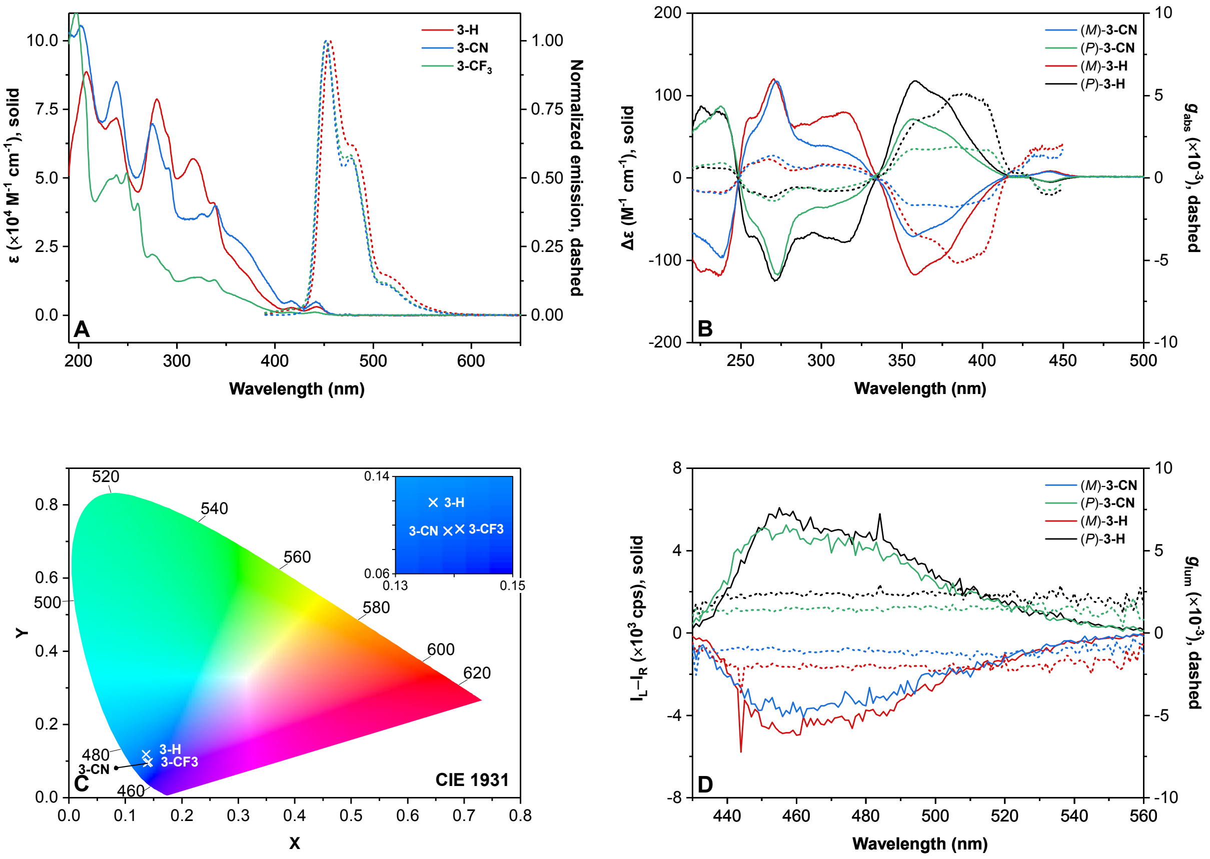

Figure S24 Optical properties of **3-H** and **3-CN**. a) UV/Vis and normalized emission spectra, b) CD spectra and g_{abs}, c) x,y coordinates of fluorescence emission with zoomed blue-region inset in CIE1931 system, d) ΔI spectra and g_{lum}.

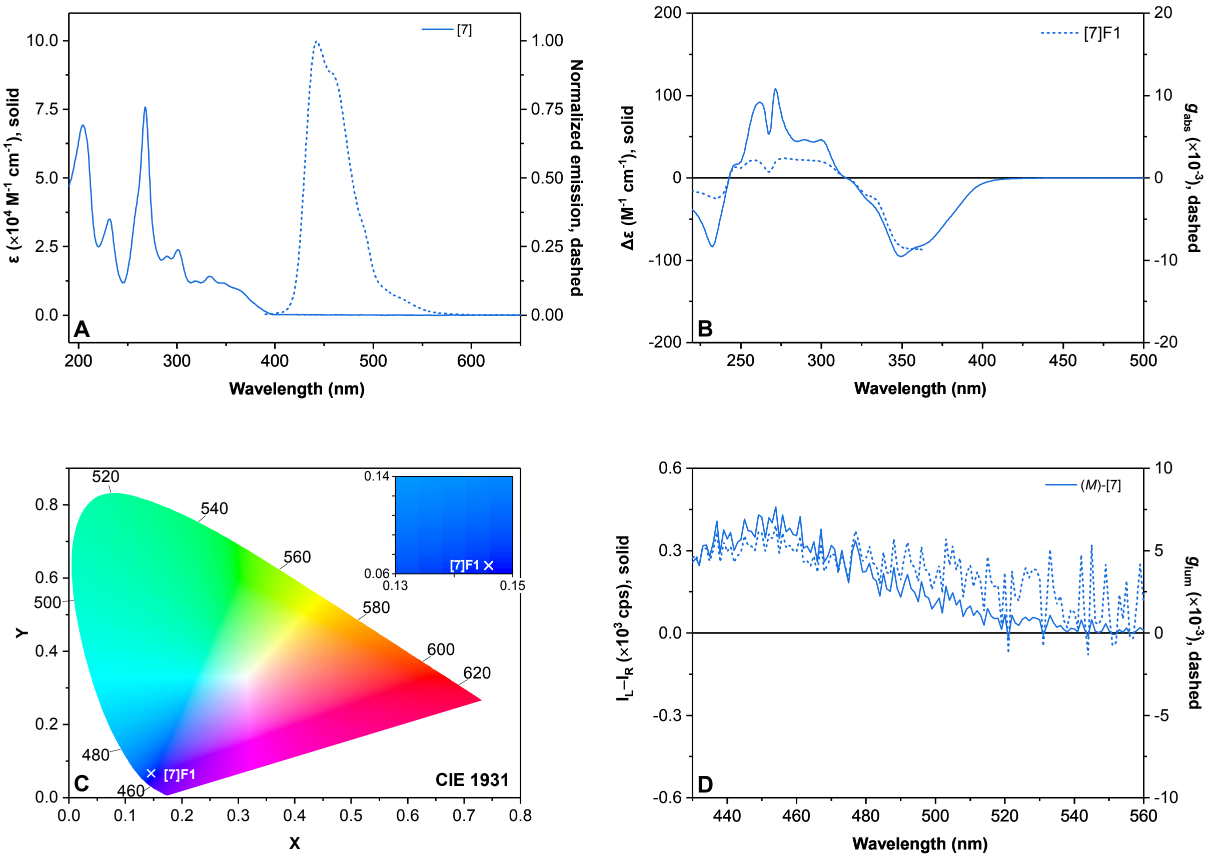

Figure S25 Optical properties of (*M*)-[7]helicene. a) UV/Vis and normalized emission spectra, b) CD spectra and g_{abs}, c) x,y coordinates of fluorescence emission with zoomed blue-region inset in CIE1931 system, d) ΔI spectra and g_{lum}.

7. Summary of calculated and experimental properties

Table S1: Calculated and experimental properties of **1-H** – **3-H**, **1-CN** – **3-CN**, **1-CF$_3$** – **3-CF$_3$** and **[7]**.

	LUMO (eV)	HOMO (eV)	ΔE	ε_{340}^{a} (M^{-1}cm^{-1})	λ_{em}^{b} (nm)	Φ_{340}^{c} %	$\lvert g_{abs,calc}\rvert$ (×10^{-3})	$\lvert g_{lum,calc}\rvert$ (×10^{-3})	$\lvert\mu\rvert$ (×10^{-20} esu cm)	$\lvert m\rvert$ (×10^{-20} erg G^{-1})	$\lvert\theta_{\mu m}\rvert$ (°)	$\lvert g_{abs}^{max}\rvert^{d}$ (×10^{-3})	$\lvert g_{lum}^{max}\rvert^{d}$ (×10^{-3})	B_{CPL}
**1-H**	-1.848	-5.372	3.524	29 019	456	16.1	16.4	3.76	230.3	0.255	154.91	2.76	1.79	4.2
**1-CN**	-2.272	-5.598	3.326	36 959	453	19.9	5.9	1.27	529.0	0.259	133.95	2.29	1.26	5.1
**1-CF$_3$**	-2.054	-5.545	3.491	28 648	452	14.9	6.7	2.82	283.5	0.228	159.54	-	-	-
**2-H**	-1.805	-5.327	3.522	42 569	457	18.3	18.1	3.63	240.4	0.453	120.77	4.64	2.3	9
**2-CN**	-2.212	-5.535	3.323	50 623	452	22.1	6.0	1.25	562.8	0.655	106.58	1.7	1.47	8.3
**2-CF$_3$**	-2	-5.487	3.487	57248	453	18.8	7.0	2.78	289.2	0.421	120.63	-	-	-
**3-H**	-1.905	-5.434	3.529	38 865	456	15.3	9.6	3.99	227.8	0.444	123	5.09	2.32	6.9
**3-CN**	-2.327	-5.644	3.317	39 586	452	21.6	5.7	1.13	607.3	0.682	105.34	1.82	1.46	6.3
**3-CF$_3$**	-2.108	-5.594	3.486	12 530	452	17.6	6.9	3.16	276.9	0.404	125.3	-	-	-
**[7]**[31]	-1.894	-5.701	3.806	12 256[e]	443[e]	1.7[e]	46.5	16.27	19.3	0.085	90	8.57[e]	5.53[e]	0.6

[a]Extinction at 340 nm, 10^{-5}-10^{-4} M concentration range in acetonitrile, 2mm optical pathway. [b]Excitation wavelengths 340-370 nm, 10^{-5} M concentration range in acetonitrile. [c]Corrected absolute quantum yields upon excitation at 340 nm. [d]Absolute value as the average of the experimentally obtained data from both enantiomers. [e]Experimentally obtained value from (*M*)-[7]helicene enantiomer.

8. Chiral-HPLC Analysis

Chromatogram

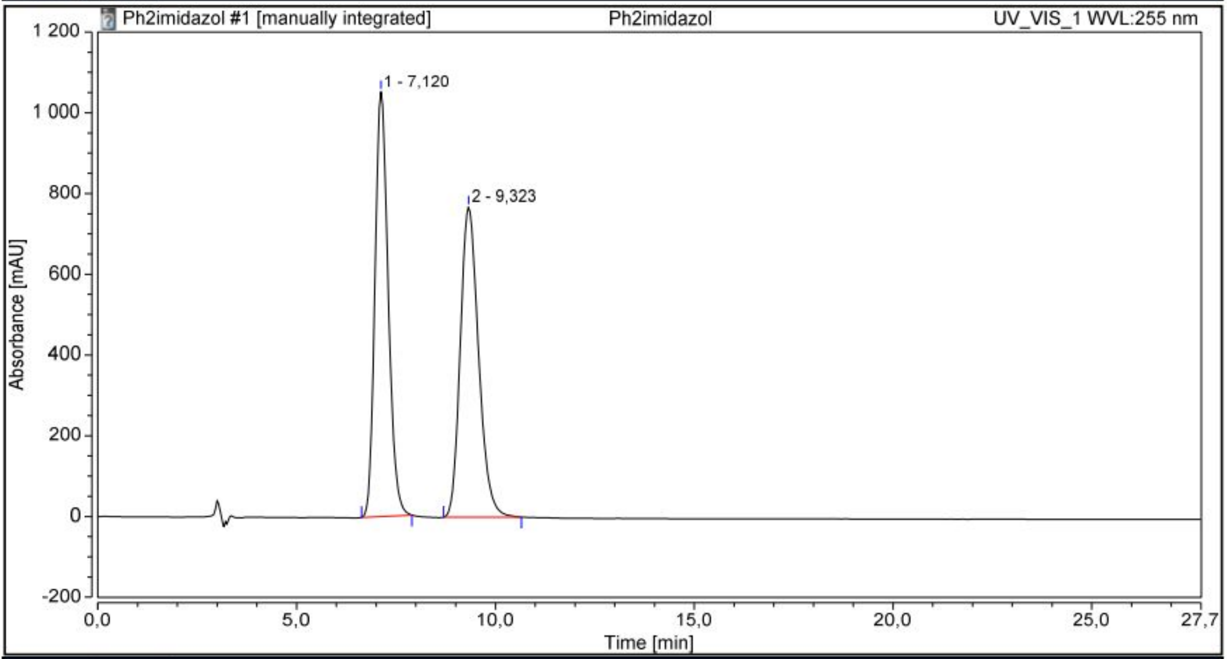

Integration Results

No.	Peak Name	Retention Time min	Area mAU*min	Height mAU	Relative Area %	Relative Height %	Amount n.a.
1		7,120	405,257	1052,393	49,76	57,80	n.a.
2		9,323	409,187	768,278	50,24	42,20	n.a.
**Total:**			**814,444**	**1820,671**	**100,00**	**100,00**	

Figure S26 HPLC chromatogram of (*rac*)-**1-H**. Chiralpak® IC, Heptane/DCM/MTBE (70:20:30).

Chromatogram

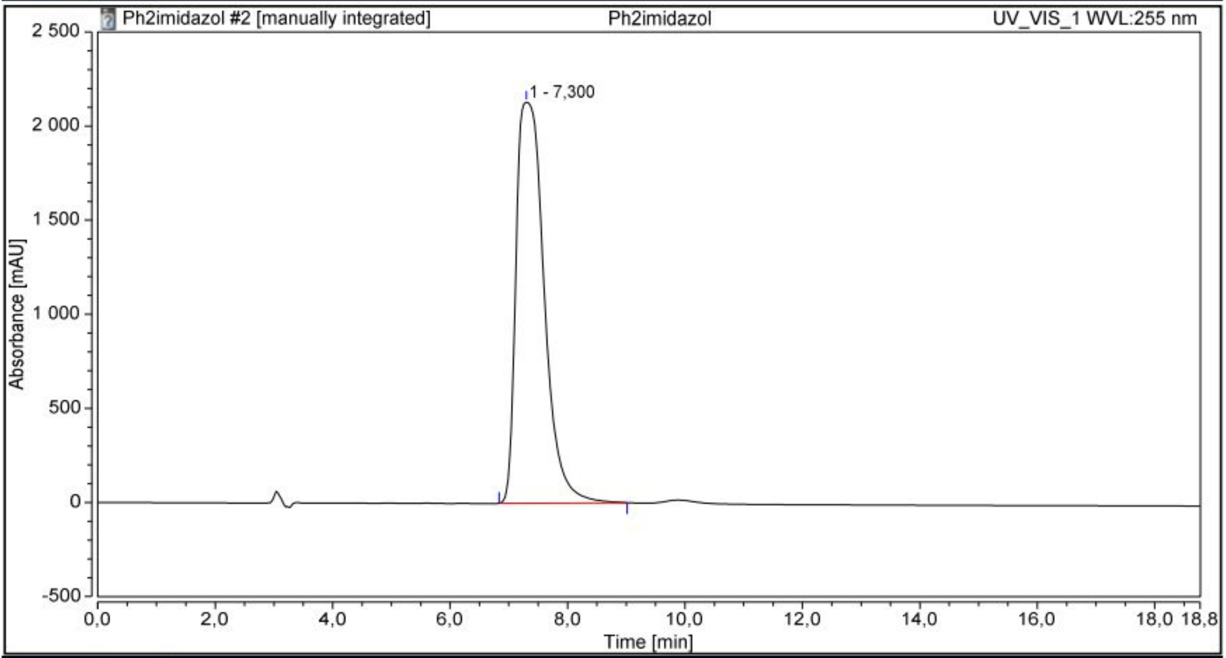

Integration Results

No.	Peak Name	Retention Time min	Area mAU*min	Height mAU	Relative Area %	Relative Height %	Amount n.a.
1		7,300	1180,536	2130,138	100,00	100,00	n.a.
**Total:**			**1180,536**	**2130,138**	**100,00**	**100,00**	

Figure S27 HPLC chromatogram of (*P*)-**1-H**. Chiralpak® IC, Heptane/DCM/MTBE (70:20:30).

Chromatogram

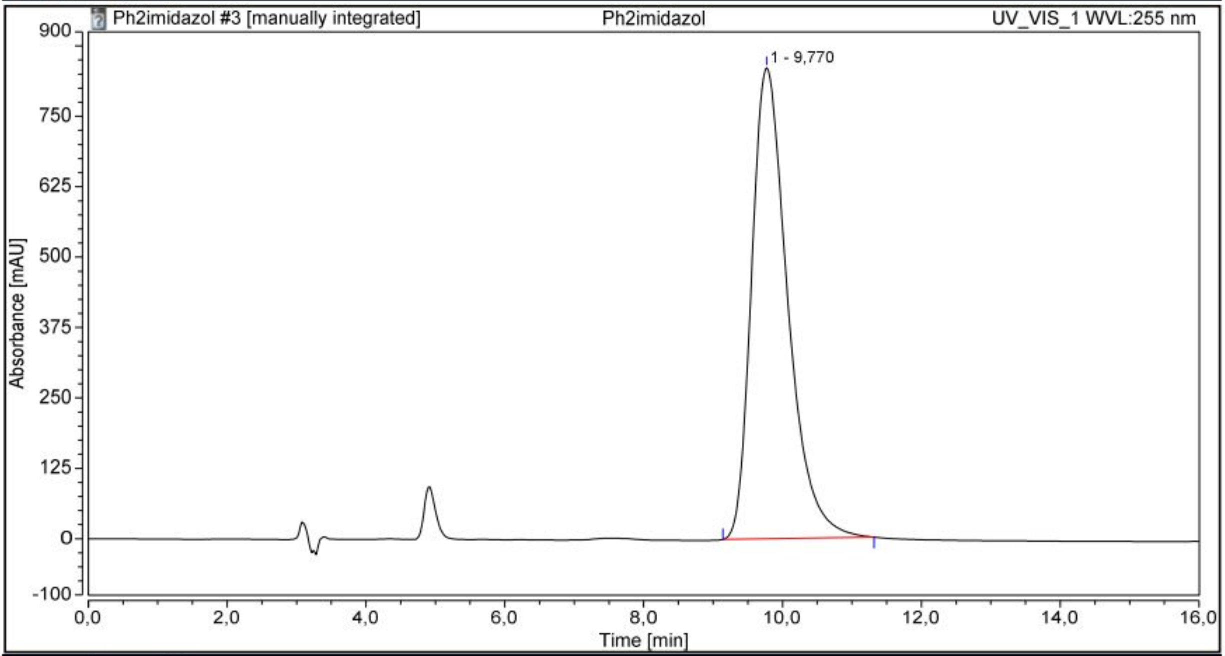

Integration Results

No.	Peak Name	Retention Time min	Area mAU*min	Height mAU	Relative Area %	Relative Height %	Amount n.a.
1		9,770	503,769	836,698	100,00	100,00	n.a.
**Total:**			**503,769**	**836,698**	**100,00**	**100,00**	

Figure S28 HPLC chromatogram of (*M*)-**1-H**. Chiralpak® IC, Heptane/DCM/MTBE (70:20:30).

Chromatogram

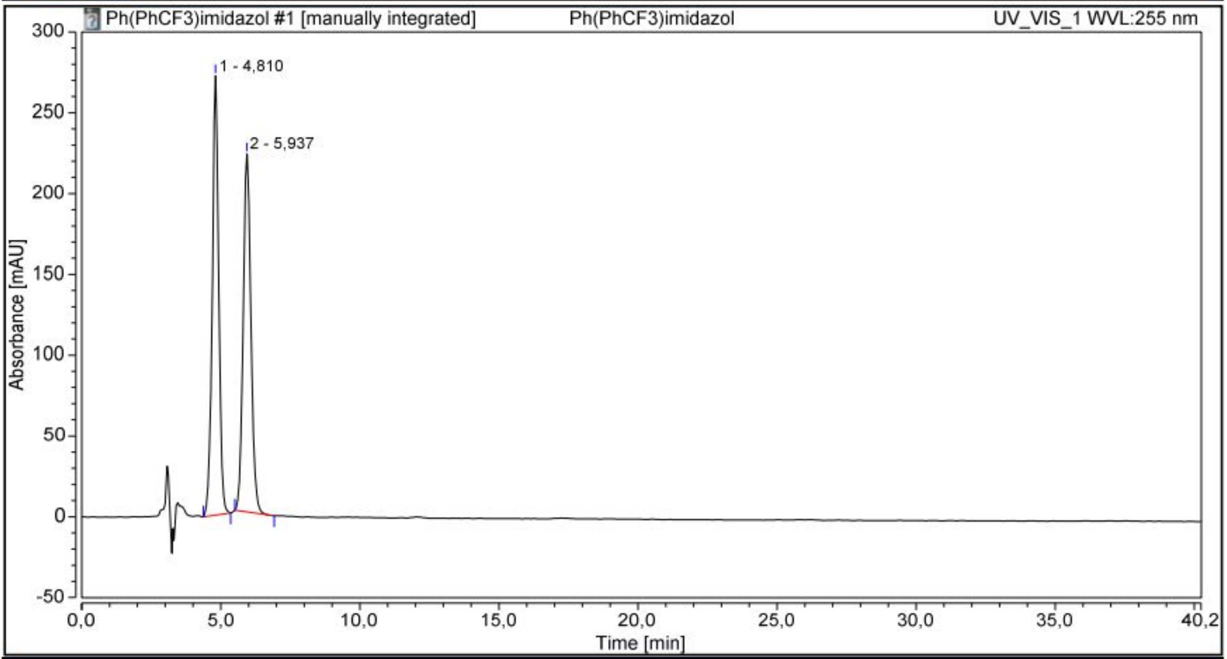

Integration Results

No.	Peak Name	Retention Time min	Area mAU*min	Height mAU	Relative Area %	Relative Height %	Amount n.a.
1		4,810	72,637	271,705	50,57	55,09	n.a.
2		5,937	70,998	221,490	49,43	44,91	n.a.
**Total:**			**143,635**	**493,195**	**100,00**	**100,00**	

Figure S29 HPLC chromatogram of (*rac*)-**1-CF$_3$**. Chiralpak® IC, Heptane/DCM/MTBE (70:20:30).

Chromatogram

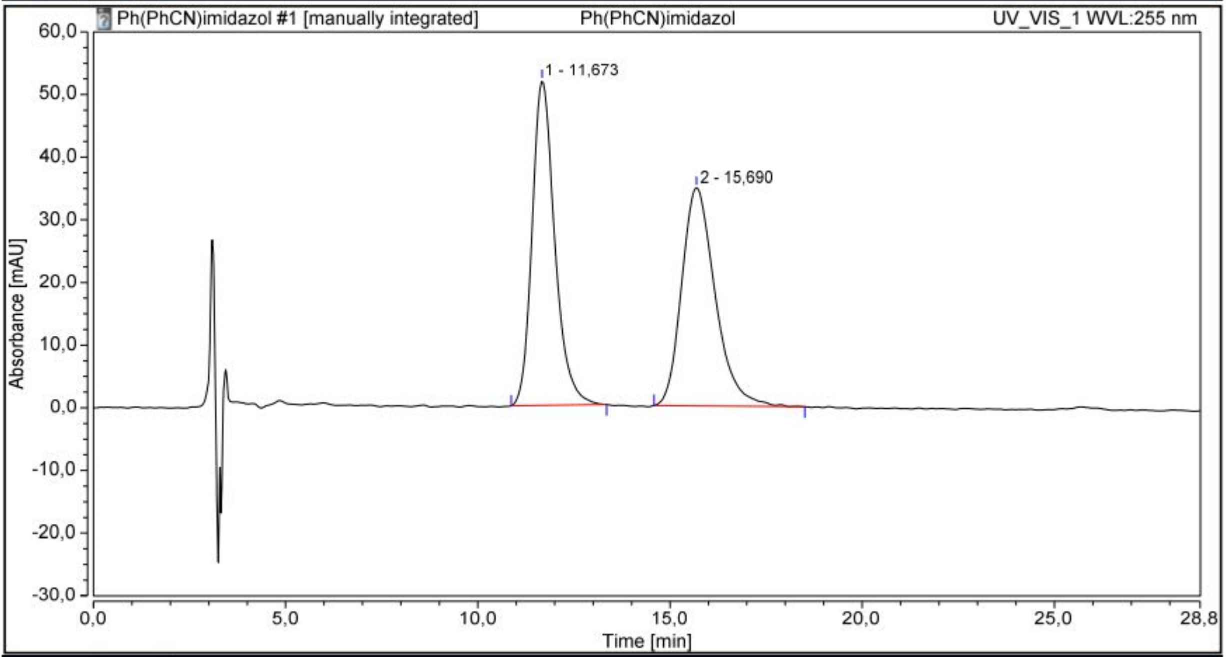

Integration Results

No.	Peak Name	Retention Time min	Area mAU*min	Height mAU	Relative Area %	Relative Height %	Amount n.a.
1		11,673	35,395	51,831	50,18	59,81	n.a.
2		15,690	35,136	34,833	49,82	40,19	n.a.
**Total:**			**70,531**	**86,663**	**100,00**	**100,00**	

Figure S30 HPLC chromatogram of (*rac*)-**1-CN**. Chiralpak® IC, Heptane/DCM/MTBE (70:20:30).

Chromatogram

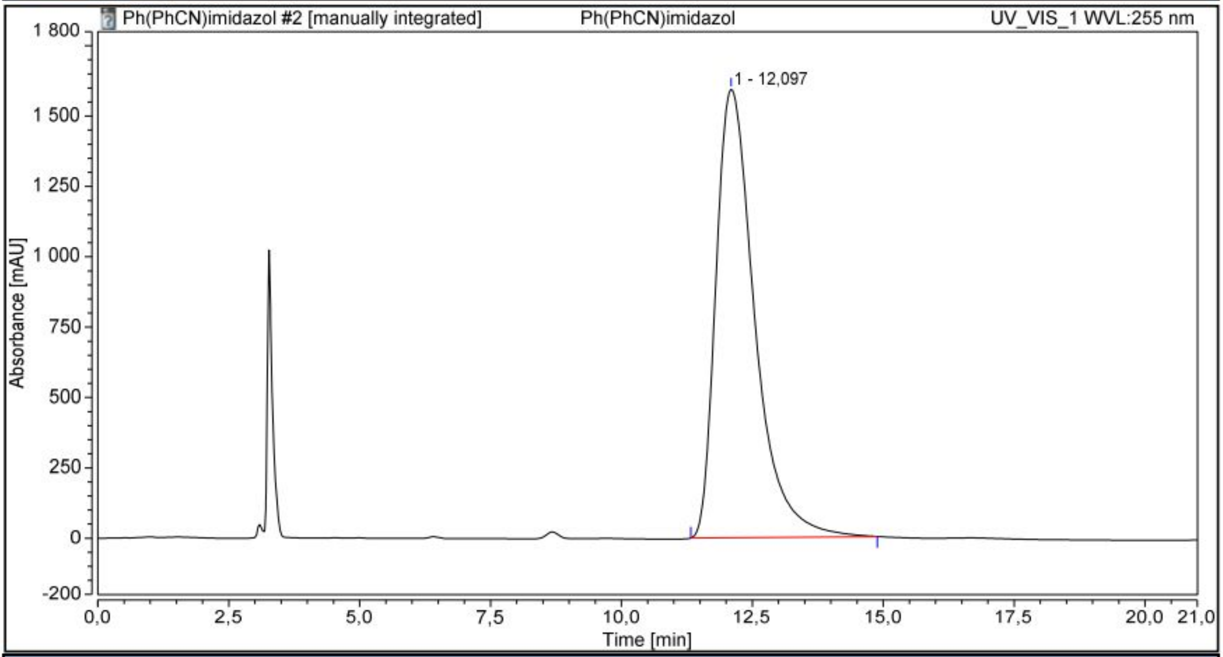

Integration Results

No.	Peak Name	Retention Time min	Area mAU*min	Height mAU	Relative Area %	Relative Height %	Amount n.a.
1		12,097	1367,281	1593,959	100,00	100,00	n.a.
**Total:**			**1367,281**	**1593,959**	**100,00**	**100,00**	

Figure S31 HPLC chromatogram of (*P*)-**1-CN**. Chiralpak® IC, Heptane/DCM/MTBE (70:20:30).

Chromatogram

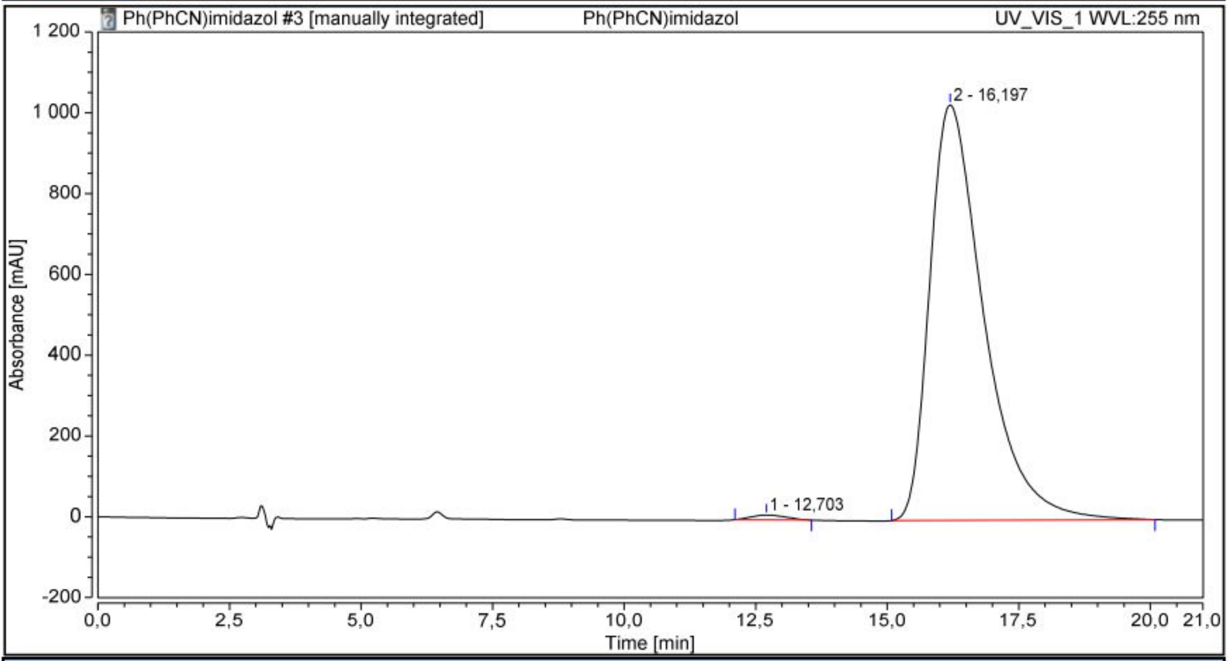

Integration Results

No.	Peak Name	Retention Time min	Area mAU*min	Height mAU	Relative Area %	Relative Height %	Amount n.a.
1		12,703	9,119	11,901	0,74	1,14	n.a.
2		16,197	1224,981	1028,264	99,26	98,86	n.a.
**Total:**			**1234,099**	**1040,165**	**100,00**	**100,00**	

Figure S32 HPLC chromatogram of (*M*)-**1-CN**. Chiralpak® IC, Heptane/DCM/MTBE (70:20:30).

Chromatogram

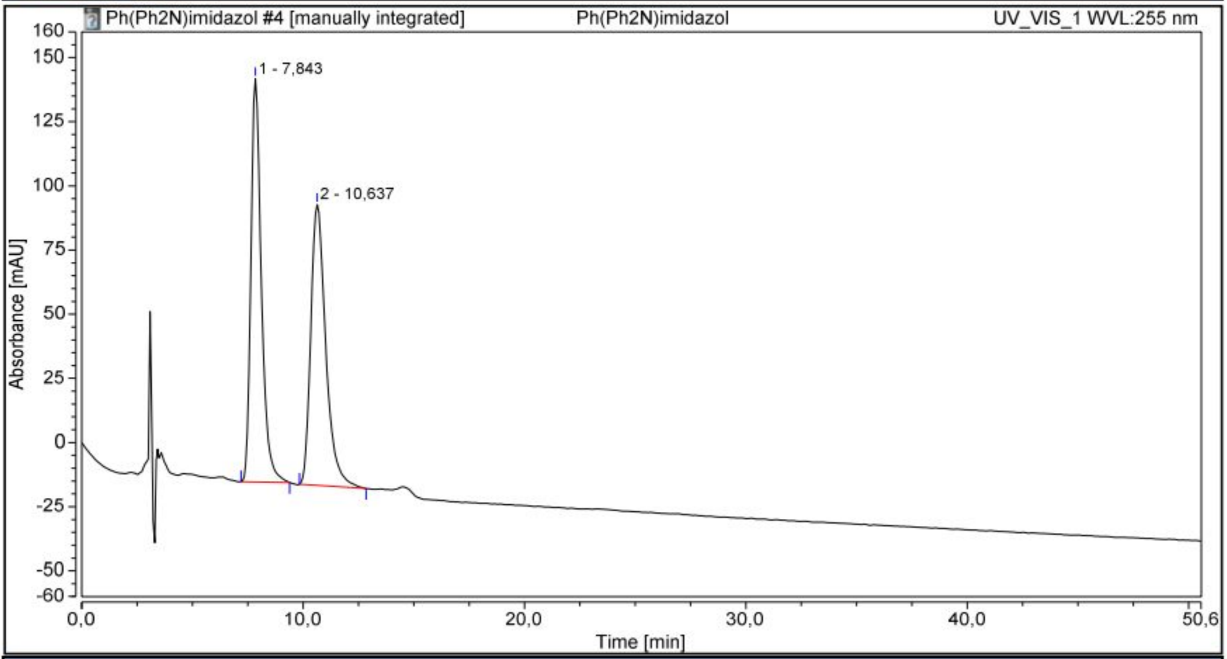

Integration Results

No.	Peak Name	Retention Time min	Area mAU*min	Height mAU	Relative Area %	Relative Height %	Amount n.a.
1		7,843	86,059	157,073	50,09	58,93	n.a.
2		10,637	85,739	109,485	49,91	41,07	n.a.
**Total:**			**171,798**	**266,558**	**100,00**	**100,00**	

Figure S33 HPLC chromatogram of (*rac*)-**2-H**. Chiralpak® IC, Heptane/DCM/MTBE (70:20:30).

Chromatogram

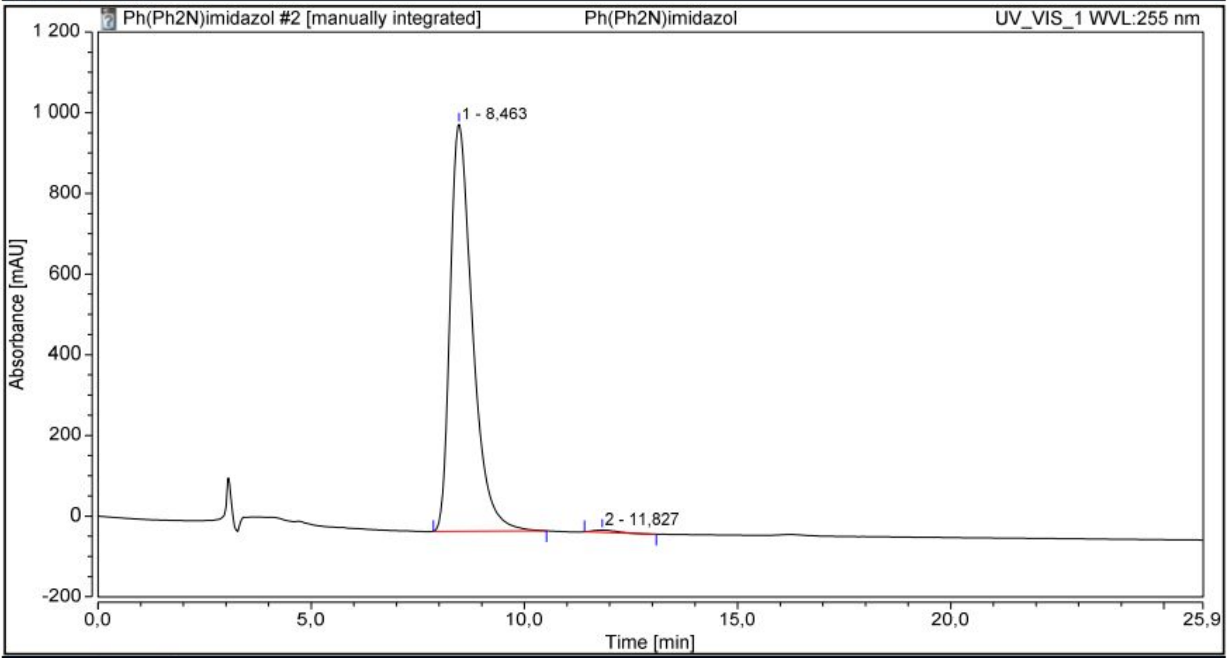

Integration Results

No.	Peak Name	Retention Time min	Area mAU*min	Height mAU	Relative Area %	Relative Height %	Amount n.a.
1		8,463	613,992	1008,937	99,43	99,45	n.a.
2		11,827	3,545	5,599	0,57	0,55	n.a.
**Total:**			**617,536**	**1014,537**	**100,00**	**100,00**	

Figure S34 HPLC chromatogram of (*P*)-**2-H**. Chiralpak® IC, Heptane/DCM/MTBE (70:20:30).

Chromatogram

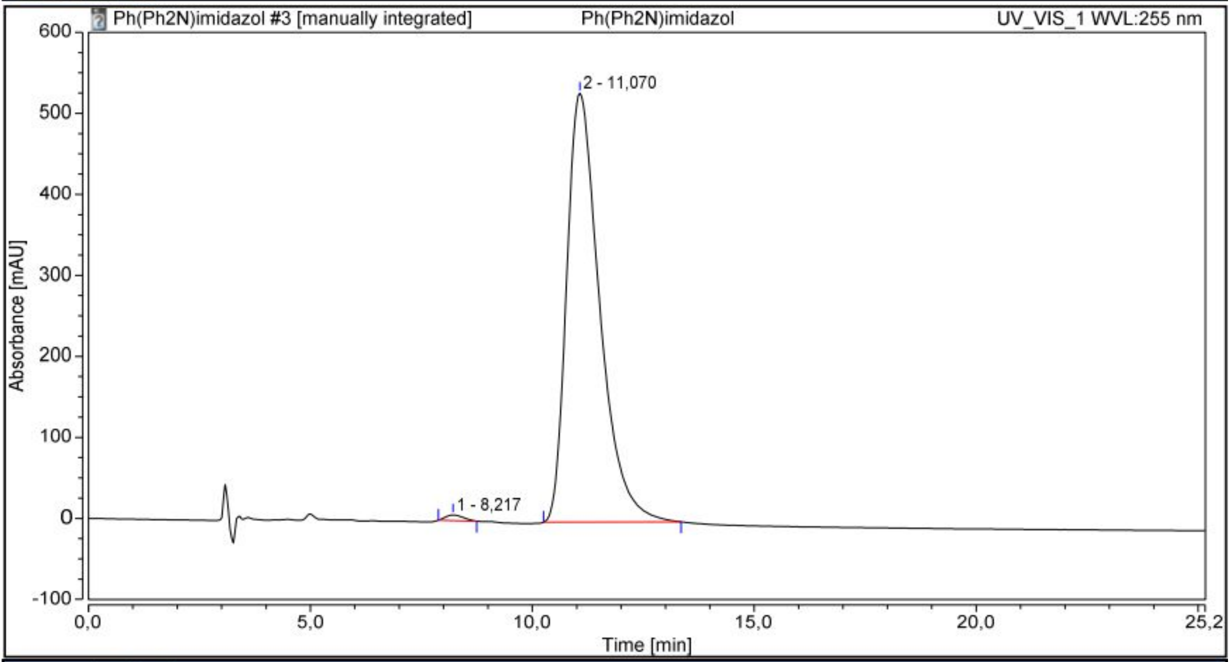

Integration Results

No.	Peak Name	Retention Time min	Area mAU*min	Height mAU	Relative Area %	Relative Height %	Amount n.a.
1		8,217	3,246	6,921	0,71	1,29	n.a.
2		11,070	456,248	529,538	99,29	98,71	n.a.
**Total:**			**459,494**	**536,458**	**100,00**	**100,00**	

Figure S35 HPLC chromatogram of (*M*)-**2-H**. Chiralpak® IC, Heptane/DCM/MTBE (70:20:30).

Chromatogram

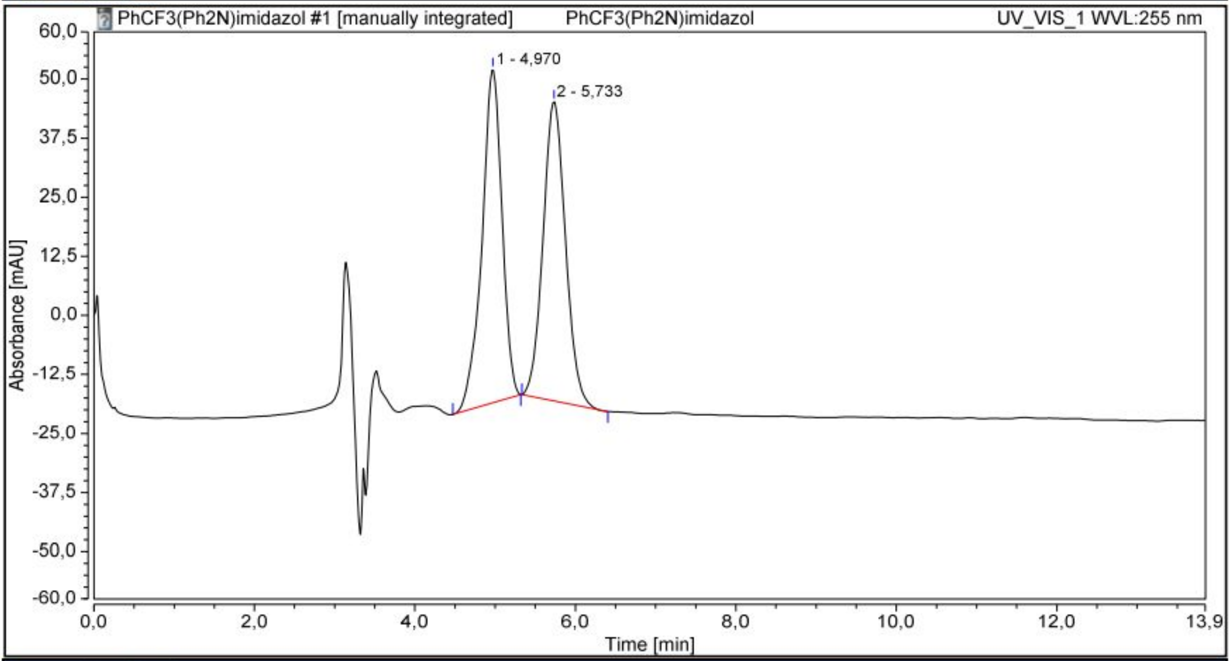

Integration Results

No.	Peak Name	Retention Time min	Area mAU*min	Height mAU	Relative Area %	Relative Height %	Amount n.a.
1		4,970	20,430	70,654	49,88	52,71	n.a.
2		5,733	20,528	63,387	50,12	47,29	n.a.
**Total:**			**40,957**	**134,041**	**100,00**	**100,00**	

Figure S36 HPLC chromatogram of (*rac*)-**2-CF$_3$**. Chiralpak® IC, Heptane/DCM/MTBE (70:20:30).

Chromatogram

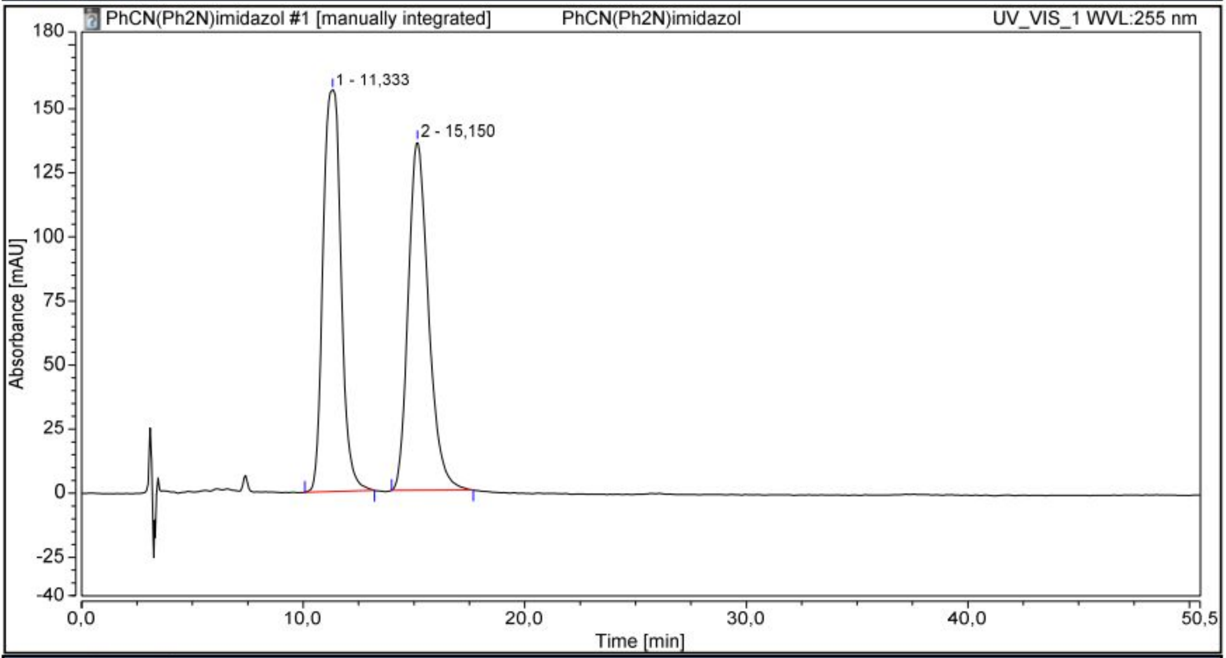

Integration Results

No.	Peak Name	Retention Time min	Area mAU*min	Height mAU	Relative Area %	Relative Height %	Amount n.a.
1		11,333	148,009	156,748	50,70	53,54	n.a.
2		15,150	143,910	136,003	49,30	46,46	n.a.
**Total:**			**291,919**	**292,752**	**100,00**	**100,00**	

Figure S37 HPLC chromatogram of (*rac*)-**2-CN**. Chiralpak® IC, Heptane/DCM/MTBE (70:20:30).

Chromatogram

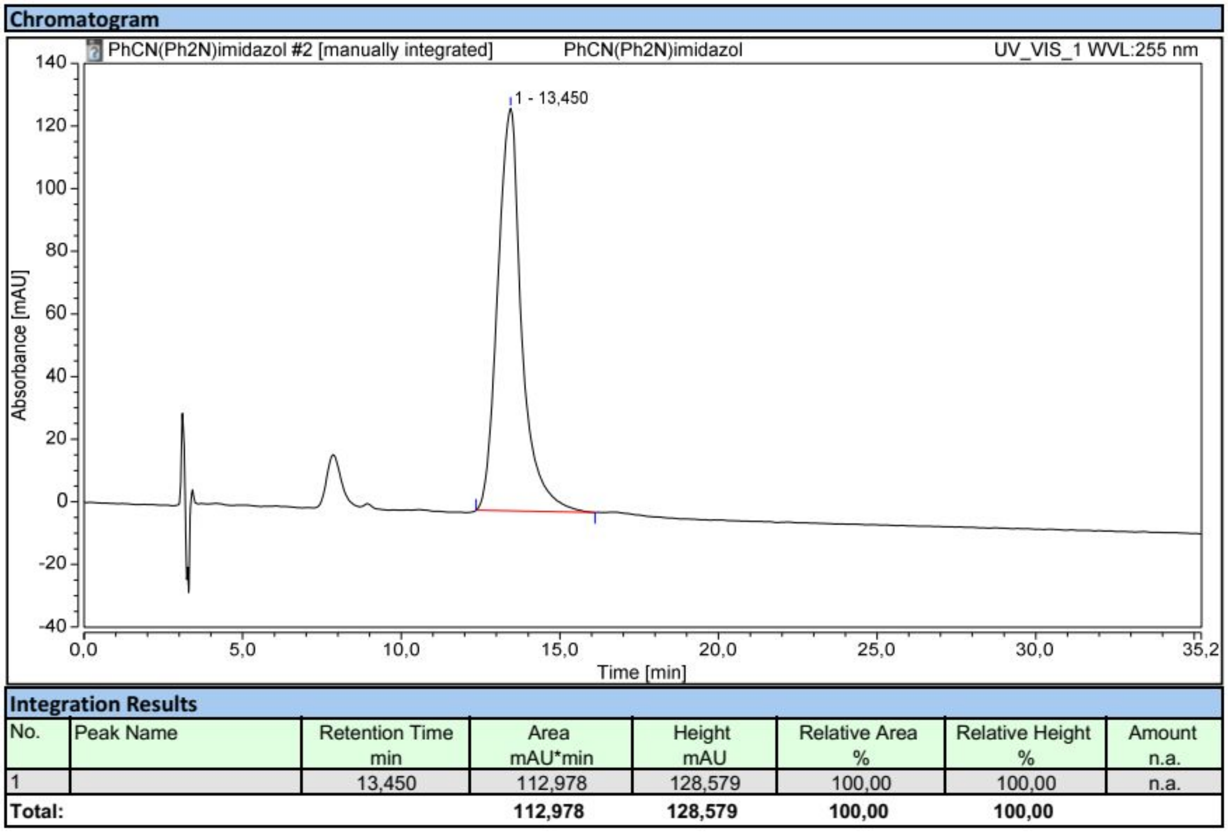

Integration Results

No.	Peak Name	Retention Time min	Area mAU*min	Height mAU	Relative Area %	Relative Height %	Amount n.a.
1		13,450	112,978	128,579	100,00	100,00	n.a.
**Total:**			**112,978**	**128,579**	**100,00**	**100,00**	

Figure S38 HPLC chromatogram of (*P*)-**2-CN**. Chiralpak® IC, Heptane/DCM/MTBE (70:20:30).

Chromatogram

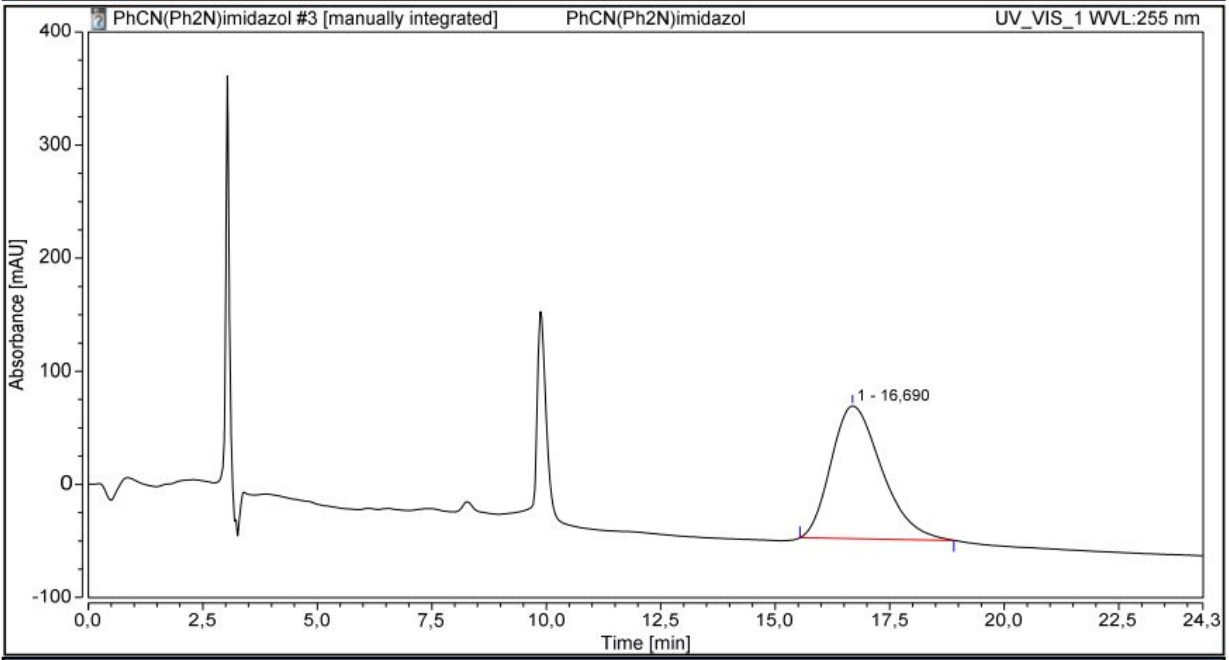

Integration Results

No.	Peak Name	Retention Time min	Area mAU*min	Height mAU	Relative Area %	Relative Height %	Amount n.a.
1		16,690	150,799	117,251	100,00	100,00	n.a.
**Total:**			**150,799**	**117,251**	**100,00**	**100,00**	

Figure S39 HPLC chromatogram of (*M*)-**2-CN**. Chiralpak® IC, Heptane/DCM/MTBE (70:20:30).

Chromatogram

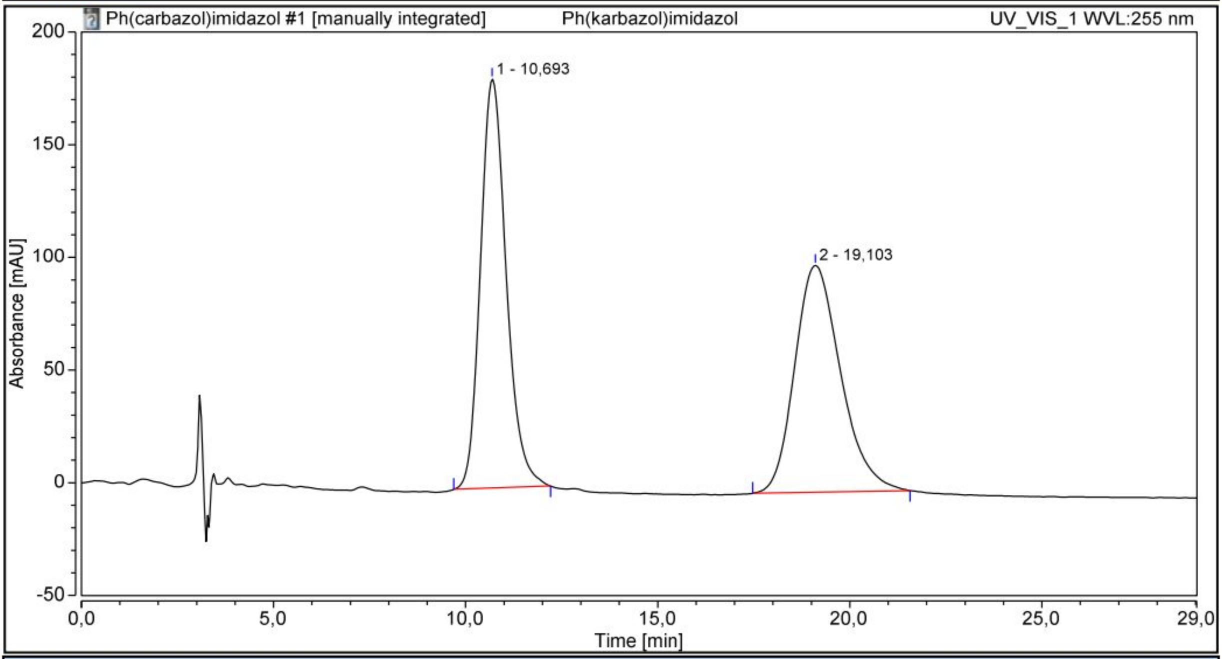

Integration Results

No.	Peak Name	Retention Time min	Area mAU*min	Height mAU	Relative Area %	Relative Height %	Amount n.a.
1		10,693	140,969	181,464	50,31	64,33	n.a.
2		19,103	139,226	100,637	49,69	35,67	n.a.
**Total:**			**280,195**	**282,101**	**100,00**	**100,00**	

Figure S40 HPLC chromatogram of (*rac*)-**3-H**. Chiralpak® IC, Heptane/DCM/MTBE (70:20:30).

Chromatogram

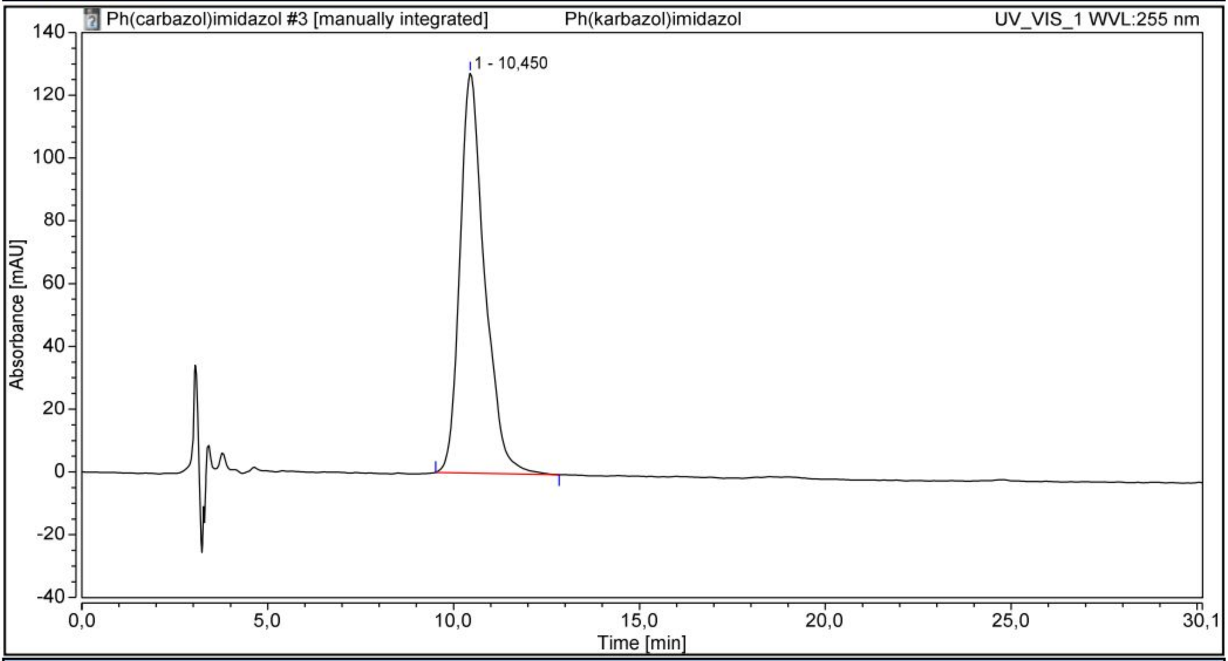

Integration Results

No.	Peak Name	Retention Time min	Area mAU*min	Height mAU	Relative Area %	Relative Height %	Amount n.a.
1		10,450	98,148	127,415	100,00	100,00	n.a.
**Total:**			**98,148**	**127,415**	**100,00**	**100,00**	

Figure S41 HPLC chromatogram of (*P*)-**3-H**. Chiralpak® IC, Heptane/DCM/MTBE (70:20:30).

Chromatogram

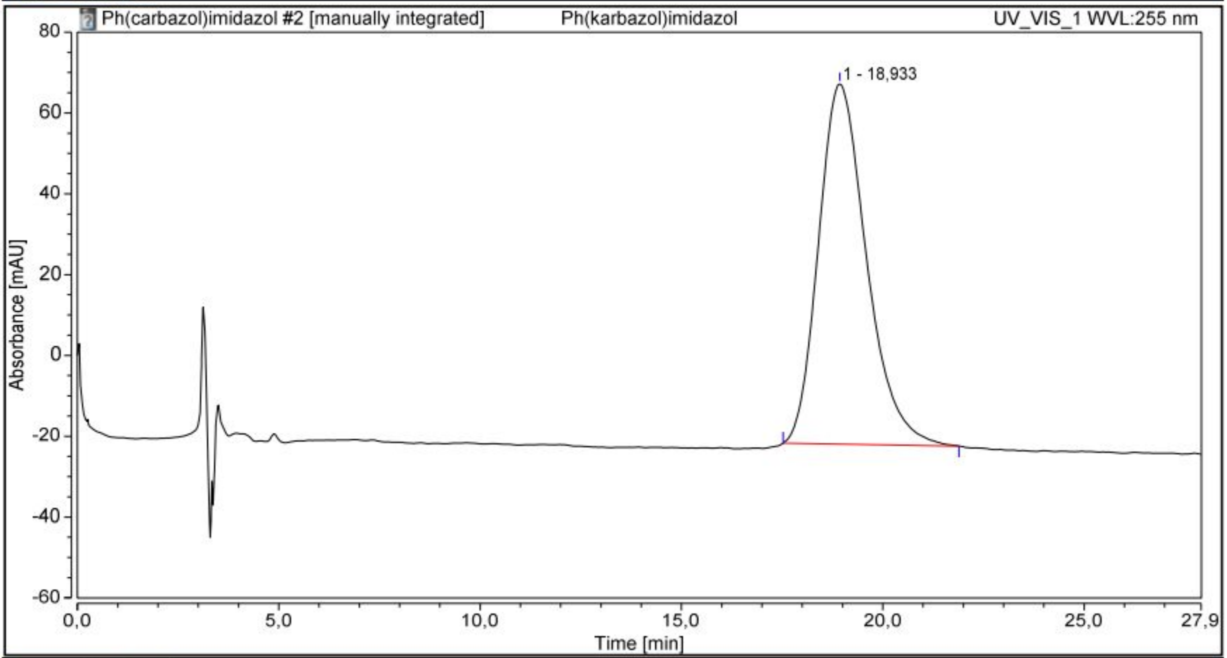

Integration Results

No.	Peak Name	Retention Time min	Area mAU*min	Height mAU	Relative Area %	Relative Height %	Amount n.a.
1		18,933	123,004	89,169	100,00	100,00	n.a.
**Total:**			**123,004**	**89,169**	**100,00**	**100,00**	

Figure S42 HPLC chromatogram of (*M*)-**3-H**. Chiralpak® IC, Heptane/DCM/MTBE (70:20:30).

Chromatogram

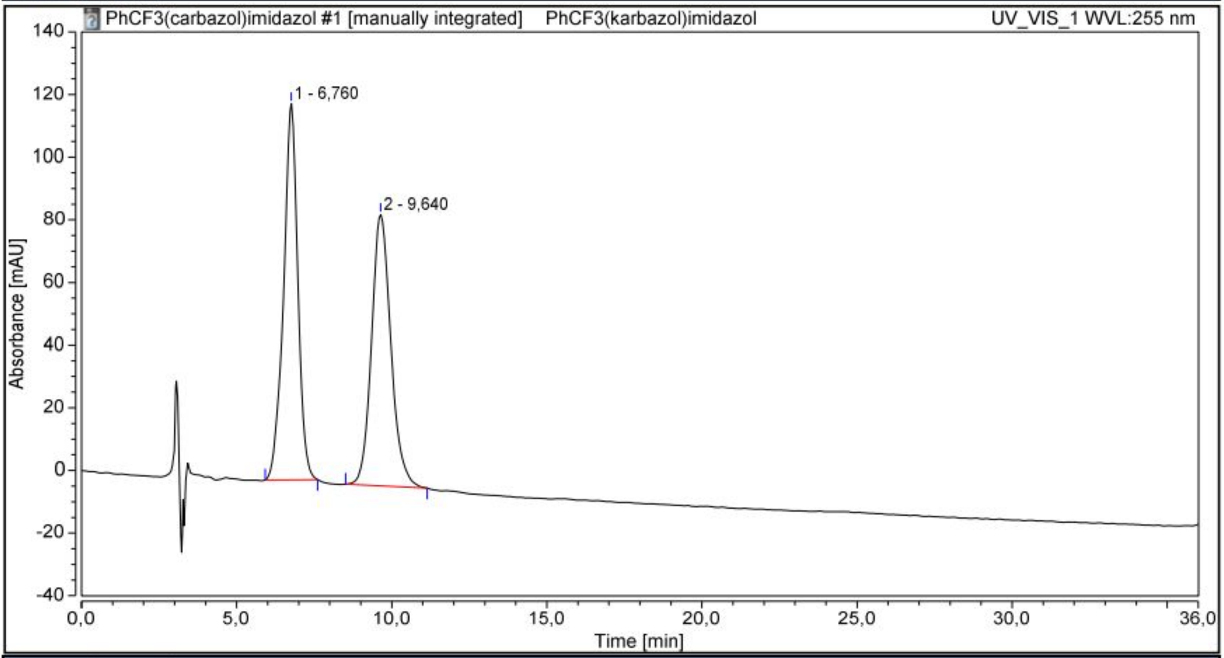

Integration Results

No.	Peak Name	Retention Time min	Area mAU*min	Height mAU	Relative Area %	Relative Height %	Amount n.a.
1		6,760	63,981	120,183	50,52	58,11	n.a.
2		9,640	62,656	86,649	49,48	41,89	n.a.
**Total:**			**126,638**	**206,832**	**100,00**	**100,00**	

Figure S43 HPLC chromatogram of (*rac*)-**3-CF$_3$**. Chiralpak® IC, Heptane/DCM/MTBE (70:20:30).

Chromatogram

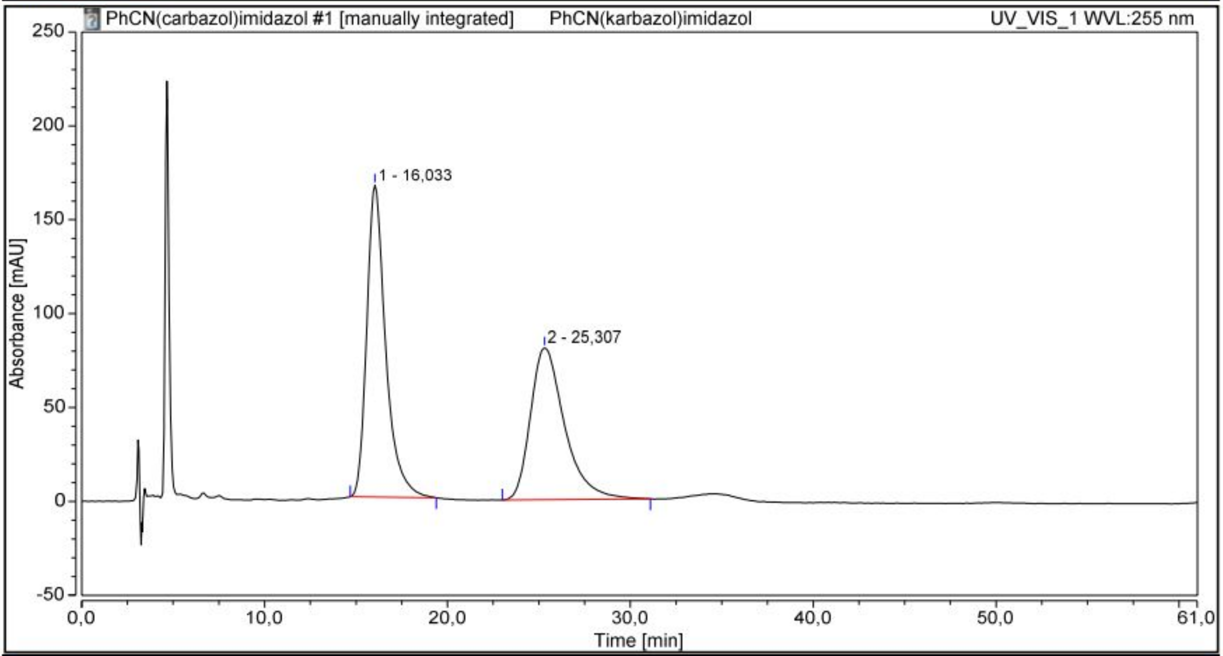

Integration Results

No.	Peak Name	Retention Time min	Area mAU*min	Height mAU	Relative Area %	Relative Height %	Amount n.a.
1		16,033	198,892	166,088	53,21	67,26	n.a.
2		25,307	174,863	80,839	46,79	32,74	n.a.
**Total:**			**373,755**	**246,927**	**100,00**	**100,00**	

Figure S44 HPLC chromatogram of (*rac*)-**3-CN**. Chiralpak® IC, Heptane/DCM/MTBE (70:20:30).

Chromatogram

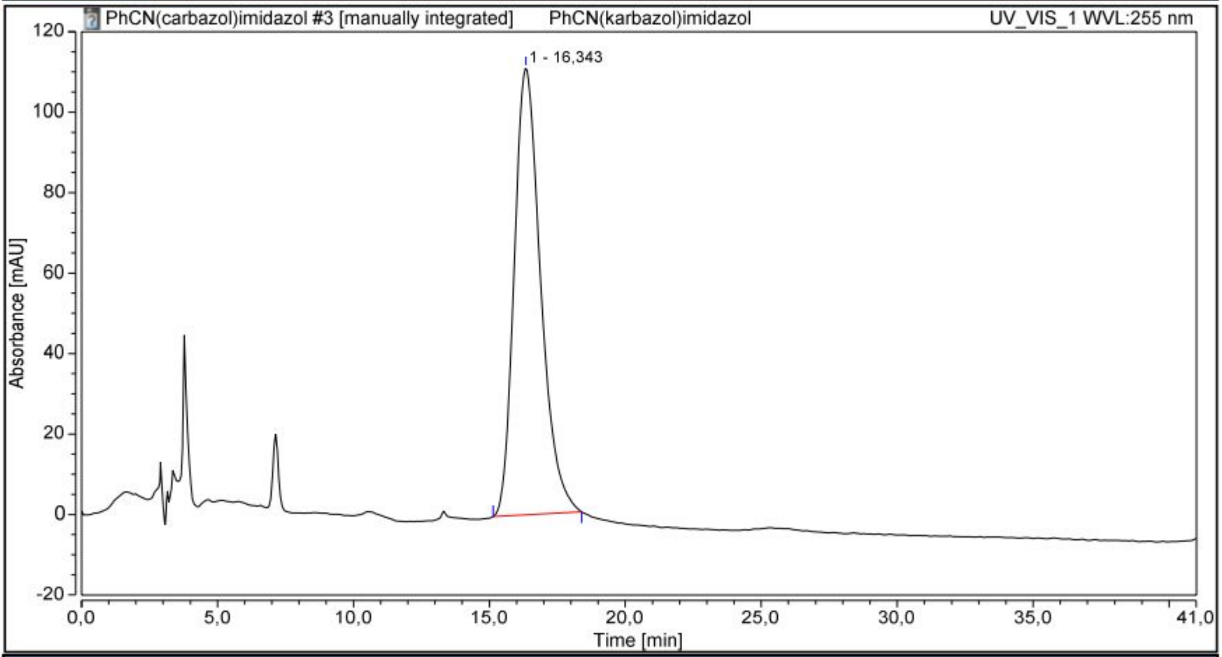

Integration Results

No.	Peak Name	Retention Time min	Area mAU*min	Height mAU	Relative Area %	Relative Height %	Amount n.a.
1		16,343	125,098	111,107	100,00	100,00	n.a.
**Total:**			**125,098**	**111,107**	**100,00**	**100,00**	

Figure S45 HPLC chromatogram of (*P*)-**3-CN**. Chiralpak® IC, Heptane/DCM/MTBE (70:20:30).

Chromatogram

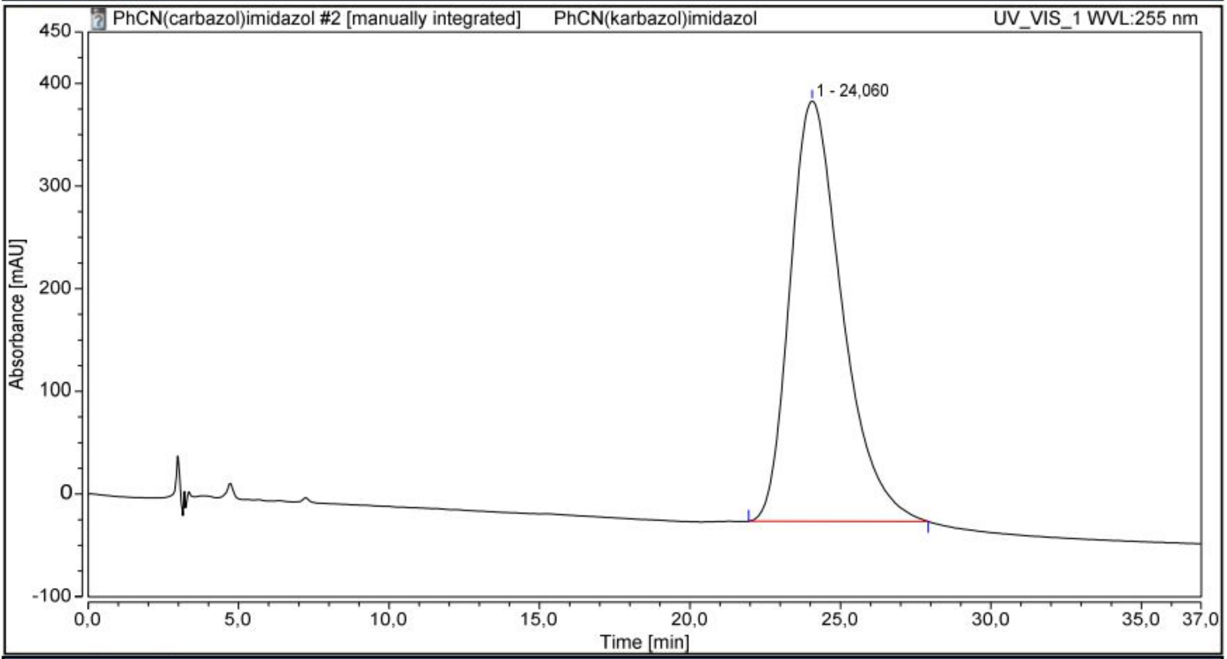

Integration Results

No.	Peak Name	Retention Time min	Area mAU*min	Height mAU	Relative Area %	Relative Height %	Amount n.a.
1		24,060	821,990	409,383	100,00	100,00	n.a.
**Total:**			**821,990**	**409,383**	**100,00**	**100,00**	

Figure S46 HPLC chromatogram of (*M*)-**3-CN**. Chiralpak® IC, Heptane/DCM/MTBE (70:20:30).

9. Crystallographic data

X-ray data of **1-H**: $C_{43}H_{26}N_2$, M=570.66 g.mol^{-1}, orthorhombic system, space group *P 2₁2₁2₁*, a=8.9605(5), b=14.6717(8), c=22.0436(12) Å, Z=4, V=2898.0(3) Å^3, Dc=1.31 g.cm^{-3}, μ(Cu Kα)=0.584 mm^{-1}, T=100 K, crystal dimensions of 0.05 x 0.05 x 0.36 mm, yellow bar. The independent part of the lattice cell is formed by one molecules of **1-H** (Figure S47). The structure model converged to the final R=0.0295 and R_w=0.0762 using 6239 independent reflections for 406 refined parameters (θ_{max}=79.86°). The Flack parameter converged to 0.36(8) indicating a possible lamellar twining which is common for helicenes.[8] CCDC registration number 2514620.

X-ray data of **2-H**: $C_{55}H_{35}N_3{\cdot}CHCl_3$, M=857.23 g.mol^{-1}, triclinic system, space group *P-1*, a=10.1657(8), b=11.9611(8), c=18.4084(14) Å, α=107.582(2), β=100.129(3), γ=97.231(2) °, Z=2, V=2062.0(3) Å^3, Dc=1.381 g.cm^{-3}, μ(Mo Kα)=0.268 mm^{-1}, T=100 K, crystal dimensions of 0.12 x 0.32 x 0.36 mm, light yellow plate. The independent part of the lattice cell is formed by one molecule of **2-H** and one solvent molecule (Figure S48). It was inevitable to model a disorder of two aromatic units at the side chain. The structure converged to the final R=0.0453 and R_w=0.1243 using 9139 independent reflections for 599 refined parameters (θ_{max}=28.30°). CCDC registration number 2514623.

X-ray data of **3-H**: $C_{55}H_{33}N_3{\cdot}CH_2Cl_2$, M=778.30 g.mol^{-1}, monoclinic system, space group *P2₁/c*, a=15.9828(12), b=8.7637(6), c=28.135(2) Å, β=93.174(3)°, Z=4, V=3934.8(5) Å^3, Dc=1.314 g.cm^{-3}, μ(Mo Kα)=0.142 mm^{-1}, T=100 K, crystal dimensions of 0.11 x 0.24 x 0.26 mm, yellow plate. The independent part of the lattice cell is formed by one molecule of **3-H** (Figure S49) and one disordered solvent molecule. The structure converged to the final R=0.0858 and R_w=0.2091 using 9431 independent reflections for 572 refined parameters (θ_{max}=28.33°). CCDC registration number 2514621.

X-ray data of **1-CN**: $2(C_{44}H_{25}N_3){\cdot}CHCl_3$, M=1310.70 g.mol^{-1}, monoclinic system, space group *P2₁/n*, a=21.8898(12), b=9.1054(7), c=31.988(2) Å, β=94.719(3)°, Z=4, V=6354.1(8) Å^3, Dc=1.370 g.cm^{-3}, μ(Mo Kα)=0.202 mm^{-1}, T=100 K, crystal dimensions of 0.10 x 0.32 x 0.34 mm, yellow plate. The independent part of the lattice cell is formed by two molecules of **1-CN** (Figure S50) and one disordered solvent molecule. The structure converged to the final R=0.0496 and R_w=0.1263 using 13837 independent reflections for 891 refined parameters (θ_{max}=28.30°). CCDC registration number 2514622.

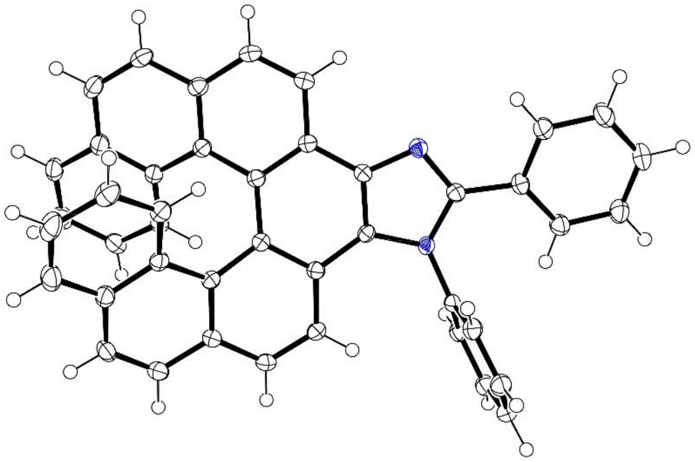

Figure S47 ORTEP projection of the crystal structure of **1-H**.

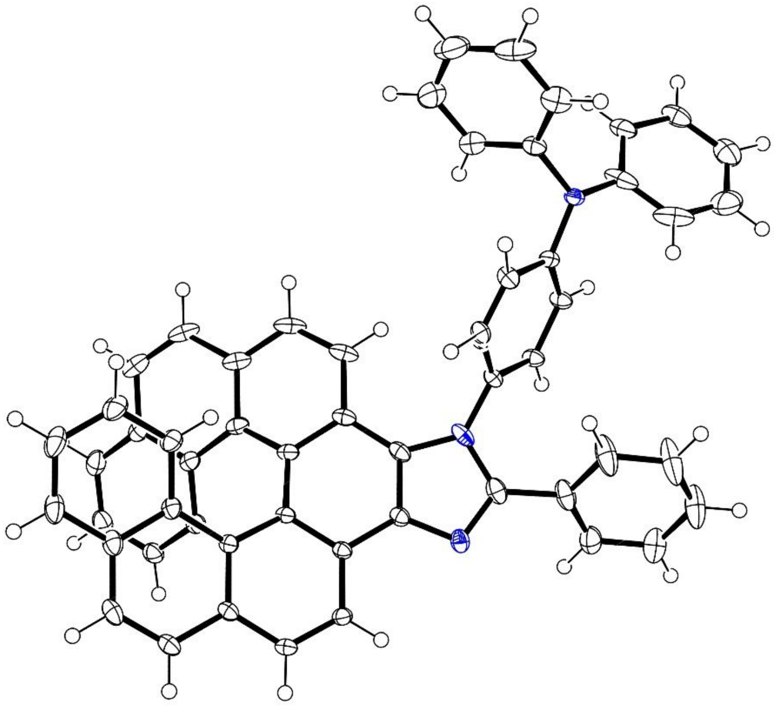

Figure S48 ORTEP projection of the crystal structure of **2-H**. The disordered parts of the molecule and the solvent were removed for the clarity.

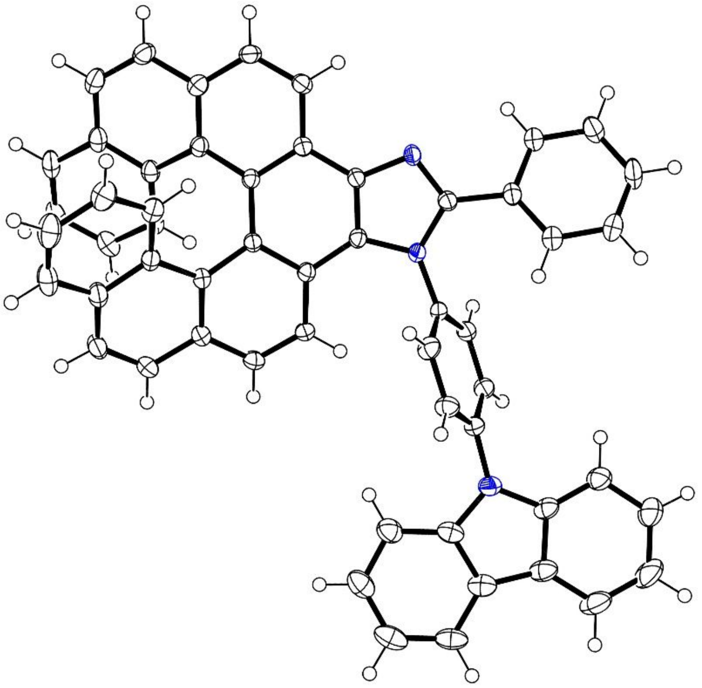

Figure S49 ORTEP projection of the crystal structure of **3-H**.

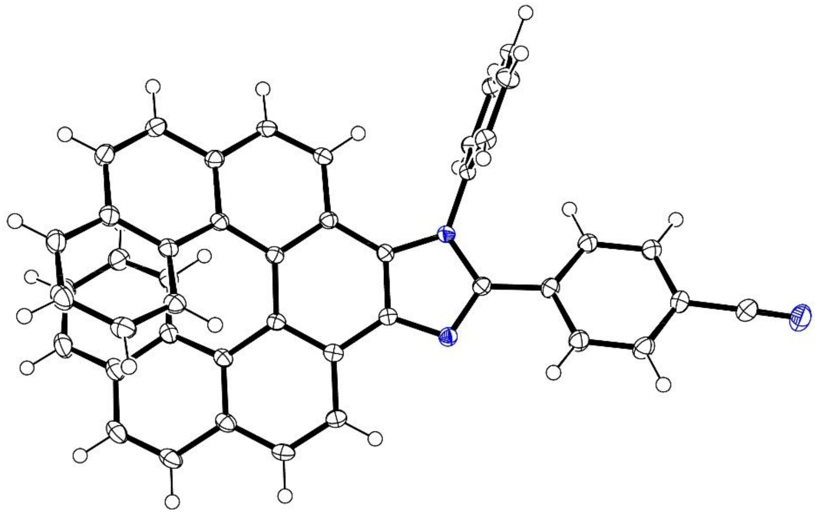

Figure S50 ORTEP projection of the crystal structure of **1-CN**.

10. References

(1) Sheldrick, G. M. Research Papers SHELXT – Integrated Space-Group and Crystal- Structure Determination Research Papers. *Acta Crystallogr. Sect. A* **2014**, *A71*, 3–8. https://doi.org/10.1107/S2053273314026370.

(2) Sheldrick, G. M. Crystal Structure Refinement with SHELXL. *Acta Crystallogr. Sect. C Struct. Chem.* **2015**, *71* (1), 3–8. https://doi.org/10.1107/S2053229614024218.

(3) Farrugia, L. J. ORTEP -3 for Windows - a Version of ORTEP -III with a Graphical User Interface (GUI). *J. Appl. Crystallogr.* **1997**, *30* (5), 565–565. https://doi.org/10.1107/S0021889897003117.

(4) Farrugia, L. J. WinGX and ORTEP for Windows : An Update. *J. Appl. Crystallogr.* **2012**, *45*, 849–854. https://doi.org/10.1107/S0021889812029111.

(5) Frisch, M. J.; Trucks, G. W.; Schlegel, H. B.; Scuseria, G. E.; Robb, M. A.; Cheeseman, J. R.; Scalmani, G.; Barone, V.; Mennucci, B.; Petersson, G. A.; Nakatsuji, H.; Caricato, M.; Li, X.; Hratchian, H. P.; Izmaylov, A. F.; Bloino, J.; Zheng, G.; Sonnenberg, J. L.; Hada, M.; et al. Gaussian 09, Revision A.02. Gaussian , Inc., Wallingford, CT 2009.

(6) Becke, A. D. Density-functional Thermochemistry. III. The Role of Exact Exchange. *J. Chem. Phys.* **1993**, *98* (7), 5648–5652. https://doi.org/10.1063/1.464913.

(7) Perdew, J. P.; Wang, Y. Accurate and Simple Analytic Representation of the Electron-Gas Correlation Energy. *Phys. Rev. B* **1992**, *45* (23), 13244–13249. https://doi.org/10.1103/PhysRevB.45.13244.

(8) Green, B. S.; Knossow, M. Lamellar Twinning Explains the Nearly Racemic Composition of Chiral, Single Crystals of Hexahelicene. *Science* **1981**, *214* (4522), 795–797. https://doi.org/10.1126/science.214.4522.795.
